# The involvement of the Stat1/Nrf2 pathway in exacerbating Crizotinib-induced liver injury: implications for ferroptosis

**DOI:** 10.1038/s41419-024-06993-z

**Published:** 2024-08-19

**Authors:** Lin Guo, JiaTing Ma, MingXuan Xiao, JiaYi Liu, ZhiYu Hu, Shuang Xia, Ning Li, Yan Yang, Hui Gong, Yang Xi, Rao Fu, Pei Jiang, ChunGuang Xia, Volker M. Lauschke, Miao Yan

**Affiliations:** 1 Department of Pharmacy, the Second Xiangya Hospital, Central South University, Changsha, China; 2 Institute of Clinical Pharmacy, Central South University, Changsha, China; 3 International Research Center for Precision Medicine, Transformative Technology and Software Services, Hunan, China; 4 Xiangya School of Pharmaceutical Sciences, Central South University, Changsha, China; 5 Department of Pharmacy, Wuzhou Gongren Hospital, Wuzhou, China; 6 Department of Pharmacy, Jining No 1 People’s Hospital, Jining Medical University, Jining, China; 7 Chia Tai Tianqing Pharmaceutical Group Co. Ltd, Lianyungang, Jiangsu China; 8 Department of Physiology and Pharmacology, Section of Pharmacogenetics, Karolinska Institutet, SE-171 77 Stockholm, Sweden

**Keywords:** Diseases, Pathogenesis

## Abstract

Crizotinib carries an FDA hepatotoxicity warning, yet analysis of the FAERS database suggests that the severity of its hepatotoxicity risks, including progression to hepatitis and liver failure, might be underreported. However, the underlying mechanism remains poorly understood, and effective intervention strategies are lacking. Here, mRNA-sequencing analysis, along with KEGG and GO analyses, revealed that DEGs linked to Crizotinib-induced hepatotoxicity predominantly associate with the ferroptosis pathway which was identified as the principal mechanism behind Crizotinib-induced hepatocyte death. Furthermore, we found that ferroptosis inhibitors, namely Ferrostatin-1 and Deferoxamine mesylate, significantly reduced Crizotinib-induced hepatotoxicity and ferroptosis in both in vivo and in vitro settings. We have also discovered that overexpression of AAV8-mediated Nrf2 could mitigate Crizotinib-induced hepatotoxicity and ferroptosis in vivo by restoring the imbalance in glutathione metabolism, iron homeostasis, and lipid peroxidation. Additionally, both Stat1 deficiency and the Stat1 inhibitor NSC118218 were found to reduce Crizotinib-induced ferroptosis. Mechanistically, Crizotinib induces the phosphorylation of Stat1 at Ser727 but not Tyr701, promoting the transcriptional inhibition of Nrf2 expression after its entry into the nucleus to promote ferroptosis. Meanwhile, we found that MgIG and GA protected against hepatotoxicity to counteract ferroptosis without affecting or compromising the anti-cancer activity of Crizotinib, with a mechanism potentially related to the Stat1/Nrf2 pathway. Overall, our findings identify that the phosphorylation activation of Stat1 Ser727, rather than Tyr701, promotes ferroptosis through transcriptional inhibition of Nrf2, and highlight MgIG and GA as potential therapeutic approaches to enhance the safety of Crizotinib-based cancer therapy.

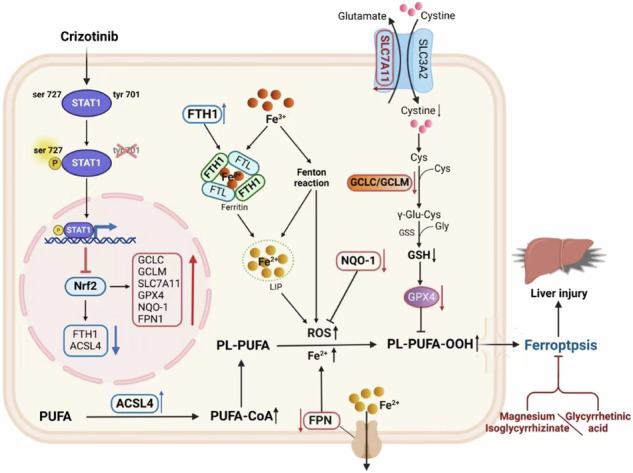

## Highlights

- The primary mechanism of hepatocyte death induced by Crizotinib is through ferroptosis, and the utilization of ferroptosis inhibitors effectively reverses Crizotinib-induced hepatotoxicity.
- Crizotinib triggers the phosphorylation of Stat1 at Ser727 rather than Tyr701, which facilitates the transcriptional suppression of Nrf2 expression upon nuclear entry, promoting ferroptosis, and leading to liver toxicity.
- Both MgIG and GA demonstrate the ability to mitigate Crizotinib-induced hepatotoxicity without impacting its anti-tumor effectiveness.

## Introduction

Crizotinib, the first tyrosine kinase inhibitor (TKI) globally to target anaplastic lymphoma kinase (ALK), mesenchymal–epithelial transition factor (Met), and proto-oncogene 1 tyrosine kinase (ROS1), remains the sole TKI approved for the targeting of both ALK and ROS1. Widely utilized as first-line therapy for locally advanced or metastatic ALK-positive non-small cell lung cancer (NSCLC) [1], Crizotinib, known as one of the “legend drugs” due to its rapid development-to-market timeline, has demonstrated significant clinical efficacy and plays a pioneering role in the treatment of anti-NSCLC [2]. However, the FDA issued a “Warnings and Precautions” labeling regarding severe hepatotoxicity shortly after the approval of Crizotinib. Recent literature suggests that 68% of Chinese cancer patients treated with Crizotinib experience elevated serum aminotransferases, with a higher prevalence observed in Chinese patients compared to non-Chinese patients [3, 4]. Additionally, data indicates that transaminases elevation is one of the most common grade 3/4 adverse events following Crizotinib therapy, with 8% of patients experiencing this and 1.3% permanently discontinuing treatment due to hepatotoxicity [3]. The etiology of Crizotinib-induced hepatotoxicity remains uncertain, and there is a deficiency in targeted clinical management approaches. Consequently, it is crucial to investigate the molecular mechanisms of Crizotinib-induced hepatotoxicity and pinpoint key toxicity targets.

Excessive hepatocyte mortality in the liver is a significant pathophysiological characteristic and prevalent outcome of drug-induced hepatotoxicity, contributing to acute liver failure (ALF) [5]. The types of cellular demise vary among hepatotoxic substances, necessitating the inhibition of specific death pathways. Ferroptosis, a recently discovered form of regulated cell death, distinguishes itself from apoptosis, necrosis, and autophagy [6]. This iron-dependent process is triggered by lipid peroxidation and has been implicated in the pathogenesis of several liver diseases, including hematochromatosis, alcoholic liver disease, hepatitis C virus infection, non-alcoholic fatty liver disease, hepatocellular carcinoma, and drug-induced liver injury (DILI), as evidenced by a growing body of research [7–10]. Inhibition of ferroptosis through ferrostatin-1 (Fer-1) or deferoxamine mesylate (DFO) effectively prevents acetaminophen-induced ALF, suggesting that ferroptosis, a promising therapeutic target compared to inflammation, is the predominant mechanism of hepatocyte death [11**–**13]. Thus, ferroptosis emerges as a significant contributor to hepatocyte death in DILI.

Nuclear factor erythroid 2-related factor 2 (Nrf2) serves as the primary transcriptional regulator of antioxidant responses and is crucial in maintaining redox homeostasis [14, 15]. Various pathological conditions are characterized by disrupted redox balances, such as ferroptosis induced by lipid peroxidation [16, 17]. The principal inhibitory systems of ferroptosis, which include the cystine/GSH/GPX4 axis, NAD(P)H/FSP1/CoQ10 system, and GCH1/BH4/DHFR system, have key transcriptional targets that are regulated by Nrf2 [18]. This underscores the significant role of Nrf2 in governing the ferroptosis network. Meanwhile, recent studies have demonstrated the involvement of the Nrf2 pathway in drug-induced toxicities [19**–**22]. However, its precise role in DILI remains unclear, with questions arising as to whether Nrf2 is upregulated in response to stress to mitigate toxic effects, or if it is hindered from nuclear translocation, thereby impeding the activation of downstream antioxidant target genes and contributing to hepatotoxicity. The investigation of the regulatory function of Nrf2 in drug toxicity continues to be a compelling topic.

Stat1, a key member of the Stat family, is involved in a wide range of physiological and pathological processes, including cell proliferation, cell death, and immune regulation. Recent research has highlighted its significant contributions to the development of liver diseases such as hepatic ischemia-reperfusion injury and DILI [23]. In physiological circumstances, Stat1 is typically localized within the cytoplasm. Upon exposure to diverse exogenous or endogenous stimuli, Stat1 undergoes phosphorylation, leading to the formation of homodimers and heterodimers. Subsequently, the phosphorylated Stat1 translocates to the nucleus to carry out its essential biological functions. Recent scholarly works indicate that the inhibition of Stat1 by Fludarabine (NSC118218) can potentially reverse the interferon γ-induced suppression of SLC7A11 and GPX4 proteins, implying a potential role for Stat1 in mediating ferroptosis through the regulation of the SLC7A11/GPX4 pathway [24]. However, the functional phosphorylation sites of Stat1, the mechanisms by which Stat1 regulates ferroptosis, and the regulatory interactions between Stat1 and Nrf2 remain unclear and warrant further investigation.

In this research, the examination of the US Food and Drug Administration Adverse Event Reporting System (FAERS) database uncovered a possible underestimation of the “Warnings and Precautions” labeling for Crizotinib-induced hepatotoxicity. The primary mode of Crizotinib-induced hepatocyte death was identified as ferroptosis. Through mechanistic analysis, it was determined that AAV8-mediated overexpression of Nrf2 alleviated Crizotinib-induced hepatotoxicity and ferroptosis by correcting imbalances in glutathione metabolism, iron homeostasis, and lipid peroxidation, as indicated by integrated transcriptomic analyses. Stat1 phosphorylation at Ser727 played a crucial role in facilitating liver ferroptosis upon Crizotinib treatment through transcriptional Nrf2 inhibition. Ultimately, the genetic or pharmacological inhibition of Stat1 expression using Glycyrrhetinic acid (GA) and magnesium isoglycyrrhizinate (MgIG) represents a promising therapeutic approach for Crizotinib-induced hepatotoxicity.

## Method

### Cell culture and materials

The cells were cultured in a 37 °C humidified incubator with 5% CO_2_. AML12 and HL7702 cell lines were maintained in DMEM (Gibco) supplemented with 10% FBS (Gibco) and 1% penicillin–streptomycin. NCI-H2228 and NCI-3122 cell lines (obtained from Servicebio Biological) were cultured in RPMI1640 (Gibco) with 10% FBS (Gibco) and 1% penicillin–streptomycin. AML12 cells were sourced from BNCC, while HL7702 cells were provided by Shanghai Zhong Qiao Xin Zhou Biotech Co., Ltd. Cells were regularly tested and verified to be mycoplasma negative.

Crizotinib (Crizo, MB2025) was provided by Meilunbio (Dalian, China), while Z-VAD-FMK (HY-16658B), Necrostatin-1 (Nec1, HY15760) and Belnacasan (VX765, HY-13205) were purchased from MedChemExpress (NJ, USA). Fer1 (S7243), DFO (S5742), chloroquine (CQ, S6999), 3-methyl adenine (3-MA, S2767), Olaparib (S1060), NSC118218 (S1491), and Glumetinib (SCC244, S8676) were purchased from Selleck Chemicals (Houston, USA). tBHQ (IT1150) was acquired from Solarbio (Beijing, China). Liquiritin (LQ, BP0874) and Glycyrrhizin (GL, BP2052) were purchased from Chengdu Biopurify Phytochemicals, China). GA (A19175) was obtained from Xiya Reagent Co., Ltd (Shandong, China). Isoliquiritigenin (ISL, 150928) was obtained from Changsha OnRoad Biotech Co., Ltd. (Hunan, China). MgIG was obtained from Zhengda Tianqing Pharmaceutical Group Co., Ltd. (Jiangsu, China).

### Data source from FAERS, study design, and signal detection

The data utilized in this research was obtained from the AERSMine website [25]. As of June 2022, the FDA has sanctioned 56 TKIs. This investigation incorporated 15 TKIs for the treatment of NSCLC and 6 TKIs with FDA-boxed warnings for hepatotoxicity, covering hepatotoxicity-related adverse events in cancer patients treated with the aforementioned TKIs from the first quarter of 2004 to the fourth quarter of 2022. TKIs with fewer than 3 reports were excluded from the analysis, such as mobocertinib. Due to the potential impact of co-administered drugs on signal associations between TKIs and hepatotoxicity, medications identified as having the highest potential for DILI in the DILIrank database [26] were omitted from the analysis.

The present study conducted a case/non-case analysis (detailed in Table S1) to assess the signal values for drug-related hepatic disorders—a comprehensive search related to the specified TKIs in comparison to those of other drugs in the FAERS database.

Subsequently, we conducted a detailed examination and analysis of signals pertaining to hepatotoxicity at the level of various preferred terms (PTs), which were then classified into system organ classes to provide a more comprehensive understanding of the distinct AEs signals associated with TKIs. 9 PTs were identified for inclusion in the analysis, encompassing a range of hepatotoxic effects, such as increased transaminases, DILI, hepatocellular injury, cholestasis, hepatic failure, hepatitis, acute hepatitis, and fulminant hepatitis. The information component (IC), a Bayesian metric [27], was utilized to quantify the strength of these signals. When the lower bound of the 95% confidence interval of the IC (IC_025_) is >0, a signal association between the drug and specific AE may exist. Larger IC_025_ values indicate stronger associations.

### Animal experiments

The experimental procedures and methods involving mice were conducted in accordance with the guidelines approved by the Ethics Committee of the Second Xiangya Hospital of Central South University (Hunan, China) and ARRIVE guidelines (https://arriveguidelines.org). C57BL/6 male mice (weighing 18–22 g) were procured from Hunan Slack Jingda Experimental Animal Co., Ltd. (Hunan, China). Subsequently, they were randomly allocated to diverse enclosures (with each enclosure constituting a group), and each group was marked, respectively, and granted unrestricted access to the general laboratory diet and water. A one-week acclimatization period was observed prior to the commencement of experiments to allow the animals to adjust to the laboratory conditions. Prior to sample collection, mice underwent a 12-h fasting period.

Investigators were not blind to treatment groups. In the investigation of Crizotinib-induced hepatotoxicity, Crizotinib was solubilized in 0.5% carboxymethyl cellulose sodium (CMC-Na) and administered to mice at a dosage of 120 mg/kg/day for a duration of 3 weeks via gavage (*n* = 5–8 per group). To assess the potential therapeutic effects of Fer1 and DFO on Crizotinib-induced ferroptosis and liver damage, male mice were treated with 120 mg/kg/day Crizotinib and/or received intraperitoneal injections of 1 mg/kg/day Fer1 or 30 mg/kg/day DFO for a period of 3 weeks (*n* = 8 per group). To assess the therapeutic potential of GA and MgIG in mitigating Crizotinib-induced liver injury, male mice were administered 120 mg/kg/day of Crizotinib and/or intraperitoneal injections of 5 mg/kg/day GA or 10 mg/kg/day MgIG for a duration of 3 weeks (*n* = 8 per group).

Additionally, overexpression of Nrf2 in mice was achieved through the use of AAV8, which was constructed and packaged by HanBio Biotechnology (Shanghai, China). Subsequently, AAV8-control (1 × 10^12^ vg/mL) and AAV8-Nrf2 (1 × 10^13^ vg/mL) were administered via tail vein injection to C57 male mice (*n* = 8 per group). 4 weeks later, mice with overexpression of Nrf2 were treated with 120 mg/kg/day Crizotinib for 3 weeks. Then, Serum was collected for the measurement of alanine aminotransferase (ALT), aspartate aminotransferase (AST), and alkaline phosphatase (ALP) levels by the automated clinical analyzer (7600, HITACHI Ltd., Tokyo, Japan), and liver was harvested for H&E staining.

### mRNA sequencing analysis

RNA Isolation and Library Preparation were performed, then the libraries were sequenced by OE Biotech, Inc. (Shanghai, China) on a lllumina Novaseq 6000 platform, and 150 bp paired-end reads were generated. Raw reads of fastq format were firstly processed using fastp and the low-quality reads were removed to obtain the clean reads. The clean reads were mapped to the reference genome using HISAT2. FPKM of each gene was calculated and the read counts of each gene were obtained by HTSeq-count. PCA analysis was performed using R (v 3.2.0) to evaluate the biological duplication of samples. Differential expression analysis was performed using the DESeq2. *q* < 0.05 and |log2(fold-change)| > 1 was set as the threshold for significantly differential expression genes (DEGs). Based on the hypergeometric distribution, GO, KEGG pathway, Reactome and Wiki Pathways enrichment analysis of DEGs were performed to screen the significant enriched term using R (version 3.2.0), respectively.

### Cell viability assay

The cell viability assay was conducted in accordance with previously published protocols [21]. Briefly, treated cells were incubated with fresh MTT solution (100 μL/well; stock concentration of 5 mg/mL in PBS) for a duration of 3–4 h. After the crystal dissolved, the plates were analyzed using an automated microplate spectrophotometer (Thermo Multiskan Spectrum, Thermo Electron Corporation, USA) to measure absorbance at 490 nm.

### Real-time quantitative polymerase chain reaction (RT- qPCR)

Tissue and cell samples were collected post-treatment, and total RNA was extracted using Trizol reagent (AG21102, Agbio) with subsequent cDNA synthesis and PCR amplification in accordance with the supplier’s protocols with a QuantStudio ^TM^ 5 Detection System (Thermo, USA). Then, the relative mRNA expression of genes was calculated and analyzed by using the 2^−∆∆Ct^ method, and GAPDH was used for normalization. The primer sequences of the target genes in this study are provided in Table S2.

### Determination of iron, antioxidant enzymes, malondialdehyde (MDA) and peroxidation (LPO)

Following pretreatment of hepatic tissues or cells, measurements were taken for total iron content, total glutathione (TGSH), reduced glutathione, oxidized glutathione (GSSG), Cysteine (Cys), LPO, and MDA levels by using corresponding commercial kits (Jiancheng Bioengineering Institute, Nanjing, China) according to the manufacturer’s protocol, respectively.

### Transmission electron microscope (TEM)

AML12 cells were collected via centrifugation at 1000 rpm for 5 min, fixed with electron microscope fixation solution at 4 °C for 2 h, and then washed 6 times with 0.1 M phosphate buffer. The cells were treated with 1% osmium acid, followed by fixation with 1% osmium tetroxide and dehydration through an alcohol gradient prior to embedding in resin. The samples underwent detection using transmission electron microscopy (Hitachi, Tokyo, Japan) after double staining with uranium acetate and lead citrate.

### Measurement of intracellular Fe^2+^

The fluorescent probe FerroOrange (Dojindo, Japan) was utilized for the detection of intracellular Fe^2+^. In AML12 cells following treatment according to the experimental protocol. Initially, Cells were incubated with DAPI for 10 min to facilitate nuclear staining. Subsequently, cells were rinsed with PBS and exposed to FerroOrange (0.1 μM) for 5 min at 37 °C before being promptly imaged using a fluorescence microscope (Olympus, Japan).

### Transfection of siRNA and plasmid

Cells were seeded into six-well plates and grown to 50–60% confluence. Transfection was performed using Lipofectamine 2000 (Invitrogen, USA), siRNA, and plasmid according to the manufacturer’s recommendations. For Nrf2 and Stat1, full-length Nrf2 and Stat1 were cloned into pcDNA3.1-flag vectors. One-site mutant plasmids of Stat1 Ser727A-pcDNA3.0-flag, Stat1 Tyr701A-pcDNA3.0-flag, and Stat1 Tyr701F-pcDNA3.0-flag were generated. siRNA was transfected at a final concentration of 100 nM. The cell culture solution was changed to a complete medium at 6 h after transfection. Then, the cells were treated with drugs for the indicated times, and treated or untreated cells were collected for further analysis. The siRNA sequences are provided in Table S3.

### Iron ion concentration detection by ICP–MS

Liver tissue samples weighing 50 mg were placed in a 50 mL constant-volume tube, to which 1 mL of concentrated nitric acid was added. The tube was then placed on a heating plate and heated at 130 °C for 2 h. Subsequently, the dissolved sample was allowed to cool to room temperature, and ultra-pure water was added to achieve a final volume of 20 mL. Liver iron concentration was measured using ICP–MS (NexION 1000G, PerkinElmer, USA), with the operating parameters of the instrument detailed in Table S4.

### Measurement of lipid ROS by flow cytometry

The AML12 cells, both treated and untreated, were exposed to 10 μM C11-BODIPY (581/591) (Life Technologies) for 30 min at 37 °C. Subsequently, the cells were rinsed three times with PBS and dissociated using trypsin (without EDTA, NCM Biotech, Shanghai, China). Fluorescence intensity was quantified through the concurrent detection of green (484/510 nm) and red signals (581/610 nm) utilizing a CytoFLEX flow cytometer (Beckman Coulter, CA, USA).

### Immunohistochemistry

The paraffin-embedded tissue sections underwent dewaxing, antigen retrieval, and serum blocking before being incubated with primary antibodies (4-HNE, ab46545, Abcam) overnight at 4 °C. Subsequently, a Biotinylated goat anti-rabbit secondary antibody solution was applied, followed by DAB and hematoxylin staining for visualization of the cell nucleus. Following dehydration and mounting, the sections were examined under an optical microscope (Olympus, Japan).

### Immunofluorescence staining

Following dewaxing of the paraffin section of the liver, antigen retrieval was conducted prior to blocking in 3% BSA for 30 min. Subsequently, the samples were incubated with primary antibodies at 4 °C overnight and HRP-conjugated secondary antibodies at room temperature for 50 min, targeting pStat1 Ser727 and Nrf2. The nuclei in the samples were then stained with DAPI for visualization, followed by self-fluorescence quenching and sealing. Imaging was performed using a Nikon Eclipse C1 microscope.

### Western blot

Total and nuclear protein extracts from HL7702, AML12 cells, and mouse liver tissues were prepared as described in our previous study [21, 28]. Protein extract was separated in 8–12% SDS–PAGE and electrophoretically transferred onto PVDF membranes. Membranes were blocked with 5% skim milk in 1× TPBS and then incubated with the antibodies listed below. After primary antibody incubation at 4 °C overnight, membranes were incubated with secondary antibodies and high-sensitivity ECL reagents (P2200, NCM biotech).

The following primary antibodies were used: anti-ACSL4 (df12141, Affinity), anti-GPX4 (ab125066, Abcom), anti-SLC7A11 (ab175186, Abcom), anti-P53 (a0263, Abclonal), anti-Nrf2 (af0639, Affinity), anti-HO1 (10701-1-AP, Proteintech), anti-Stat1 (TU376539, Abmart), anti-pStat1 Tyr701 (TA3300, Abmart), anti- pStat1 Ser727 (ab109461, Abcom), anti-Met (ER40501, Huabio), anti-pMet (PA2979, Abmart), anti-Histone H3 (af0863, Affinity), anti-β-actin (ac026, ABclonal) and anti-GAPDH (a19056, ABclonal).

### Luciferase assay

The pGL3-basic vectors containing 3.0 kb upstream of the core coding region of the specified genes, Luc-TK, and overexpression plasmids were transfected into AML12 cells in 24-well plates using Lipofectamine 2000. Following transfection for 48 h with or without Crizotinib, the cells were harvested, and the Dual-Luciferase Reporter Assay (Promega) was utilized to measure the luciferase activity of each group according to the manufacturer’s instructions. Renilla luciferase activity was referred to as an internal control to calculate the relative luciferase activity.

### Chromatin immunoprecipitation (CHIP) assay

A total of 107 AML12 cells with or without Stat1 overexpression were treated with 1% formaldehyde for 10 min to crosslink DNA and then incubated with 0.2 g glycine for 5 min to stop the reaction. Then, after being washed three times with ice-cold PBS, the cells were harvested. Then nuclei preparation was performed. Thereafter, the genomic DNA was fragmented into 300–100 bp lengths for 15 min by sonication (230 W, 2 × 5 s intervals) and collected supernatant for pre-washing. Then, lysates were incubated with anti-STAT1 or anti-flag antibody (normal IgG was used as a control) at 4 °C overnight. The subsequent day, the prepared Protein A/G-beads were added, and the immunocomplexes were incubated at room temperature for 30 min before washing with wash buffer and TE wash buffer. After elution and reverse crosslinking, Precipitate and purify DNA, which was analyzed using qPCR. Primers for ChIP-qPCR are listed in Table S5.

### Statistical analysis

The data were presented as the means ± SEM. The significance of differences between groups was determined with the one-way analysis of variance (ANOVA) and SPSS 20.0 software (SPSS Inc., Chicago, IL, USA), and a comparison between two groups was made with an independent sample *t*-test. Figures were drawn with GraphPad Prism 9 (GraphPad Software, La Jolla, CA, USA).

## Result

### Potential underestimation of the “Warnings and Precautions” labeling for Crizotinib-induced hepatotoxicity

**Fig. 1:**
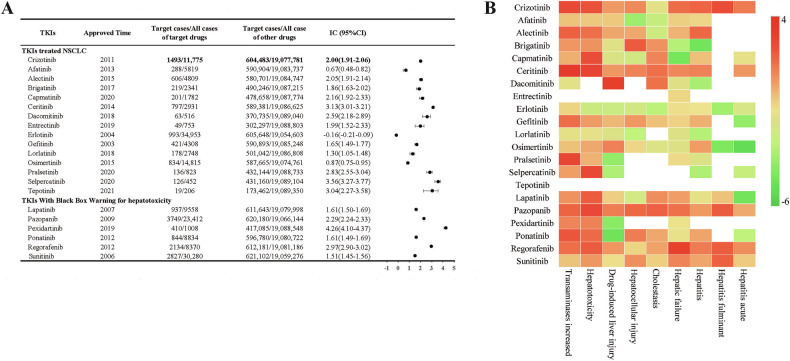
Signal association plots of hepatotoxicity and selected key hepatotoxicity PTs were analyzed for various TKIs in the FAERS database. **A** Association map of different TKIs with reported hepatotoxicity signals. **B** Heatmap of the association between different TKIs and selected key hepatotoxicity PTs signals. ALK anaplastic lymphoma kinase, ROS1 ROS proto-oncogene 1, cMET cellular-mesenchymal to epithelial transition factor, EGFR epithelial growth factor receptor, RET proto-oncogene tyrosine-protein kinase receptor ret, HER2 human epidermal growth factor receptor-2, VEGFR vascular endothelial growth factor receptor, CSF-1R colony-stimulating factor 1 receptor, KIT KIT proto-oncogene receptor tyrosine kinase, FLT3 FMS-like tyrosine kinase 3, RAF1 Raf-1 proto-oncogene, serine/threonine kinase, PDGFR platelet-derived growth factor receptors, ROR reporting odds ratio, 95%CI 95% confidence interval.

Figure 1A illustrates the presence of 21 TKIs with prominent liver toxicity warning signals, including 15 intended for the treatment of NSCLC and 6 carrying FDA black-boxed warnings for hepatotoxicity. Among these TKIs, Crizotinib demonstrated a higher IC_025_ value for hepatotoxicity compared to 6 out of 15 (7/15) NSCLC TKIs and half of the TKIs with black-boxed warnings. Subsequently, various significant PTs associated with liver toxicity, such as rapidly progressive and unfavorable clinical outcomes, were analyzed, as depicted in Fig. 1B. The IC_025_ value for cholestasis (11/21), a form of liver injury linked to Crizotinib, was found to be below 0, indicating a lack of statistically significant correlation. This finding contributed to the ongoing debate surrounding the types of liver injuries induced by Crizotinib. In comparison to the other 20 TKIs, Crizotinib demonstrated higher correlations with various PTs of liver injury, including increased transaminases (3/21), hepatotoxicity (5/21), and DILI (6/21), as well as specific types of liver injury such as hepatocellular injury (5/21), suggesting that further exploration of the mechanisms underlying Crizotinib-induced liver injury from a hepatocellular injury perspective might yield valuable insights. Particularly noteworthy were the strong associations observed between Crizotinib and liver failure (hepatic failure (2/21)) and hepatitis (hepatitis (1/21), fulminant hepatitis (1/21), acute hepatitis (1/21)), which aligned with results reported in several case studies [29**–**32]. The aforementioned findings indicated that the hepatotoxicity “Warnings and Precautions” labeling for Crizotinib might have been underestimated.

### Crizotinib caused imbalances in glutathione metabolism, iron homeostasis, and lipid peroxidation in hepatocytes

**Fig. 2:**
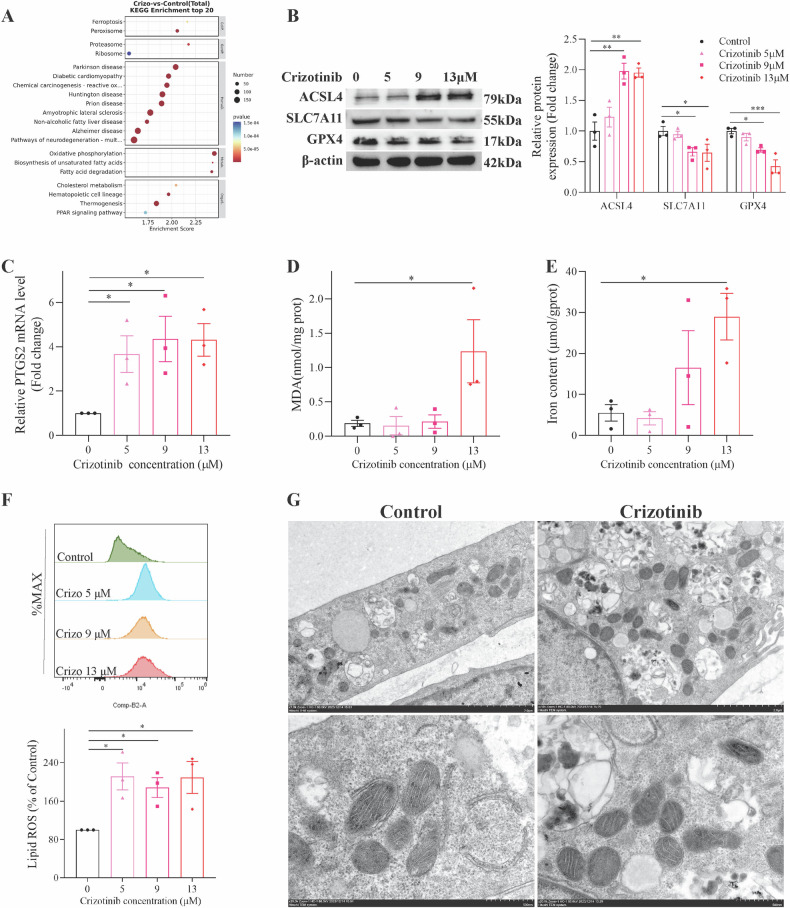
Crizotinib induced ferroptosis in AML12 cells (*n* = 3). **A** Signification enriched top 20 KEGG pathway for the DEGs between the control and Crizotinib group. **B** and **C** The changes of ACSL4, SLC7A11, GPX4 protein expression (**B**), and PTGS2 mRNA levels (**C**) in AML12 cells treated with Crizotinib in different concentrations for 48 h were observed. **D** and **E** Iron content (**D**) and MDA (**E**) were measured by reagent kit following treatment with increasing concentrations of Crizotinib for 48 h. **F** The induction of lipid ROS was determined by BODIPY™ 581/591 C_11_ and flow cytometry for 48 h. **G** Representative TEM images showed mitochondrial damages treated with 13 μM Crizotinib for 48 h in the AML12 cell. Scale bars, 2 μm (upper panel) and 500 nm (lower panel). *P < 0.05, **P < 0.01 and ***P < 0.001 vs. control group. Crizo Crizotinib.

Initially, an animal model was developed to mimic the hepatotoxic effects of Crizotinib observed in clinical settings. The levels of ALT, AST, and ALP, which serve as indicators of liver parenchymal damage, exhibited a notable alteration following treatment with 120 mg/kg/day Crizotinib (Fig. S1A–C). Furthermore, histological analysis using H&E staining (Fig. S1D) demonstrated that, in comparison to the control group, the hepatic lobule structure of mouse liver tissue exhibited indistinct features, disarrayed hepatic cords, loose cytoplasmic appearance, pronounced cytoplasmic and nuclear separation, and notable inflammatory cell infiltration in the Crizotinib group. These findings suggested the successful establishment of a Crizotinib-induced liver injury model. Subsequently, an in-depth investigation into the underlying mechanism of Crizotinib-induced hepatotoxicity was conducted through mRNA sequencing analysis. A total of 5569 DEGs, with 2577 upregulated and 2992 downregulated in the Crizotinib group, were identified (Fig. S2A–C). Furthermore, KEGG, GO, and WikiPathways enrichment analyses all revealed the ferroptosis pathway as a significantly enriched pathway among the DEGs (Figs. 2A and S2D, E).

Furthermore, ALK + NSCLC cell lines NCI-H2228 and NCI-H3122 were treated with Crizotinib for 48 h. In the concentration range of 0–30 μM, the cell viability decreased in a concentration-dependent manner, exhibiting significant anti-cancer effects (Fig. S3A, B). Subsequent validation of the toxic effects of Crizotinib within this concentration range was conducted in normal hepatocytes. The survival rates of the human hepatocyte AML12 and HL7702 cells were significantly reduced upon Crizotinib treatment in a concentration- and time-dependent manner (Fig. S3C, D). Consequently, A concentration of 5–15 μM and 48 h exposure will be used for further investigating the mechanisms of Crizotinib-induced hepatotoxicity. Crizotinib upregulated ACSL4 protein, a pivotal enzyme involved in lipid composition, while downregulating the ferroptosis key regulators SLC7A11 and GPX4 in AML12 (Fig. 2B) and HL7702 cells (Fig. S4A, B) dose-dependently. Further, Crizotinib markedly increased the mRNA levels of PTGS2, a recognized molecular marker of ferroptosis, by up to 4.3-fold in AML12 cells (Fig. 2C). Uncontrolled lipid peroxidation, which is reliant on iron, is a defining characteristic of ferroptosis. The results in Fig. 2D, E show that Crizotinib significantly increased the ferroptosis key indexes iron content and lipid peroxidation marker MDA in AML12 cells, consistent with the results in HL7702 cells (Fig. S4C, D). Following lipid peroxidation reactions, excessive lipid ROS accumulate and interact with lipid molecules on the cellular membrane, subsequently leading to lipid peroxidation and membrane impairment. Flow cytometric analysis with the BODIPYTM 581/591 C11 probe showed Crizotinib markedly increased lipid ROS (Fig. 2F). TEM revealed reduced mitochondrial size and cristae density and cellular membrane disruption after Crizotinib treatment in AML12 cells (Fig. 2G), indicating morphological features of ferroptosis. These findings suggest Crizotinib triggers ferroptosis in hepatic cells.

### Inhibiting ferroptosis alleviated Crizotinib-induced hepatotoxicity

**Fig. 3:**
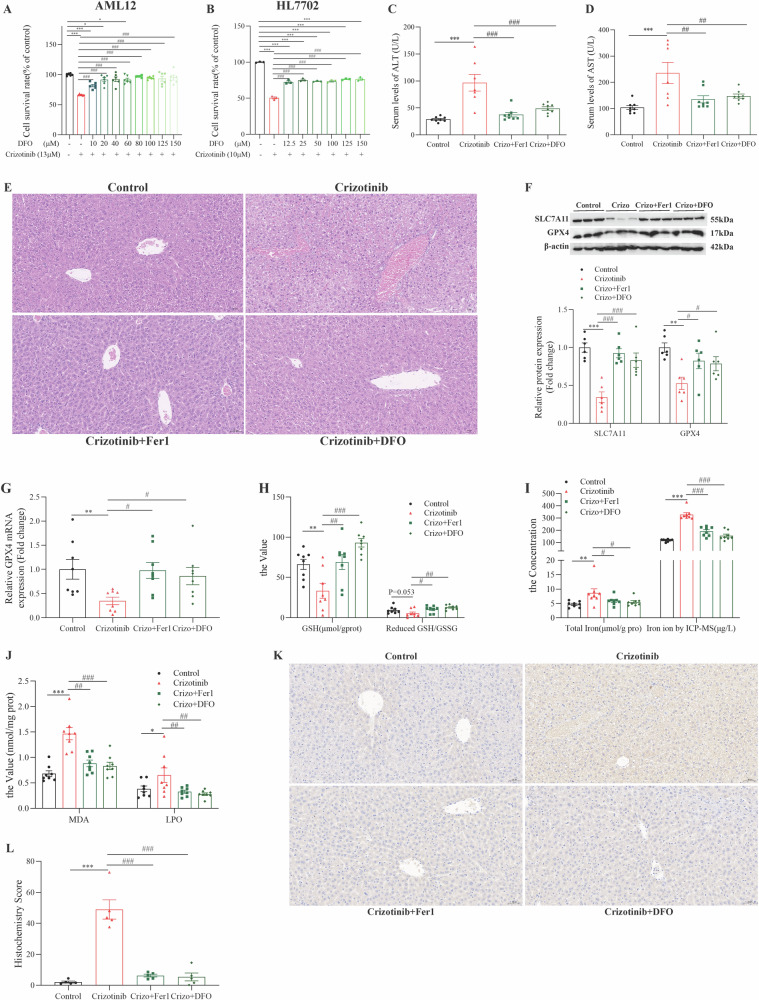
Fer1 and DFO alleviate Crizotinib-triggered hepatotoxicity and ferroptosis. **A** and **B** The survival rate of AML12 cells (**A**, *n* = 6) and HL-7702 cells (**B**, *n* = 3) treated with Crizotinib and/or DFO in different concentrations for 48 h. C57 male mice were treated with 120 mg/kg/day Crizotinib and/or 1 mg/kg/day Fer1 or 30 mg/kg/day DFO for 3 weeks. **C** and **D** The levels of serum ALT (**C**) and AST (**D**) were analyzed (*n* = 7–8). **E** Representative images of H&E staining in liver tissues (H&E staining, ×20). **F** The expression of SLC7A11 and GPX4 protein were measured by western blot (*n* = 6). **G** The GPX4 mRNA level was determined by RT-qPCR (*n* = 8). **H**–**J** GSH, the reduced GSH/HSSG (**H**, *n* = 8), total Iron concentration measured by reagent box and ICP-MS (**I**, *n* = 8), MDA and LPO (**J**, *n* = 8) were measured. **K** and **L** Representative pictures of 4-HNE expression in each group measured by immunohistochemical staining (**K**, ×20). And the staining outcomes were quantitatively assessed using the *H*-score system (**L**, *n* = 5). *P < 0.05, **P < 0.01 and ***P < 0.001 vs. control group. ^#^*P* < 0.05, ^##^*P* < 0.01 and ^###^*P* < 0.001 vs. Crizotinib group^.^ Crizo Crizotinib.

To elucidate the specific cell death pathways induced by Crizotinib in hepatocytes, Crizotinib was combined with various cell death inhibitors in AML12 cells. MTT assay showed that deferasirox DFO combined treatment improved cell viability the most significantly (Fig. 3A), which was validated in HL7702 cells (Fig. 3B), followed by the ferroptosis inhibitor Fer1, apoptosis inhibitor Z-VAD-FMK, necrosis inhibitor Nec1 and pyroptosis inhibitor VX765, while autophagy inhibitors 3-MA and CQ as well as PARP inhibitor Olaparib showed no alleviation (Fig. S5A–G). In addition, Fig. 3C–E revealed that Fer1 and DFO showed a significant reduction of Crizotinib-induced liver injury, as evidenced by decreased ALT, AST levels, and ameliorated liver pathological changes in C57 mice. These results indicate that ferroptosis plays a crucial role in Crizotinib-triggered cell death pathways in hepatocytes.

Subsequently, the alleviating effects of ferroptosis inhibitors on Crizotinib-induced ferroptosis were examined in vitro. Eliminating excess iron ions is a direct way to inhibit ferroptosis. In AML12 cells, DFO effectively reduced the Crizotinib-induced high levels of ACLS4 protein and PTGS2 mRNA, while increasing the low levels of SLC7A11 and GPX4 proteins (Fig. S6A, B). Additionally, DFO decreased iron accumulation, MDA, and lipid ROS levels (Fig. S6C–E). Similar results were observed in HL7702 cells in a concentration-dependent manner (Fig. S7A–C), providing compelling evidence for the role of DFO in mitigating Crizotinib-induced liver ferroptosis. Moreover, the inhibition of lipid peroxidation and the enhancement of antioxidant capacity have been shown to effectively suppress ferroptosis. Fer1 was demonstrated to mitigate Crizotinib-induced ferroptosis, thereby offering protection against Crizotinib-induced liver injury (Fig. S8A–C). Free Fe^2+^ in cells causes lipid peroxidation via the Fenton reaction, the classic ferroptosis pathway. Utilizing FerroOrange dye, an accumulation of Fe^2+^ was measured after Crizotinib treatment, while treatment with DFO reversed this (Fig. S9).

The therapeutic effects of Fer1 and DFO on the three major characteristics of Crizotinib-induced ferroptosis were systemically examined in vivo. Fer1 and DFO increased the protein and mRNA levels of SLC7A11 and GPX4 (Fig. 3F, G), t-GSH levels, and reduced GSH/GSSG ratio (Fig. 3H) to address glutathione deficiency induced by Crizotinib. ICP–MS accurately identified changes in iron levels, showing that Fer1 and DFO decreased iron accumulation, consistent with kit detection results in resolving Crizotinib-induced iron metabolic disorders (Fig. 3I). Additionally, Fer1 and DFO lowered MDA and LPO levels (Fig. 3J), as well as inhibited 4-HNE which is another natural product of lipid peroxidation reaction (Fig. 3K, L), to alleviate Crizotinib-induced lipid peroxidation. The above results indicate that Fer1 and DFO significantly alleviate Crizotinib-induced ferroptosis and hepatotoxicity.

### Nrf2-dependent ferroptosis is involved in Crizotinib-induced hepatotoxicity

After removing the Metabolism and Human Diseases categories, re-analysis of the remaining KEGG pathways showed DEG enrichment in the P53 pathway, which regulates key ferroptosis genes SLC7A11, PTGS2, and GLS2, implicating P53 as a core ferroptosis regulator [33, 34]. However, experiments revealed no significant P53 protein changes after Crizotinib treatment in normal hepatocytes and liver tissues (Fig. S10B–D), suggesting P53-independent mechanisms mediate Crizotinib-induced ferroptosis.

**Fig. 4:**
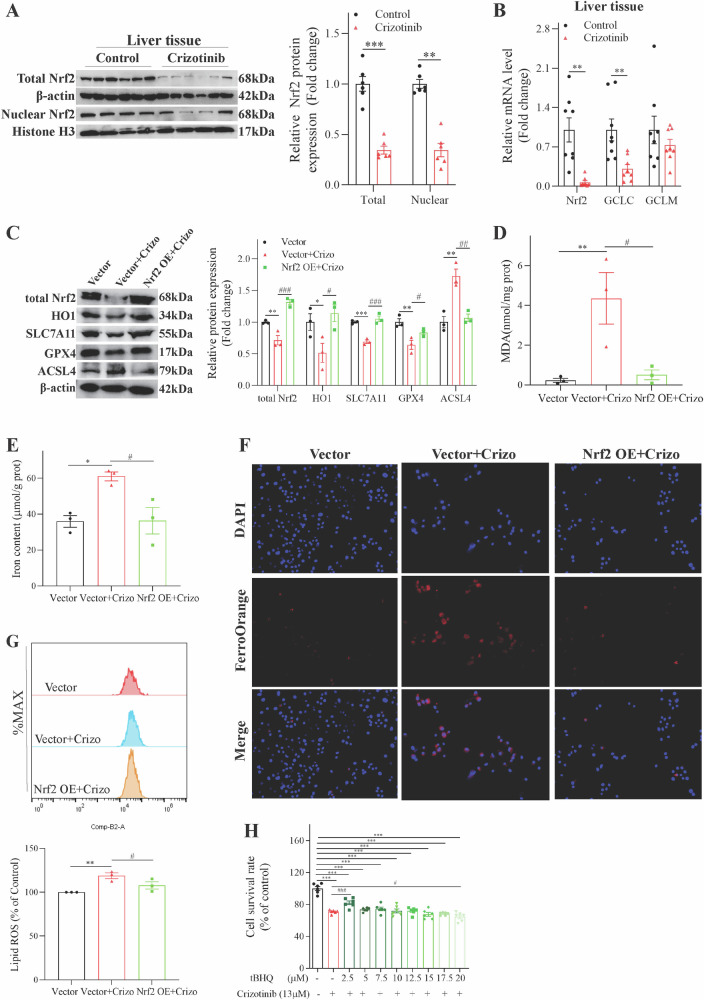
Nrf2 overexpression attenuated Crizotinib-induced hepatocellular ferroptosis in AML12 cells. **A** The protein level of total Nrf2 and nuclear Nrf2 in liver tissues was detected by western blot (*n* = 6). **B** The mRNA levels of Nrf2 and its downstream gene in liver tissues (*n* = 8) were determined by RT-qPCR. Vector or Nrf2 OE transfected AML12 cells were treated with or without 13 μM Crizotinib for 48 h. **C** Protein expression of Nrf2, HO1, SLC7A11, GPX4 and ACSL4 was measured by western blot (*n* = 3). **D** Fold change in MDA level (*n* = 3). **E** Fold change in Iron content (*n* = 3). **F** Fe^2+^ was detected by FerroOrange probe (×20). **G** The induction of lipid ROS was determined by BODIPY™ 581/591 C11 and flow cytometry (*n* = 3). **H** The survival rate of AML12 cells was treated with 13 μM Crizotinib and/or tBHQ at different concentrations for 48 h (*n* = 6). *P < 0.05, **P < 0.01 and ***P < 0.001 vs. control group. ^#^*P* < 0.05, ^##^*P* < 0.01 and ^###^*P* < 0.001 vs. Crizotinib group^.^ Crizo Crizotinib.

In addition to ferroptosis pathways, Reactome enrichment revealed DEGs were enriched in the Nrf2 pathway (Fig. S11A). Mechanistic experiments showed Crizotinib reduced Nrf2 protein and downstream antioxidant target HO-1 levels in normal hepatocytes (Fig. S11B, C) and liver tissues (Fig. 4A) by suppressing Nrf2 mRNA expression (Figs. S11D and 4B). The Crizotinib-mediated inhibition of Nrf2 also reduced mRNA levels of several key Nrf2-regulated ferroptosis genes including SLC7A11, GPX4, NQO1, GCLC, and GCLM (Figs. S11D; 4B), preliminarily suggesting Nrf2 signaling may play a role in Crizotinib-induced ferroptosis.

Follow-up investigations directly examined the regulatory function of Nrf2 in Crizotinib-triggered ferroptosis in AML12 cells. Nrf2 overexpression reversed Crizotinib’s suppression of Nrf2 and HO1 protein, attenuating ferroptosis by restoring SLC7A11 and GPX4 expression (Fig. 4C). Additionally, Nrf2 overexpression mitigated Crizotinib-induced upregulation of ACSL4 (Fig. 4C) and MDA accumulation (Fig. 4D), while reducing total iron content (Fig. 4E), red Fe^2+^ fluorescence (Fig. 4F) and lipid ROS generation (Fig. 4G). Similarly, tBHQ, a potent antioxidant that specifically activates Nrf2 expression, protected against Crizotinib survival rate reduction (Fig. 4H) and indicators of ferroptosis (Fig. S12A–C). Taken together, these comprehensive results demonstrate that Nrf2 activation by overexpression or pharmacological induction significantly alleviates Crizotinib-triggered ferroptosis in hepatocytes.

### Nrf2-overexpression attenuates hepatotoxicity by suppressing ferroptosis in vivo

**Fig. 5:**
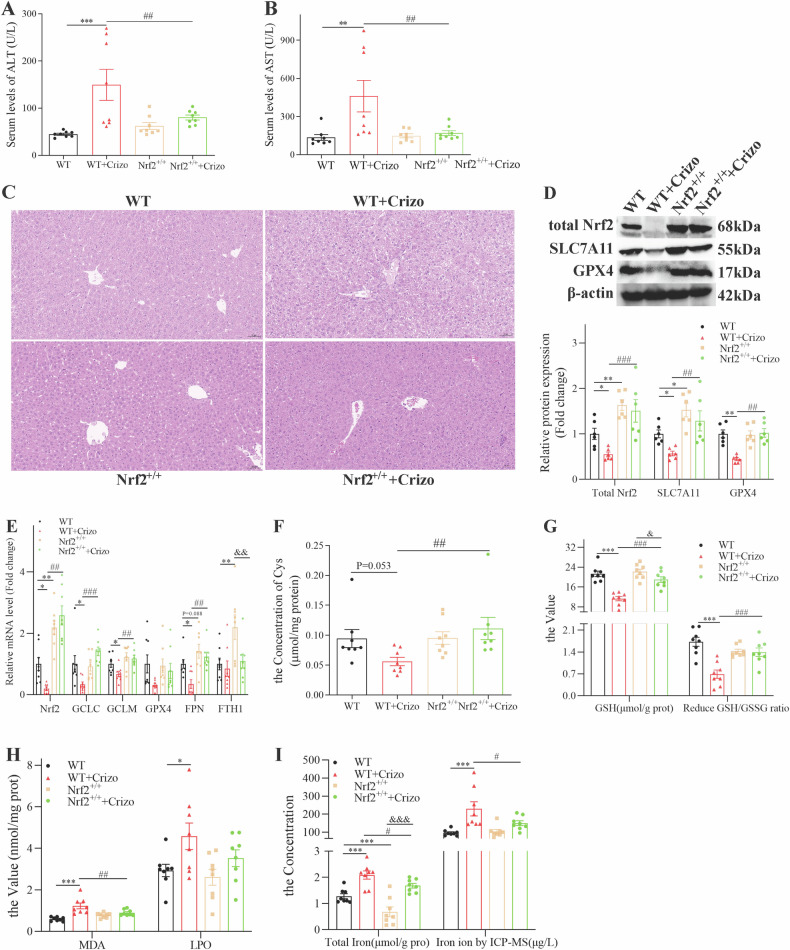
Overexpression of Nrf2-attenuated Crizotinib-induced hepatocytes ferroptosis and hepatotoxicity in vivo. AAV8-TBG- Vector or AAV8-TBG- Nrf2 were injected into C57 male mice through the tail vein. 4 weeks later, mice were treated with 0.5% CMC-Na or 120 mg/kg/day Crizotinib (*n* = 8). **A** and **B** The levels of serum ALT (**A**) and AST (**B**) were measured (*n* = 8). **C** Representative images of H&E staining in liver tissues (20×). **D** The protein levels of total Nrf2, SLC7A11, and GPX4 of liver lysate were analyzed by western blot (*n* = 6). **E** The mRNA levels of Nrf2, GCLC, GCLM, GPX4, FPN, and FTH1 of liver lysate were analyzed by RT-qPCR (*n* = 8). **F**–**H** Cys (**F**, n = 8), GSH, the reduced GSH/HSSG (**G**, *n* = 8), MDA, and LPO (**H**, *n* = 8) were measured by the reagent kit. **I** total Iron concentration was measured by reagent box and ICP–MS (*n* = 8). *P < 0.05, **P < 0.01 and ***P < 0.001 vs. the WT group. ^#^*P* < 0.05, ^##^*P* < 0.01 and ^###^*P* < 0.001 vs. WT+ Crizotinib group. ^&^*P* < 0.05, ^&&^*P* < 0.01 and ^&&&^*P* < 0.001 vs. Nrf2^+/+^ group. Crizo Crizotinib.

Based on our previous findings, we speculated that recovering Nrf2 could rescue Crizotinib-induced hepatotoxicity. Therefore, we overexpressed Nrf2 in mice by injecting AAV8 carrying the TBG promoter with Nrf2. After 4 weeks, mice were treated with vehicle or 120 mg/kg/day Crizotinib for 3 weeks. Remarkably, Nrf2 overexpression partially recovered the increased ALT and AST levels caused by Crizotinib (Fig. 5A, B). H&E staining showed Nrf2-overexpressing mice had improved histology with fewer vacuoles after Crizotinib treatment (Fig. 5C), suggesting that the overexpression of Nrf2 reduced the induction effect of Crizotinib. Then Nrf2 protein and mRNA were recovered in Crizotinib-treated mice with Nrf2 overexpression (Fig. 5D, E).

Furthermore, we detected the indicators of glutathione metabolism in those mouse models. Compared with vehicle mice after Crizotinib treatment, Nrf2-overexpressing mice increased the protein and mRNA level of SLC7A11 and GPX4 (Fig. 5D, E), and the mRNA levels of glutamate- cysteine ligase catalyzes/modify subunits GCLC and GCLM (Fig. 5E). Similarly, Nrf2 upregulation increase the cys (Fig. 5F), t-GSH and the ratio of reduced GSH/ GSSG (Fig. 5G). Next, we found that lipid peroxidation products MDA and LPO were alleviated in Crizotinib-treated mice by overexpressing Nrf2 (Fig. 5H). Concurrently, Nrf2 overexpression upregulated the sole known iron efflux transporter FPN without affecting the iron storage protein FTH1 (Fig. 5E). And Nrf2 overexpression decreased total iron content by kit assay and ICP-MS quantification (Fig. 5I), indicating alleviation of Crizotinib-induced iron imbalance. Collectively, these results demonstrated that Crizotinib caused liver ferroptosis by inhibiting Nrf2, a potential therapeutic target for its hepatotoxicity.

### Knockdown or activation inhibition of Stat1 alleviates Crizotinib-induced ferroptosis

mRNA sequencing revealed the Stat family as a top differentially expressed transcription factor (TF), regulating cell cycle, growth, and survival (Fig. S13A). Western blots demonstrated Crizotinib-induced Stat1 phosphorylation at Tyr701 and Ser727 in AML12 cells (Fig. S13B) and liver tissue (Fig. S13C), suggesting a link between Stat1 activation and hepatoxicity.

**Fig. 6:**
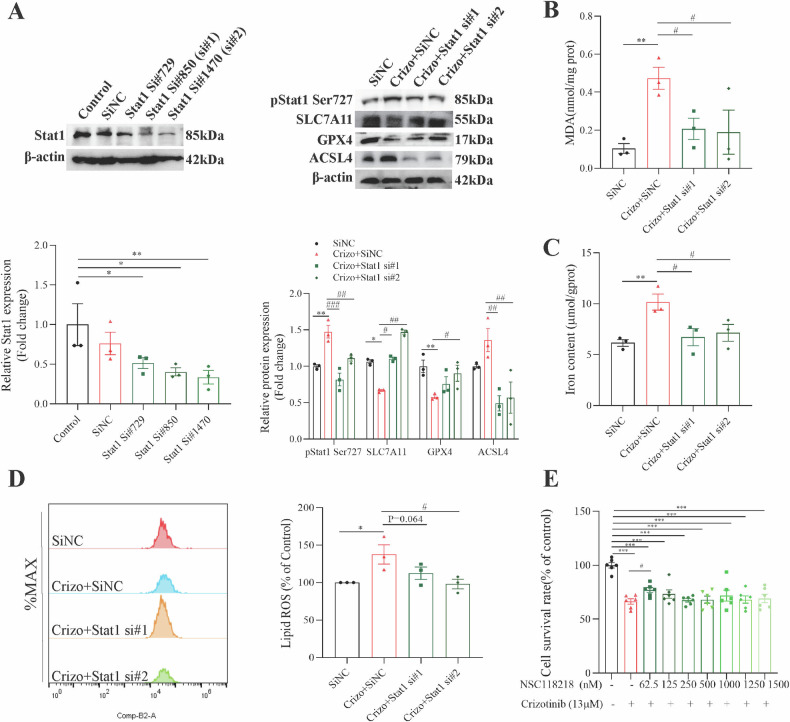
Silence of Stat1 attenuated Crizotinib-induced hepatocellular ferroptosis in AML12 cells. SiNC or Stat1 siRNA transfected AML12 cells were treated with or without 13 μM Crizotinib for 48 h. **A** Protein expression of Stat1, pStat1 Ser727, SLC7A11, GPX4 and ACSL4 was measured by western blot (*n* = 3). **B** Fold change in MDA level (*n* = 3). **C** Fold change in Iron content (*n* = 3). **D** The induction of lipid ROS was determined by BODIPY™ 581/591 C11 and flow cytometry (*n* = 3). **E** The survival rate of AML12 cells was treated with 13 μM Crizotinib and/or NSC118218 at different concentrations for 48 h (*n* = 6). *P < 0.05, **P < 0.01 and ***P < 0.001 vs. control group. ^#^*P* < 0.05, ^##^*P* < 0.01 and ^###^*P* < 0.001 vs. Crizotinib group. Crizo Crizotinib.

Then, Stat1 knockdown alleviated pStat1 Ser727 expression and promoted SLC7A11 and GPX4 expression in Crizotinib-treated AML12 cells, enhancing ferroptosis defense (Fig. 6A). Stat1 silencing also decreased Crizotinib-induced high ACSL4 expression (Fig. 6A), MDA accumulation (Fig. 6B), total iron content (Fig. 6C) and lipid ROS accumulation (Fig. 6D), reducing lipid peroxidation. Then, we selected NSC118218, a Stat1 activation inhibitor that specifically decreases Stat1, to evaluate its potential for treating Crizotinib-induced hepatotoxicity. Similarly, NSC118218 effectively protected against Crizotinib-induced survival rate reduction (Fig. 6E) and ferroptosis (Fig. S14A–C). These results demonstrated that Stat1 inhibition significantly mitigated Crizotinib-induced hepatocellular ferroptosis.

### The inhibition of Stat1 is related to Nrf2 upregulation

**Fig. 7:**
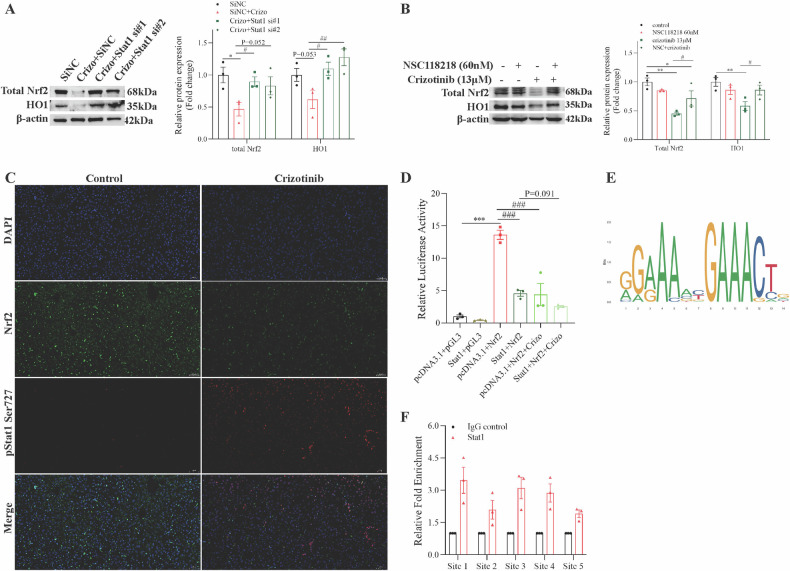
STAT1 is a transcription factor of the Nrf2 gene. SiNC or Stat1 siRNA transfected AML12 cells were treated with or without 13 μM Crizotinib for 48 h. **A** protein expression of total Nrf2 and HO1 was measured by western blot (*n* = 3). AML12 cells were treated with 13 μM Crizotinib and/or NSC118218 at different concentrations for 48 h. **B** protein expression of total Nrf2 and HO1 was measured by western blot (*n* = 3). **C** Representative images of immunofluorescence for p-Stat1 (Ser727) staining and Nrf2 staining in liver tissues (×20). **D** Dual-luciferase reporter assay of Nrf2 and STAT1 with or without Crizotinib treatment (*n* = 3). **E** Stat1 binding motif provided by the JASPAR database. **F** STAT1 binds to its binding sites in the Nrf2 promoter region as shown by the ChIP assay in AML12 cells with or without the transfection of Stat1 overexpression plasmid. *P < 0.05, **P < 0.01 and ***P < 0.001 vs. control group. ^#^*P* < 0.05, ^##^*P* < 0.01 and ^###^*P* < 0.001 vs. Crizotinib group.

mRNA sequencing suggested Nrf2 may be a Stat1 target gene (Fig. S15). Experiments showed Stat1 silencing increased Nrf2 and HO1 protein compared to Crizotinib treatment (Fig. 7A), corroborated by the Stat1 inhibitor NSC118218 (Fig. 7B). Immunofluorescence revealed Nrf2 was present in the cytoplasm and minimally in nuclei in a physiological manner. Crizotinib markedly reduced Nrf2, especially in nuclei, suggesting inhibited nuclear translocation. Meanwhile, pStat1 Ser727 was low in controls but increased in nuclei after Crizotinib treatment (Fig. 7C). Together, these results indicate Crizotinib induces ferroptosis by promoting Stat1 phosphorylation and nuclear translocation, consequently suppressing Nrf2 expression and nuclear localization.

To further elucidate the transcriptional regulation between Stat1 and Nrf2, luciferase assays were performed in AML12 cells. Compared to the pcDNA3.1+Nrf2 group, luciferase activity was significantly decreased in Stat1+Nrf2 group, indicating Stat1 negatively regulates Nrf2 transcription (Fig. 7D). Crizotinib further reduced luciferase activity in pcDNA3.1+Nrf2 group and enhanced inhibition in Stat1+Nrf2 cells (Fig. 7D), confirming Crizotinib exacerbates Stat1-mediated Nrf2 suppression. Subsequently, the online bioinformatics JASPAR database (http://jaspar.generge.net) was also used, and the results also indicated that there was a potential binding site between STAT1 and the Nrf2 promoter region (Table S6). Since there were multiple predicted binding sites, some in close proximity, a total of five ChIP primers were designed (Table S5). ChIP-qPCR validated significant Stat1 enrichment at sites 1, 3, and 4 in the Nrf2 promoter (Figs. S16; 7E, F). Together, these comprehensive results demonstrate that Nrf2 is a direct transcriptional target suppressed by Stat1, which is further enhanced by Crizotinib exposure.

### The inhibition of phosphorylation of Stat1 Ser727 is related to transcriptional Nrf2 inhibition

Crizotinib-induced Stat1 phosphorylation at Ser727 and Tyr701 sites, but their individual roles in regulating ferroptosis were unclear. To investigate Tyr701, we constructed Stat1 Tyr701A and Tyr701F plasmids to mimic Tyr701 dephosphorylation (Fig. S17A and C). Compared to Crizotinib alone, Tyr701 dephosphorylation did not affect ferroptosis indicators, including the protein expression of SLC7A11, GPX4, or iron levels (Fig. S17A-D). After 48 h of Crizotinib treatment in AML12 cells, Tyr701 dephosphorylation also did not relieve Crizotinib-induced decreases in SLC7A11 and GPX4 (Fig. S17E) or increases in total iron (Fig. S17F). These results suggest limited involvement of Tyr701 in regulating Crizotinib-induced liver ferroptosis.

**Fig. 8:**
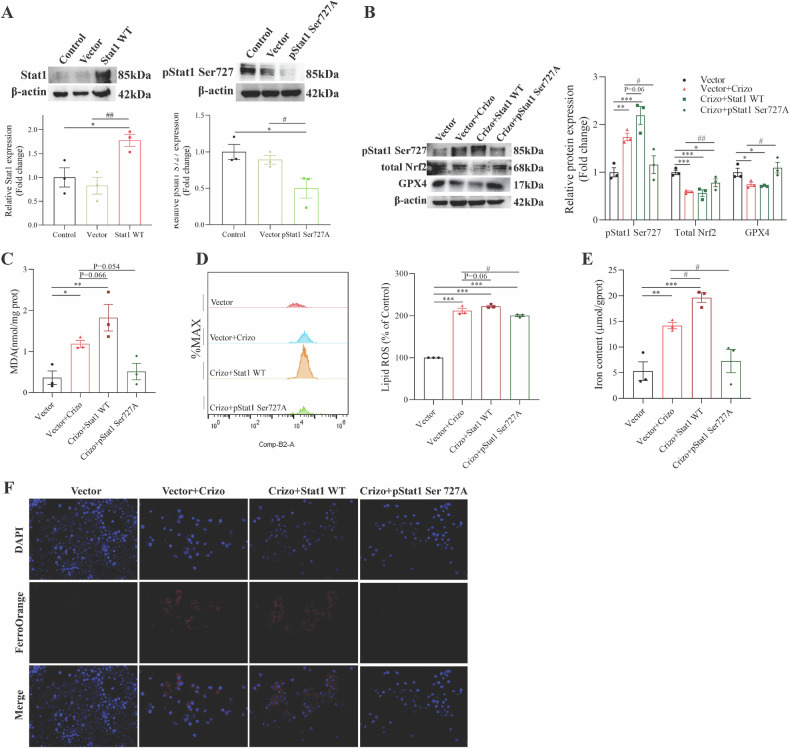
Phosphorylation of Stat1 Ser727 induction is associated with Nrf2 inhibition and ferroptosis (*n* = 3). **A** Transfection efficiency of Stat1 WT and pStat1 Ser727A plasmid was evaluated by western blot. **B** Protein expression of pStat1 Ser727, total Nrf2, and GPX4 was measured by western blot. **C** Fold change in MDA level. **D** The induction of lipid ROS was determined by BODIPY™ 581/591 C11 and flow cytometry. **E** Fold change in Iron content. **F** Fe^2+^ was detected by FerroOrange probe (×20). *P < 0.05, **P < 0.01 and ***P < 0.001 vs. control group. ^#^*P* < 0.05 and ^##^*P* < 0.01 vs. Crizotinib group.

Subsequently, the role of Ser727 phosphorylation was investigated. As shown in Fig. 8A, both the overexpressed Stat1 WT plasmid mimicking phosphorylation and the Stat1 Ser727A plasmid mimicking dephosphorylation were successfully constructed. Compared to Crizotinib alone, Ser727 dephosphorylation significantly increased the protein expression of Nrf2 and GPX4 (Fig. 8B), and decreased MDA (Fig. 8C) and lipid ROS levels (Fig. 8D). Additionally, Ser727 dephosphorylation significantly reduced the high total iron levels (Fig. 8E) and Fe^2+^ levels (Fig. 8F) induced by Crizotinib. These comprehensive results demonstrate the Stat1 Ser727 site specifically participates in regulating Crizotinib-induced ferroptosis in hepatocytes by suppressing Nrf2 expression and activity.

### pharmacological interventions potentially linked to the Stat1/Nrf2 pathway rescued Crizotinib-induced hepatic ferroptosis

**Fig. 9:**
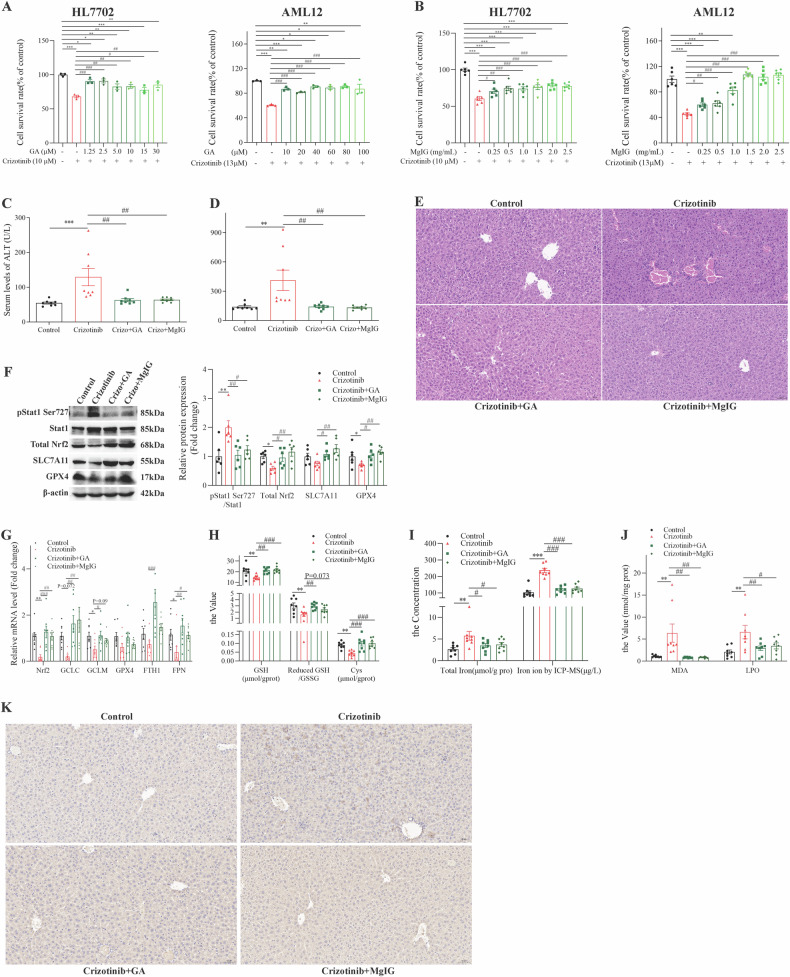
GA and MgIG attenuate Crizotinib-induced hepatocyte ferroptosis via the Stat1/Nrf2 pathway in vivo. **A** and **B** the cell viability of HL7702 and AML12 cells treated with 13 μM Crizotinib and/or GA (*n* = 3) or MgIG (*n* = 6) at different concentrations for 48 h. C57 male mice were treated with 120 mg/kg/day Crizotinib and/or 5 mg/kg/day GA or 10 mg/kg/day MgIG for 3 weeks. **C** and **D** The levels of serum ALT (**C**) and AST (**D**) were analyzed (*n* = 8). **E** Representative images of H&E staining in liver tissues (×20). **F** The protein levels of pStat1 Ser727, Stat1, total Nrf2, SLC7A11, and GPX4 of liver lysate were analyzed by western blot (*n* = 6). **G** The mRNA levels of Nrf2, GCLC, GCLM, GPX4, FPN, and FTH1 of liver lysate were analyzed by RT-qPCR (*n* = 8). **H**, **J** Cys, GSH, the reduced GSH/HSSG (**H**, *n* = 8), MDA, and LPO (**J**, *n* = 8) were measured by reagent kit. **I** Total Iron concentration was measured by reagent box and ICP-MS (*n* = 8). **K** Representative pictures of 4-HNE expression in each group measured by immunohistochemical staining (×20). *P < 0.05, **P < 0.01 and ***P < 0.001 vs. the control group. ^#^*P* < 0.05, ^##^*P* < 0.01 and ^###^*P* < 0.001 vs. Crizotinib group.

Among licorice components, the triterpene GL did not alleviate Crizotinib-induced cell death in HL7702 and AML12 cells (Fig. S18A), while GA was protective (Fig. 9A). The flavonoids ISL and LQ alleviated death in HL7702 cells induced by Crizotinib, but only 2.5 μM ISL alleviated death in AML12 cells (Fig. S18B, C). The licorice preparation MgIG significantly alleviated Crizotinib-induced hepatotoxicity in both cell lines (Fig. 9B). Importantly, GA and MgIG did not affect the anticancer activity of Crizotinib in ALK + NSCLC cell lines H2228 and H3122 (Figs S19A–D), demonstrating selective protection against liver toxicity.

Subsequently, we delved into the mechanisms by which GA and MgIG mitigate hepatotoxicity induced by Crizotinib. Western blot analyses, depicted in Figs. S20A and S21A revealed that both GA and MgIG effectively reduce Crizotinib-induced phosphorylation of Stat1 and counteract the inhibition of the Nrf2 pathway, as well as the suppression of SLC7A11 and GPX4, and the upregulation of ACSL4 in AML12 cells. Additionally, GA and MgIG were shown to diminish the Crizotinib-induced increase in total iron content (Figs. S20B and S21B), MDA levels (Figs. S20C and S21C), and lipid ROS production (Fig. S21D). Importantly, the intensified red fluorescence of Fe^2+^, a consequence of Crizotinib treatment, was significantly reduced by GA and MgIG treatment in AML12 cells (Fig. S21E).

Furthermore, we evaluated the therapeutic potential of GA and MgIG in vivo by treating Crizotinib-administered mice with GA or MgIG. The combination treatments notably lowered serum ALT and AST levels (Fig. 9C, D) and improved liver histology with less inflammatory cell infiltration and bleeding spots (Fig. 9E), indicating reduced hepatotoxicity. These improvements were accompanied by the normalization of Crizotinib-induced aberrations in key proteins and genes such as pStat1, Nrf2, SLC7A11, GPX4, GCLC, and GCLM, alongside enhanced glutathione synthesis and iron homeostasis (Fig. 9F–I). Additionally, GA and MgIG reduced some indices related to lipid peroxidation, including MDA and LPO, and corrected the expression of 4-HNE (Fig. 9J, K). These results highlight the ability of GA and MgIG to mitigate Crizotinib-induced liver damage, potentially via the Stat1/Nrf2 pathway, underscoring their promise as clinical interventions for hepatotoxicity.

## Discussion

**Fig. 10:**
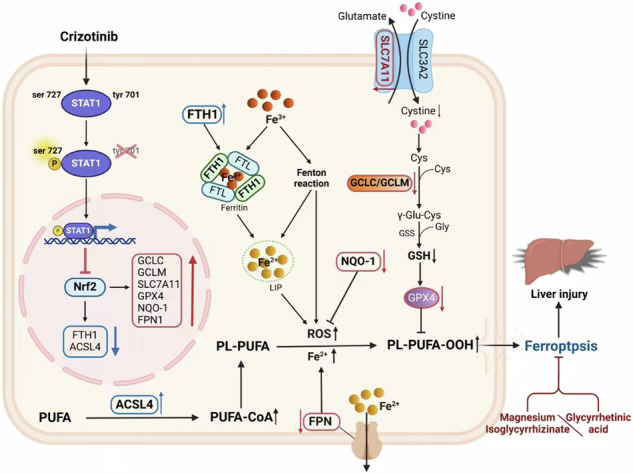
Schematic diagram showing the mechanism of Crizotinib-induced hepatotoxicity. Crizotinib triggers the phosphorylation of Stat1 at Ser727, which facilitates the transcriptional suppression of Nrf2 expression upon nuclear entry, promoting ferroptosis, and leading to liver toxicity.

DILI stands as a prevalent and grave adverse reaction associated with over 1000 medications, leading to the causes of ALF in developed nations and often prompting FDA drug safety warnings [35]. Notably, the incidence of DILI in our country surpasses that in Europe and the United States, marking it as a significant health concern [36]. It is fatal to patients without effective intervention. Currently, the cessation of suspect drugs remains the primary management strategy for DILI, with very few treatment options available [37]. In our research, we uncovered the critical role of ferroptosis in Crizotinib-induced hepatotoxicity and elucidated the molecular interplay between Stat1 and Nrf2. We pinpointed the phosphorylation of Stat1 at Ser727 as essential for Nrf2 transcriptional regulation, impacting liver ferroptosis. Through a series of in vitro and in vivo experiments, we demonstrated that GA and MgIG, potentially by modulating the Stat1/Nrf2 pathway, offer protection against Crizotinib-induced hepatotoxicity and ferroptosis (Fig. 10). These findings suggest the potential of GA and MgIG in enhancing the safety of Crizotinib use in clinical settings, addressing a critical gap in DILI management.

At the molecular level, the toxicity of TKIs to organs can be broadly categorized into on-target and off-target toxicities [38]. This distinction is crucial in understanding the mechanisms behind Crizotinib-induced hepatotoxicity. Despite the liver’s minimal expression of pharmacodynamic targets, ALK and ROS1, as indicated by the UniProt protein database, the risk of Crizotinib-induced hepatotoxicity attributed to these targets appears negligible. This observation led us to explore the role of Met, another target of Crizotinib, in hepatotoxicity. Our findings show a decrease in pMet relative to total Met in AML12 cells following Crizotinib exposure (Fig. S22A). However, the use of SCC244, a Met-specific inhibitor, did not mitigate the Crizotinib-induced reduction in cell survival (Fig. S22B), nor did silencing of Met impact the expression of SLC7A11, GPX4, or iron content, with or without Crizotinib treatment (Fig. S22C–F). These results indicate that Crizotinib-induced ferroptosis is independent of its “on-target” effects on ALK, ROS1, and Met. On the other hand, off-target effects have been recognized as a primary source of drug toxicity. Given Crizotinib’s nature as a multi-target TKI, this increases its propensity for off-target toxicity. Identifying specific targets involved in drug-induced toxicity remains a pivotal step in predicting and mitigating potential adverse effects. However, there are currently no reports identifying the key targets responsible for Crizotinib-induced hepatotoxicity, highlighting a significant gap in our understanding and intervention strategies for this.

In our prior work, we observed both apoptosis and necrosis in Crizotinib-induced hepatotoxicity [21], with literature also pointing to necroptosis without necrosis [39]. This discrepancy led us to further investigate through mRNA sequencing, revealing significant enrichment of DEGs in ferroptosis and related pathways. By using cell death inhibitors, we found that DFO significantly improved Crizotinib-induced low cell viability in AML12 cells. Animal studies corroborated the effectiveness of DFO in reducing elevated ALT and AST levels and mitigating hepatic damage, highlighting ferroptosis as a potential primary mechanism of Crizotinib-induced cell death. Ferroptosis is an iron-dependent form of cell death, regulated through complex biochemical pathways and distinct from other cell death mechanisms like apoptosis and necrosis, both in its triggers—iron accumulation and lipid peroxidation—and outcomes [40, 41]. This process begins when the cellular reduction system is inactivated, leading to the reduction of trivalent to divalent iron ions, further catalyzing ROS production through the Fenton reaction. This oxidative stress causes extensive damage to cell membranes by peroxidizing polyunsaturated fatty acids, leading to cell rupture [42]. Ferroptosis involves both extrinsic (e.g., decreased cysteine or glutamine uptake and increased iron uptake) and intrinsic pathways (e.g., GPX4 inhibition) [43], making its detection challenging due to the absence of discriminating markers unique to this form of cell death. Iron and lipid peroxidation must be observed, and a combination of markers evaluated to exclude other forms of cellular stress [44]. Our research into Crizotinib-induced hepatotoxicity examined various indicators to underscore ferroptosis’s pivotal role, highlighting the necessity of a nuanced approach in its identification and the implications of iron metabolism in DILI.

As our understanding of ferroptosis deepens, the critical role of Nrf2 in this process becomes clearer, particularly how it mediates the cell’s antioxidant capacity, iron homeostasis, and metabolic status through its target genes [45]. Nrf2, which regulates key ferroptosis inhibitors xCT and GPX4, is usually kept at low levels through Keap1-mediated degradation [46, 47]. Ferroptotic stress activates Nrf2, prompting it to activate numerous protective genes involved in iron metabolism and oxidative defense [48, 49]. However, increased lipid oxidation and downstream Nrf2 target inactivation significantly enhance the overall protein–lipid oxidation and ferroptosis in disease environments with low Nrf2, further promoting disease progression [50, 51]. In our study, Reactome found that “Nuclear events mediated by Nrf2” and “Keap1-Nrf2 pathway” were among the top 20 DEGs enrichment sites. And Nrf2 overexpression was found to significantly improve Crizotinib-induced ferroptosis and liver injury, highlight targeting Nrf2 as a promising strategy in conditions characterized by lipid peroxidation and ferroptosis.

Stat1 exhibits dual roles in liver cancer, activating tumor suppression pathways while also facilitating tumor angiogenesis, immune evasion, and drug resistance [52]. However, Stat1 activation is increasingly linked to liver inflammation exacerbation and fibrosis progression [53, 54]. Beyond inflammatory mediation, Stat1 upregulates genes for fat and triglyceride synthesis, promoting hepatic lipid peroxidation and cell death [54]. Hepatotoxic drugs like aminothiazole and aspirin activate Stat1, enhancing oxidative stress and mitochondrial apoptotic pathways and leading to hepatocyte apoptosis [55]. The regulatory role of Stat1 in liver disease, affecting inflammation, metabolism, apoptosis, and proliferation, necessitates further investigation. A retrospective pharmacogenomic study identified the Stat1 SNP rs10208033’s association with Crizotinib-induced hepatotoxicity in Chinese patients with NSCLC treated with Crizotinib, highlighting the need for mechanistic exploration [56]. Our study shows that Stat1 knockdown ameliorates Crizotinib-induced ferric overload and lipid peroxidation in hepatocytes, reducing iron death. Mechanistically, we present the first direct evidence of a transcriptional negative regulatory relationship between Stat1 and Nrf2, with Crizotinib promoting this transcriptional activity, suggesting that inhibiting Stat1 could offer an intriguing intervention strategy.

The phosphorylation of STAT1 is not only pivotal for its activation but also serves as a critical mechanism for the nuanced regulation of cellular signaling. Unphosphorylated or partially phosphorylated STAT1 fails to elicit a complete downstream immune response, with Tyr701 phosphorylation being essential for a comprehensive immune response mediated by IFN-γ [57, 58]. Studies elucidate the significant roles of Tyr701 and Ser727 phosphorylation sites in cellular processes like cell death and cell cycle regulation, with the importance of each site varying depending on the cellular context and specific signaling events. Tyr701 phosphorylation is intimately linked to STAT1’s activation and its pro-apoptotic functions, enhanced by agents like doxorubicin, illustrating its central role in STAT1-mediated apoptotic signaling [59]. Conversely, Ser727 phosphorylation is key in cell cycle regulation, particularly in G0/G1 arrest, mediated by changes in c-Myc, cyclins, and p27 levels, indirectly influencing apoptosis through growth arrest [60]. This intricate interplay underscores the context-dependent significance of each phosphorylation site in determining cell fate, a nuance further explored in our study where Crizotinib-induced STAT1 phosphorylation at both sites in hepatocytes. Crizotinib induced Stat1 at both sites in hepatocytes. However, p-Stat1 Tyr701 was not involved in Nrf2-induced ferroptosis or Crizotinib-induced hepatotoxicity. The other functions of p-Stat1 Tyr701 under Crizotinib treatment warrant further investigation

Licorice, a cornerstone of traditional Chinese medicine, is celebrated for its hepatoprotective and detoxifying virtues, attributed to its bioactive constituents such as triterpenes (GL and GA), and flavonoids (ISL and LQ) [61, 62]. GA, derived from licorice root, emerges as a pivotal compound, underpinning the herb’s biological activities, including anti-inflammatory, immunomodulatory, antitumor, and antioxidative properties [63**–**65]. MgIG, a refined licorice derivative, stands out for its clinical application in liver ailments, leveraging its targeted hepatoprotection, antioxidation, and anti-inflammatory capabilities [66**–**68]. Despite the acknowledged therapeutic potential of GA and MgIG in DILI, their efficacy against Crizotinib-induced hepatotoxicity and the underlying mechanisms remained unexplored until our investigation revealed their capacity to inhibit Stat1 phosphorylation at Ser727 and restore Nrf2 levels, thus preventing ferroptosis induced by Crizotinib. This breakthrough not only illuminates the regulatory interplay between Stat1 and Nrf2 but also positions GA and MgIG as promising agents against Crizotinib hepatotoxicity. Exploring further, we assessed the impact of ISL, a licorice-derived flavonoid known for its hepatoprotective and cardioprotective properties [69], on Crizotinib-induced hepatotoxicity. Preliminary results demonstrated the potential of ISL to rejuvenate the Nrf2 pathway, bolster antioxidant defenses, and mitigate liver ferroptosis in HL7702 cells post-Crizotinib exposure (Fig. S23). This indicates that ISL may also mitigate Crizotinib-induced hepatotoxicity. However, at the same time, there are still some questions that need to be explored further. Firstly, based on the current study, our findings only demonstrate that GA and MgIG significantly alleviate Crizotinib-induced hepatotoxicity and ferroptosis at both cellular and animal levels, with a mechanism potentially related to the Stat1/Nrf2 pathway. To conclusively prove that the broad hepatoprotective effects of GA and MgIG alleviate liver damage primarily through the inhibition of ferroptosis rather than other mechanisms, further in-depth research is necessary. Secondly, further research is warranted to understand how GA and MgIG impact the balance between the therapeutic efficacy and liver toxicity over extended clinical use of Crizotinib. In addition, in light of the demonstrated therapeutic impacts of GA, ISL, and MgIG on Crizotinib-triggered liver ferroptosis, it prompts a pivotal inquiry: might these compounds harbor the potential for broader applications in the realm of hepatic ailments tied to dysregulated iron metabolism? This question extends to high iron metabolic liver diseases with distinct yet overlapping metabolic dysfunctions. The prospect of GA and MgIG offering a novel therapeutic avenue in these contexts underscores the imperative for more expansive and detailed investigations, aiming to unravel the multifaceted roles of iron homeostasis in liver health and disease. Further exploration into this domain could illuminate new pathways for intervention and management of these prevalent and challenging liver diseases.

Our study acknowledges several limitations. Primarily, our in vitro investigations employed the AML12 and HL7702 cell lines, which, despite their widespread use, do not entirely mimic primary hepatocyte behavior. Moreover, while we concentrated on the mechanisms underlying hepatocyte injury-induced parenchymal cell death, histopathological observations of HE-stained liver tissues revealed significant inflammatory infiltration post-Crizotinib treatment, hinting at an unexplored potential macrophage-mediated inflammatory response. Additionally, the in vivo role of Stat1 Ser727 in this context was not exhaustively studied. These gaps underscore the necessity for further research to validate and expand upon our findings.

## Conclusion

The phosphorylation activation of Stat1 Ser727, rather than Tyr701, promotes ferroptosis through transcriptional inhibition of Nrf2 expression and highlights MgIG and GA as potential therapeutic approaches to enhance the safety of Crizotinib- based cancer therapy.

### Supplementary information

Supplementary Figure and Tables

Original Western Blots

## Data Availability

All data generated or analyzed during this study are included in this published article and its Supplementary Information files. The RNAseq data generated in this study have been deposited in NCBI’s Short Read Archive (SRA) with accession number PRJNA1136570. Further information and requests for resources and reagents should be directed to and will be fulfilled by the corresponding authors, YM (yanmiao@csu.edu.cn).
