# Supplementary material for: The involvement of the Stat1/Nrf2 pathway in exacerbating Crizotinib-induced liver injury: implications for ferroptosis
Source: Cell Death Dis. 2024 Aug 19;15(8):600. doi: 10.1038/s41419-024-06993-z (PMC11333746; doi:10.1038/s41419-024-06993-z)
Supplement: Supplementary file 1 — Supplementary Figure and Tables [file 41419_2024_6993_MOESM1_ESM.docx]

**Supplementary Figures**


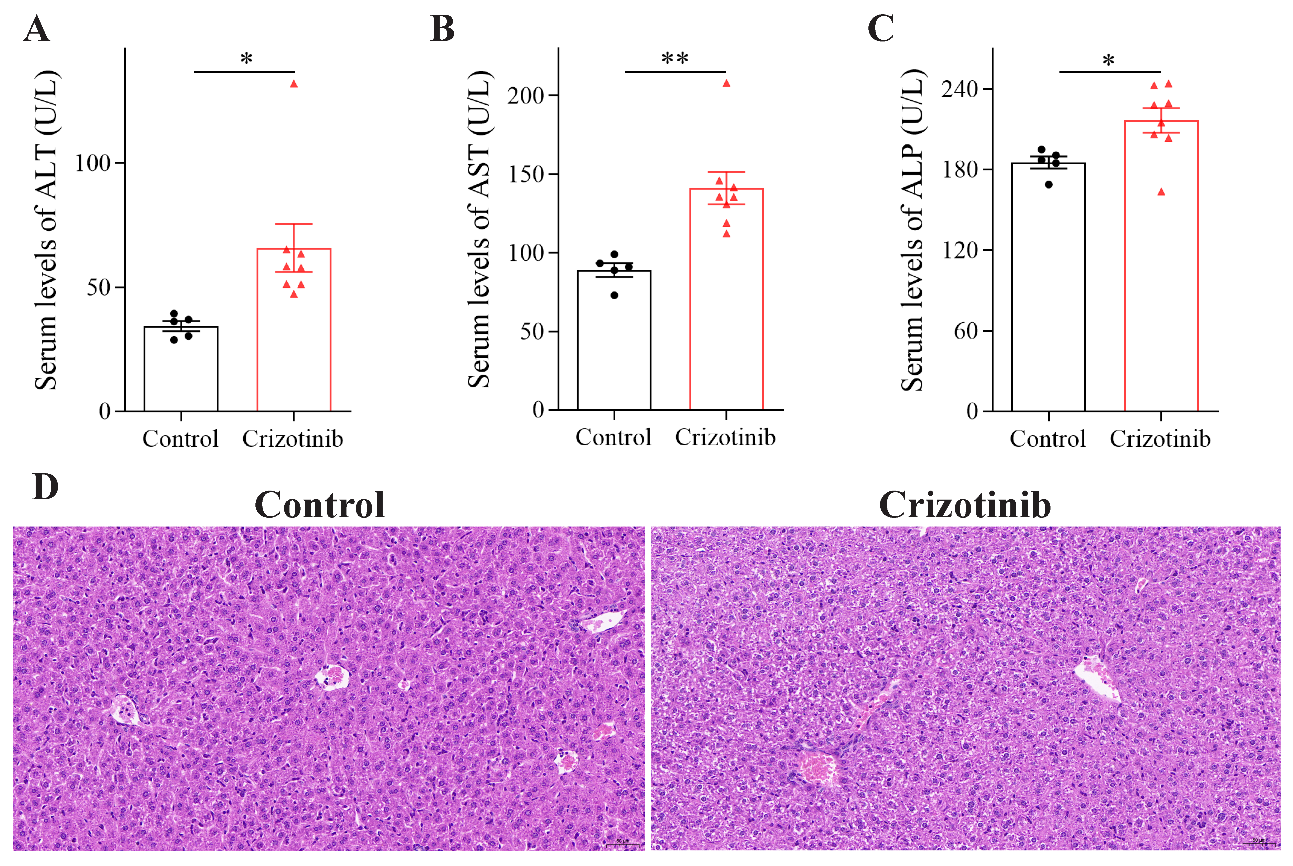


**Supplementary Figure 1. 120 mg/kg/d Crizotinib for 3 weeks caused liver injury *in vivo*. (A-D)** Serum ALT (A), AST (B), ALP (C) levels and liver pathologic changes (D, H&E staining, 20×) of mice were determined to evaluate Crizotinib- caused hepatotoxicity (n=5-8). ^*^*P*<0.05 and ^**^*P*<0.01 vs. Control group.


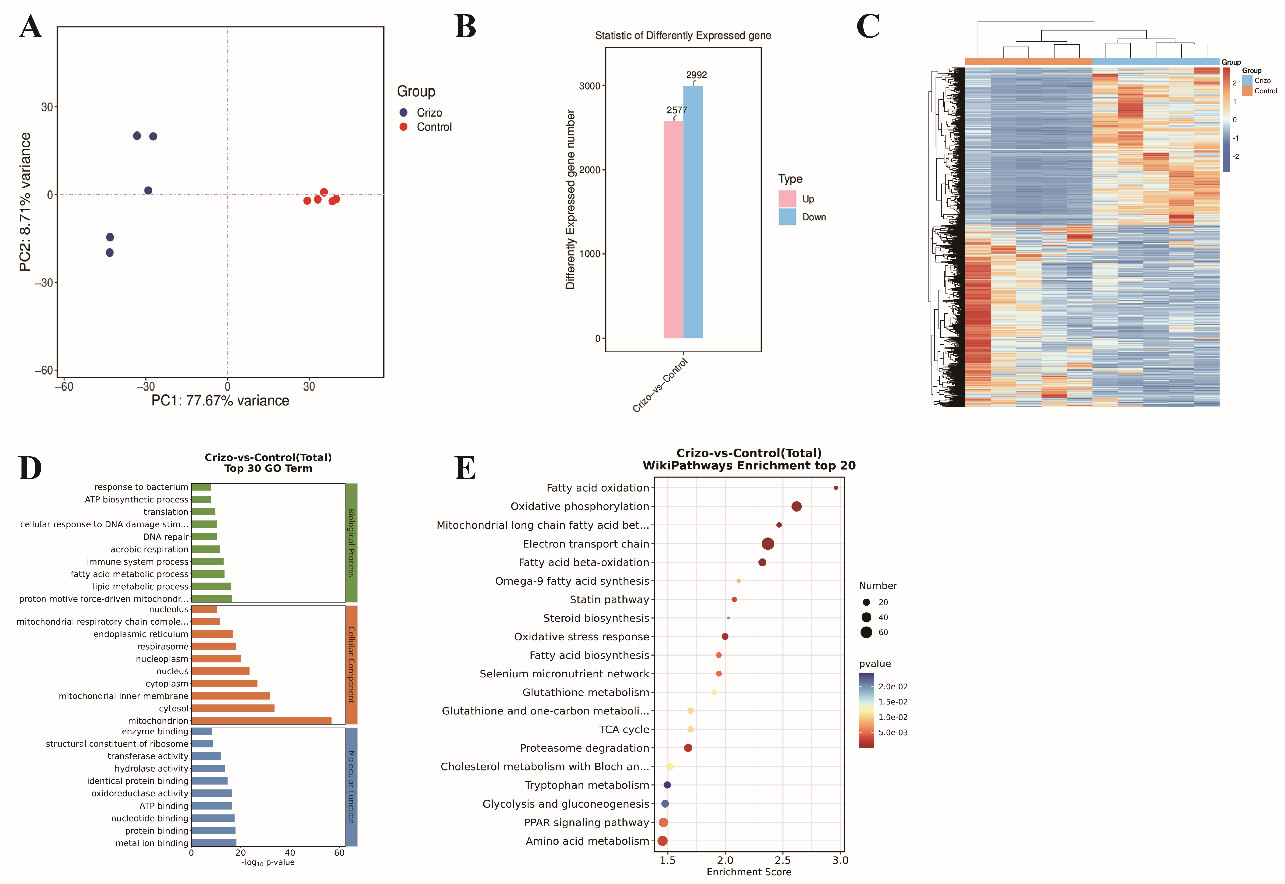


**Supplementary Figure 2. mRNA sequencing data analysis of Crizotinib- induced hepatotoxicity model (n= 5).** **(A)** PCA diagram. **(B-C)** Histogram (B) and heat map (C) of DEGs between the control and Crizotinib group. **(D-E)** Signification enriched top 30 GO terms (D) and top 20 WikiPathways (E) for the differentially expressed mRNA between the control and Crizotinib group. Abbreviation: Crizo, Crizotinib.


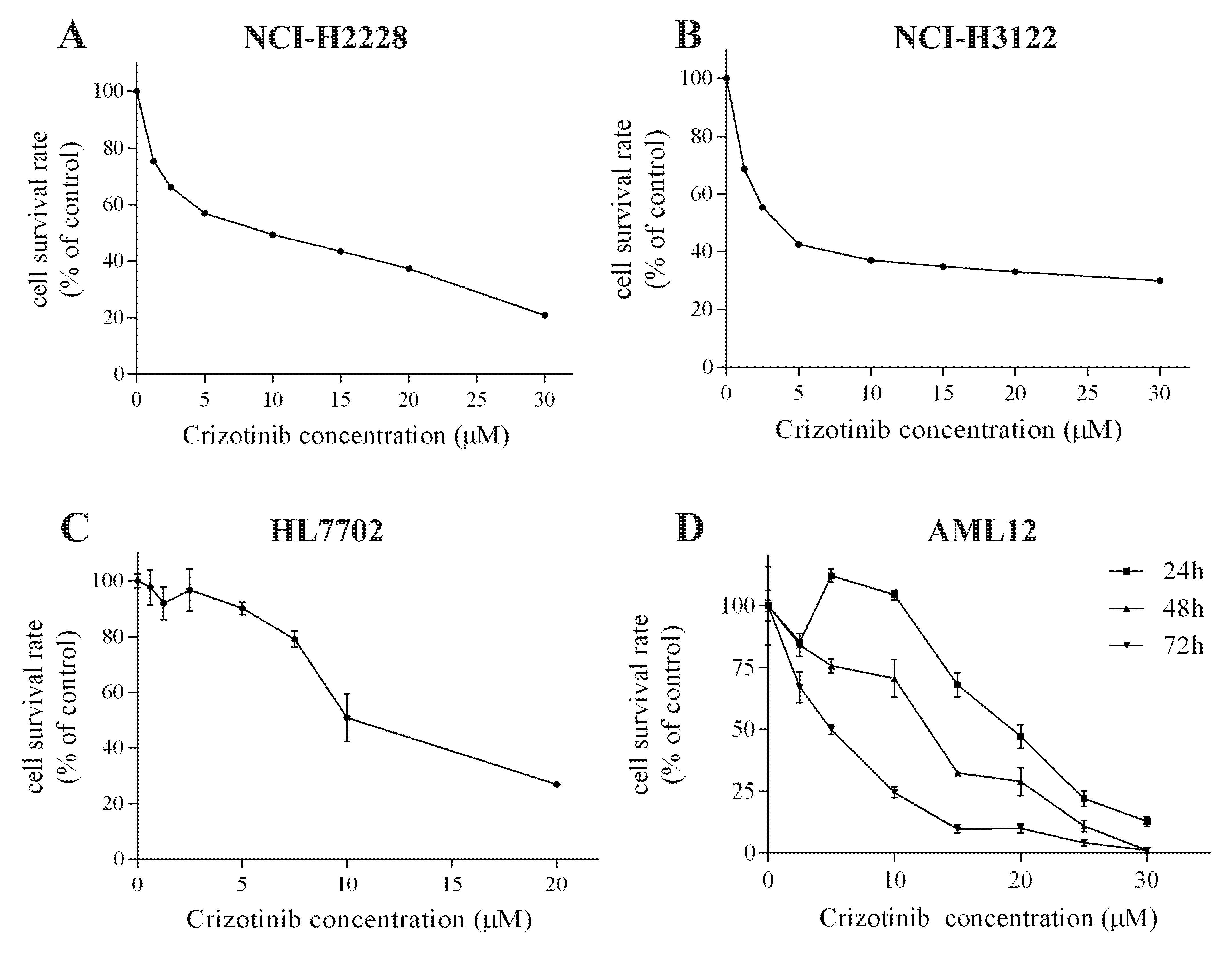


**Supplementary Figure 3. The cell survival rate of NCI-H2228 cells (A), NCI-H3122 cells (B), HL7702 cells (C) and AML12 cells (D) treated with 0-30 μM Crizotinib for 24-72 h was measured by MTT assay** (n=3 or 6)**.**


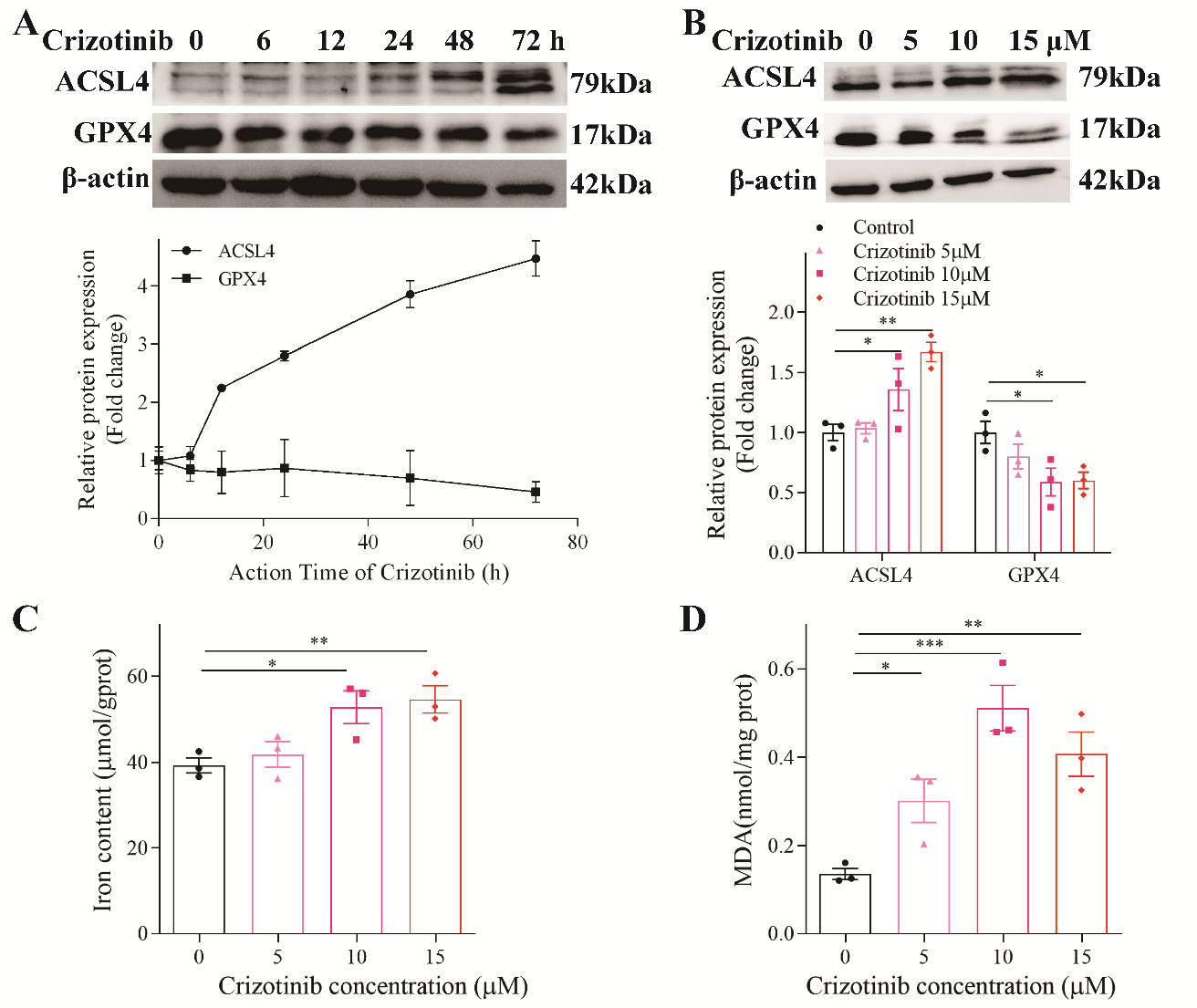


**Supplementary Figure 4. Crizotinib induced ferroptosis in HL7702 cells (n=3).** HL7702 cells were treated with 10 μM Crizotinib for 0, 6, 12, 24, 48 or 72 h or treated with 0, 5, 10 and 15 μM Crizotinib for 48 h. **(A-B)** The changes of ACSL4, GPX4 protein expression treated with Crizotinib at different time (A) and concentration (B) were observed. **(C-D)** Iron content (C) and MDA (D) were measured by reagent kit following treatment with increasing concentrations of Crizotinib for 48 h. ^*^*P*<0.05, ^**^*P*<0.01 and ^***^*P*<0.001 vs. Control group.


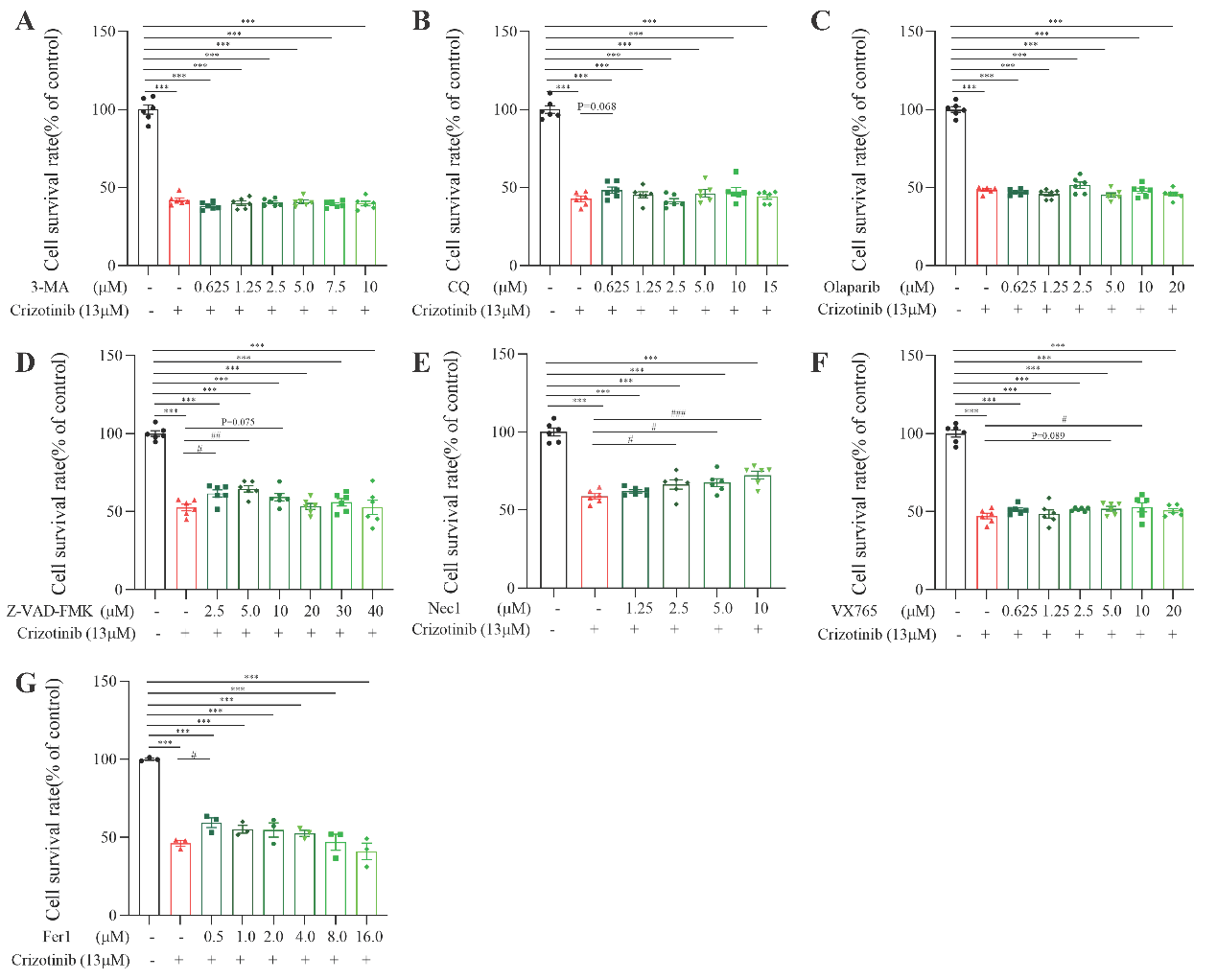


**Supplementary Figure 5. Effects of various cell death inhibitors combined with Crizotinib on cell survival in AML12 cells.** **(A-G)** The survival rate of AML12 cells were treated with 13 μM Crizotinib and/or 3-MA (A, n= 6), CQ (B, n= 6), olapanib (C, n= 6), Z-VAD-FMK (D, n= 6), Nec1 (E, n= 6), VX765 (F, n= 6) and Fer1 (G, n=3). ^*^*P*<0.05 and ^***^*P*<0.001 vs. control group. ^#^*P*<0.05, ^##^*P*<0.01 and ^###^*P*<0.001 vs. Crizotinib group.


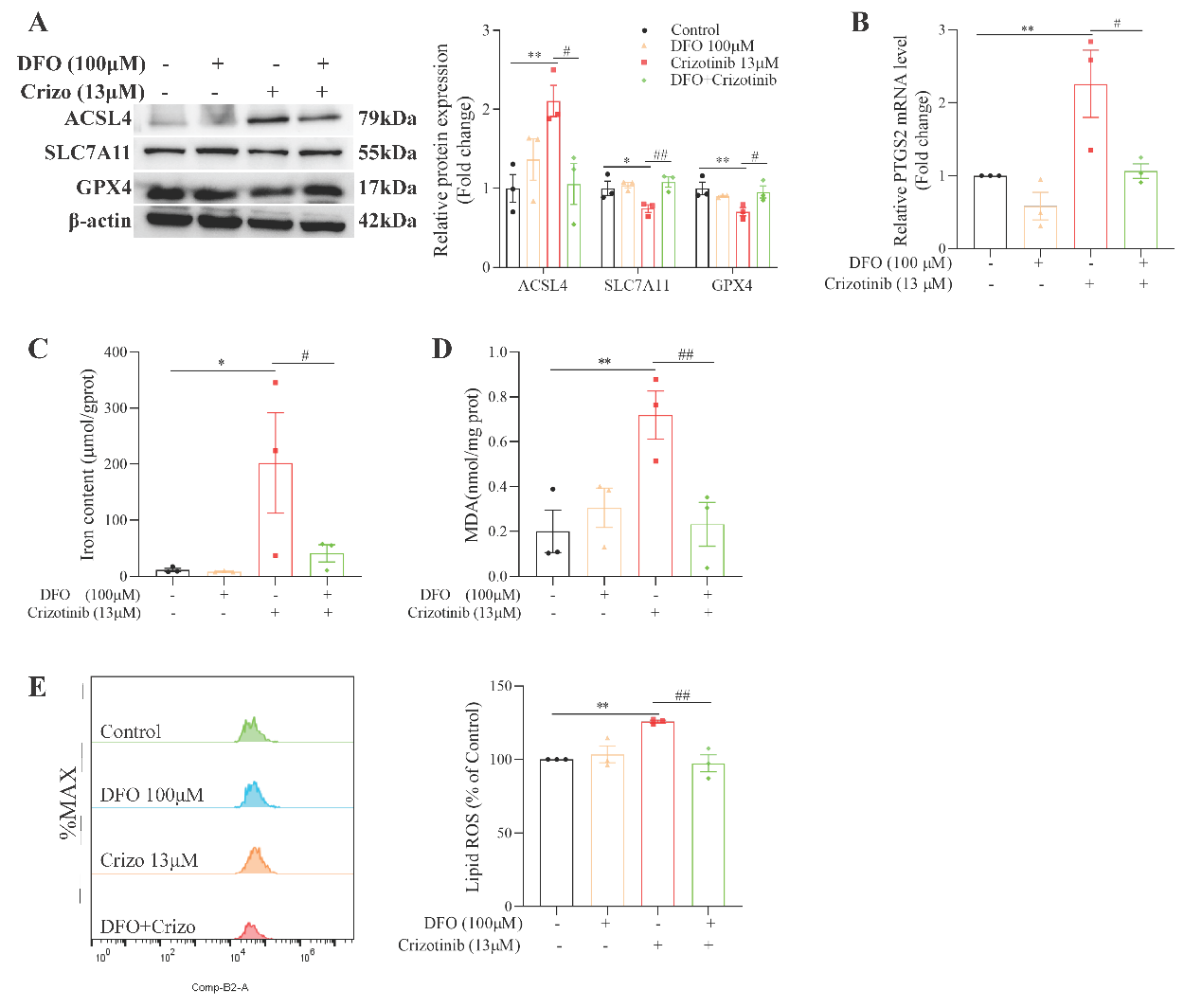


**Supplementary Figure 6. DFO alleviates Crizotinib- triggered ferroptosis in AML12 cells (n=3).** AML12 cells were pretreated with DFO for 1 h and co- incubated with Crizotinib for 48 h. **(A)** The expression of ACSL4, SLC7A11, GPX4 protein were measured by western blot. **(B)** The mRNA levels of PTGS2 were determined by RT-qPCR. **(C-D)** Iron content (C) and MDA (D) were measured by reagent kit. **(E)** The induction of lipid ROS was determined by BODIPY™ 581/591 C11 and flow cytometry. ^*^*P*<0.05, and ^**^*P*<0.01 vs. control group. ^#^*P*<0.05 and ^##^*P*<0.01 vs. Crizotinib group. Abbreviation: Crizo, Crizotinib.


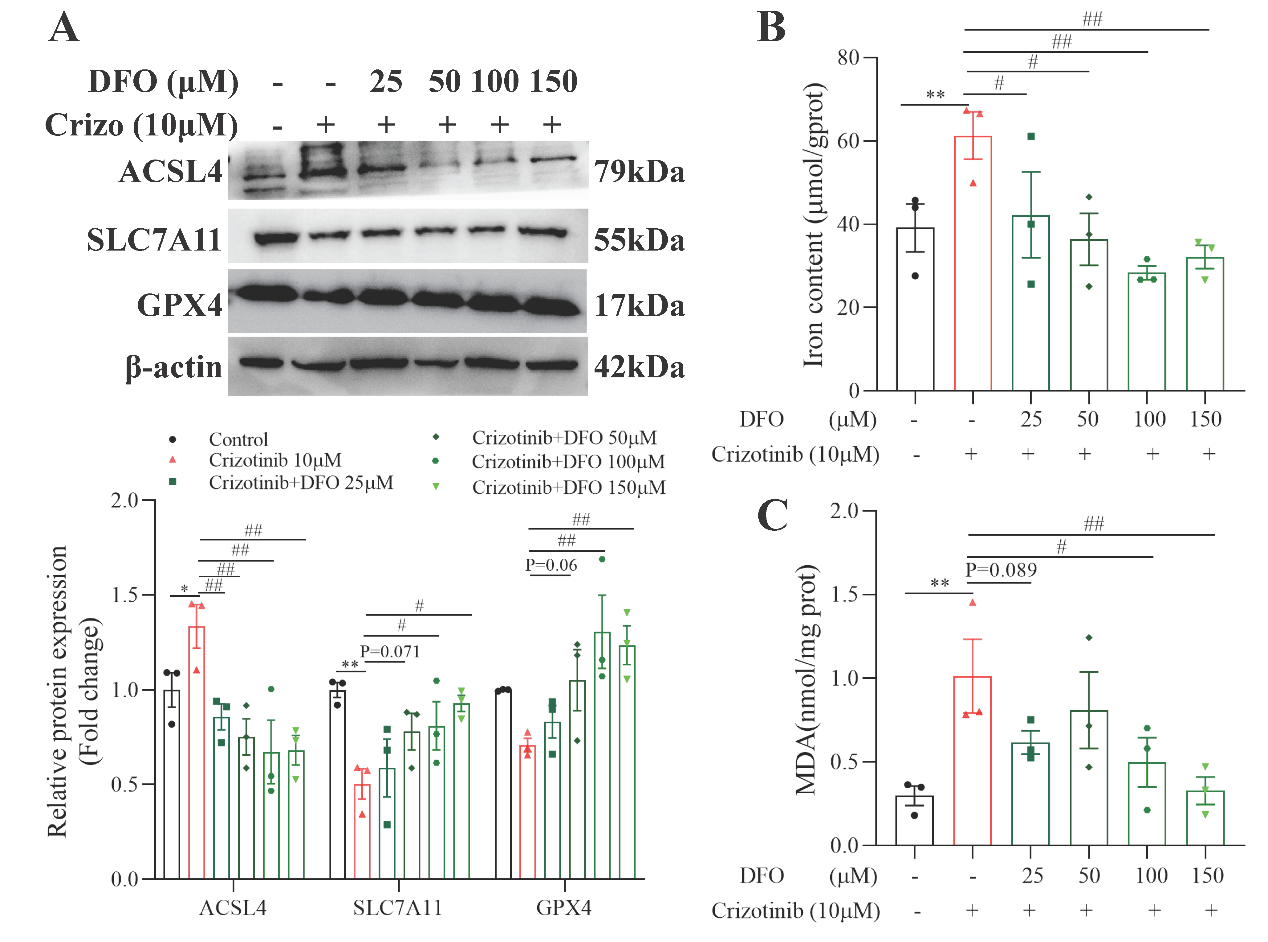


**Supplementary Figure 7. DFO alleviates Crizotinib- triggered ferroptosis in HL7702 cells (n=3).** HL7702 cells were pretreated with DFO for 1 h and co- incubated with Crizotinib for 48 h. **(A)** The expression of ACSL4, SLC7A11, GPX4 protein (A) were measured by western blot. **(B-C)** Iron content (B) and MDA (C) were measured by reagent kit. ^*^*P*<0.05, and ^**^*P*<0.01 vs. control group. ^#^*P*<0.05 and ^##^*P*<0.01 vs. Crizotinib group. Abbreviation: Crizo, Crizotinib.


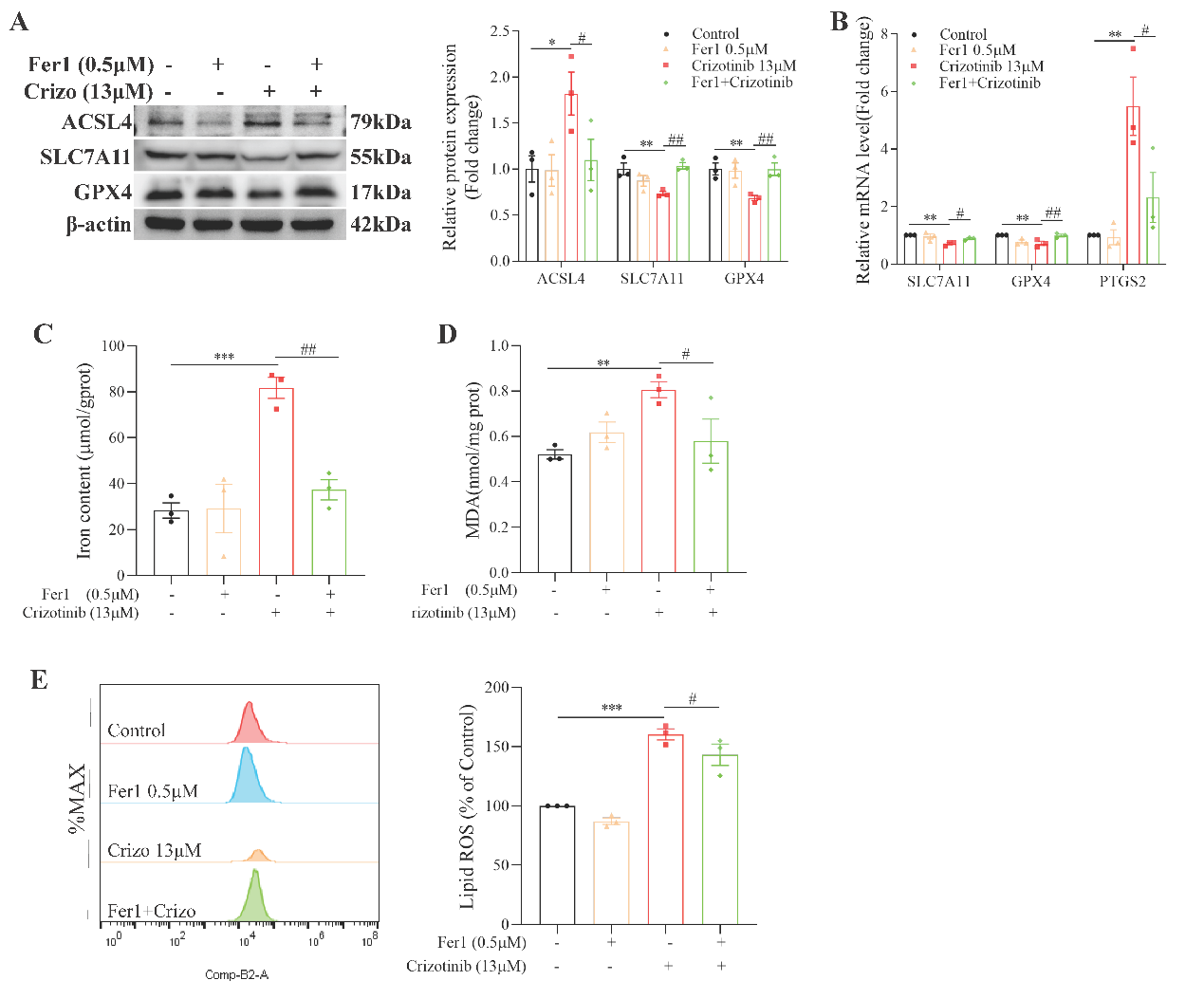


**Supplementary Figure 8. Fer1 alleviates Crizotinib- induced hepatocellular ferroptosis (n=3).** AML12 cells were pretreated with 0.5 μM Fer1 for 1 h and co- incubated with Crizotinib for 48 h. **(A-B)** The changes of ACSL4, SLC7A11, GPX4 protein expression (A) and SLC7A11, GPX4 and PTGS2 mRNA levels (B) were observed. **(C-D)** Iron content (C) and MDA (D) were measured by reagent kit. **(E)** The induction of lipid ROS was determined by BODIPY™ 581/591 C11 and flow cytometry. ^*^*P*<0.05, ^**^*P*<0.01 and ^***^*P*<0.001 vs. control group. ^#^*P*<0.05 and ^##^*P*<0.01 vs. Crizotinib group. Abbreviation: Crizo, Crizotinib.


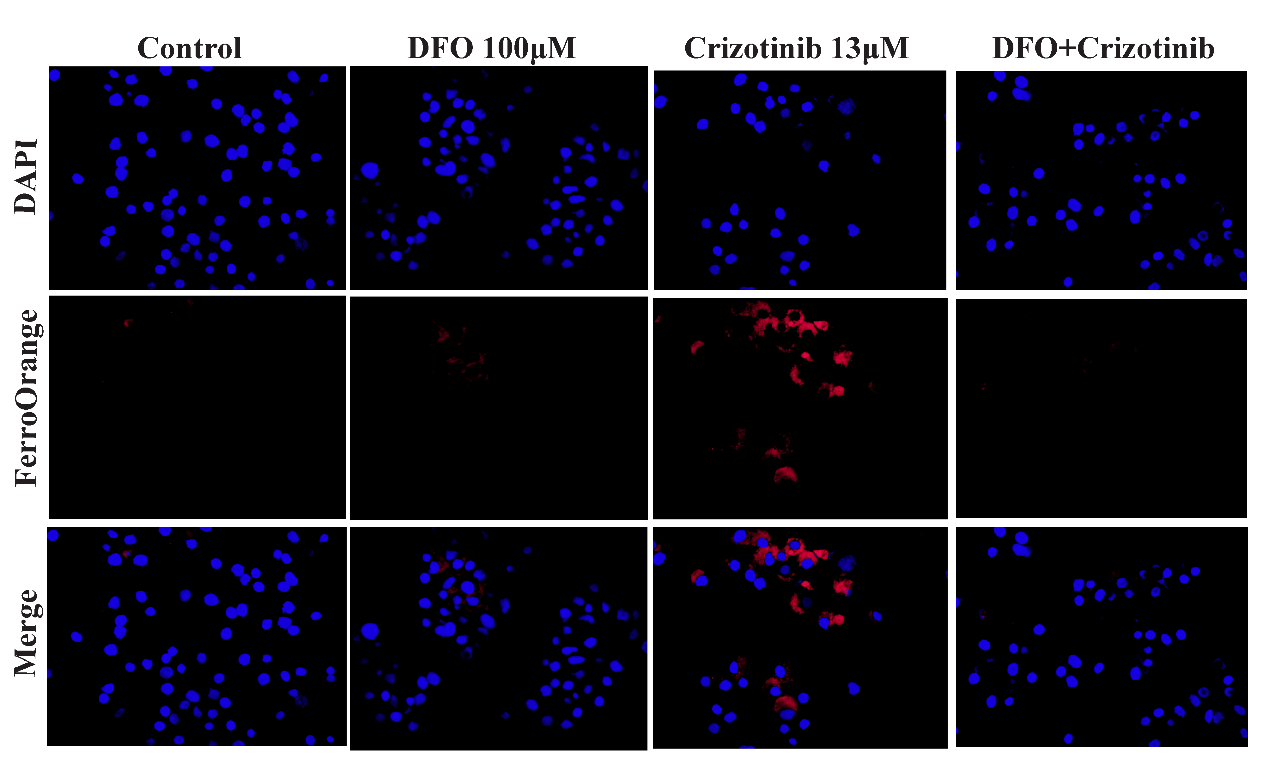


**Supplementary Figure 9. DFO alleviates Crizotinib- induced Fe^2+^ accumulation in AML12 cells detected by FerroOrange probe (20×, n=3).** AML12 cells were pretreated with 100 μM DFO for 1 h and co- incubated with 13 μM Crizotinib for 48 h.


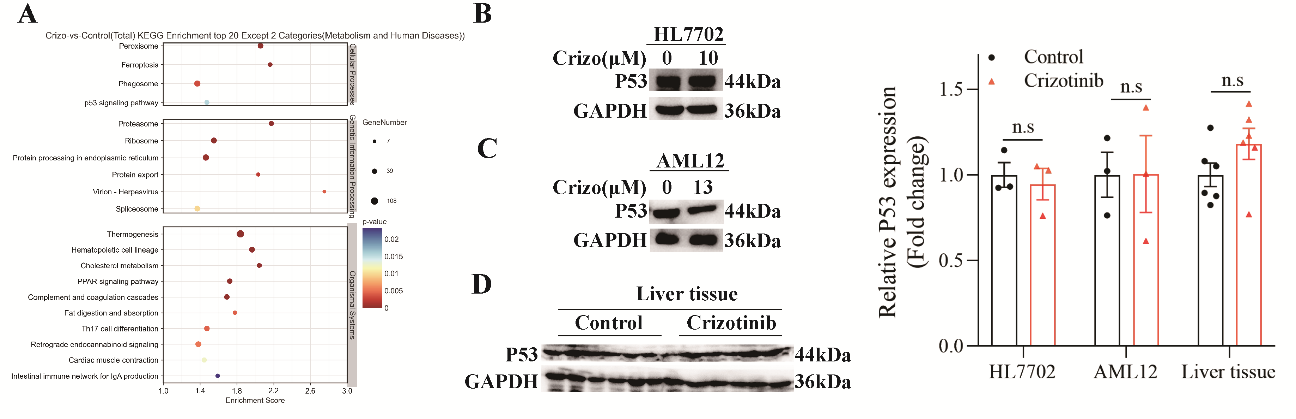


**Supplementary Figure 10.** **Effects of Crizotinib on the P53 pathway.** **(A)** top 20 KEGG pathway (except 2 categories (Metabolism and Human Diseases)) for the DEGs between the control and Crizotinib group. **(B-D)** The expression level of P53 proteins in HL7702 cells (B, n= 3), AML12 cells (C, n= 3) and liver tissues (D, n= 6) after Crizotinib treatment was analyzed by western blot. n.s, no signification. Abbreviation: Crizo, Crizotinib.


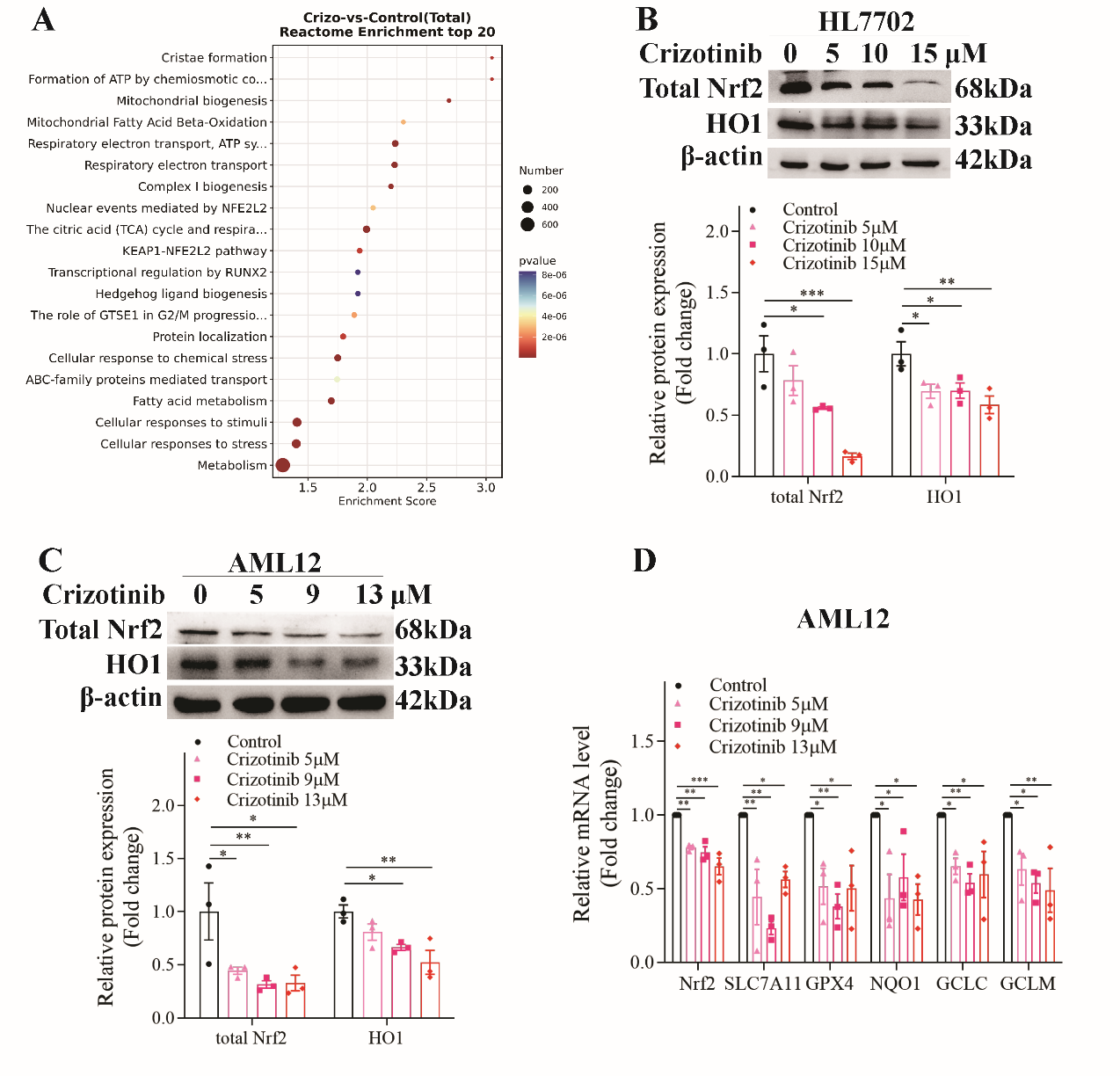


**Supplementary Figure 11. Effects of Crizotinib on the Nrf2 pathway.** **(A)** Signification enriched top 20 Reactome for the DEGs between the control and Crizotinib group. **(B-C)** The expression level of Nrf2 and HO1 proteins in HL7702 cells (B) and AML12 cells (C) treated with Crizotinib at different concentrations for 48 h was analyzed by western blot (n=3). **(D)** The mRNA levels of Nrf2 and its downstream gene in AML12 cells (n=3) were determined by RT-qPCR. ^*^*P*<0.05, ^**^*P*<0.01 and ^***^*P*<0.001 vs. control group. Abbreviation: Crizo, Crizotinib.


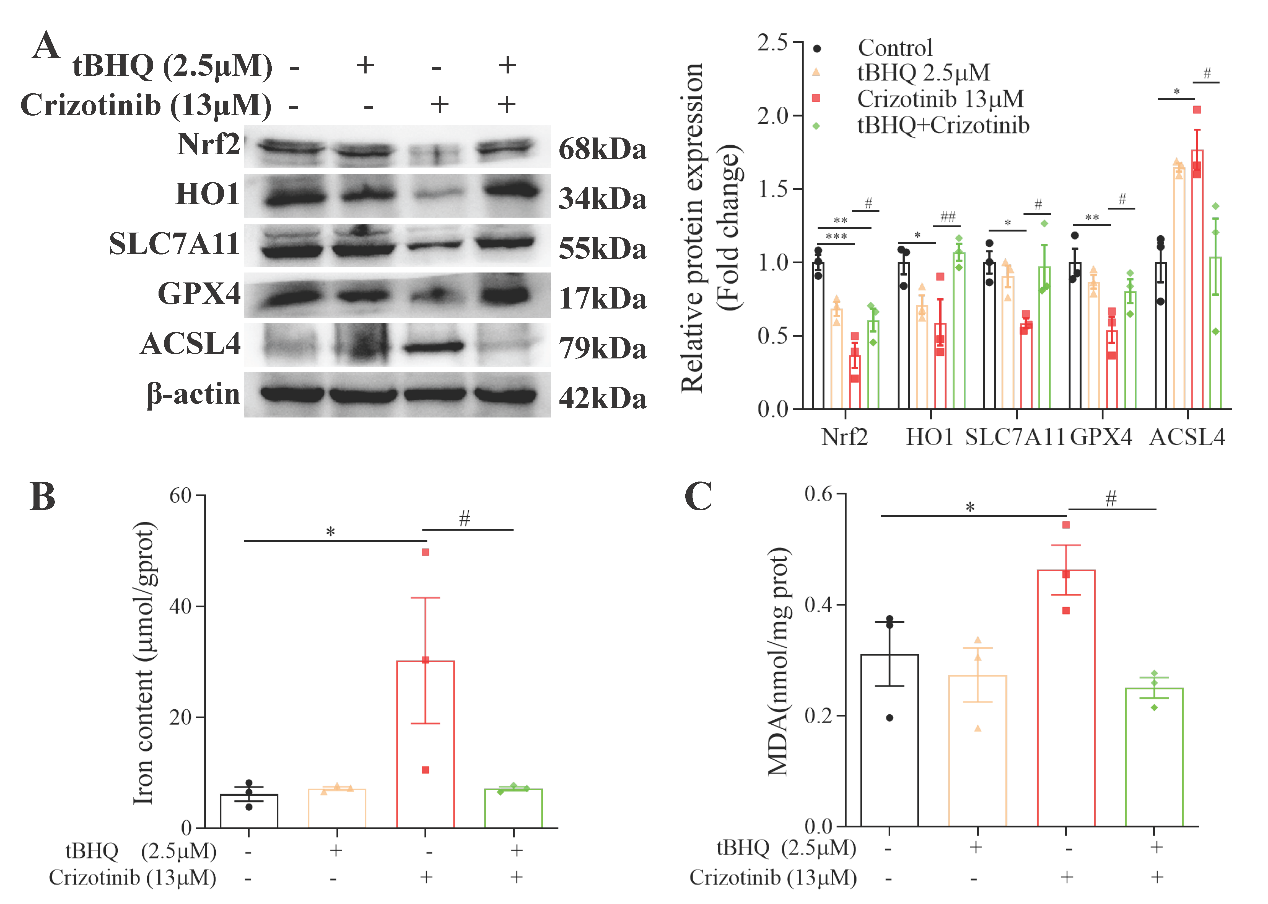


**Supplementary Figure 12. Activation of Nrf2 by tBHQ treatment attenuated the severity of Crizotinib- induced ferroptosis in AML12 cells (n=3).** AML12 cells were pretreated with 2.5 μM tBHQ for 1 h and co- incubated with Crizotinib for 48 h. **(A)** Protein expression of Nrf2, HO1, SLC7A11, GPX4 and ACSL4 was measured by western blot. **(B)** Fold change in Iron content. **(C)** Fold change in MDA level. ^*^*P*<0.05, ^**^*P*<0.01 and ^***^*P*<0.001 vs. control group. ^#^*P*<0.05, ^##^*P*<0.01 and ^###^*P*<0.001 vs. Crizotinib group.


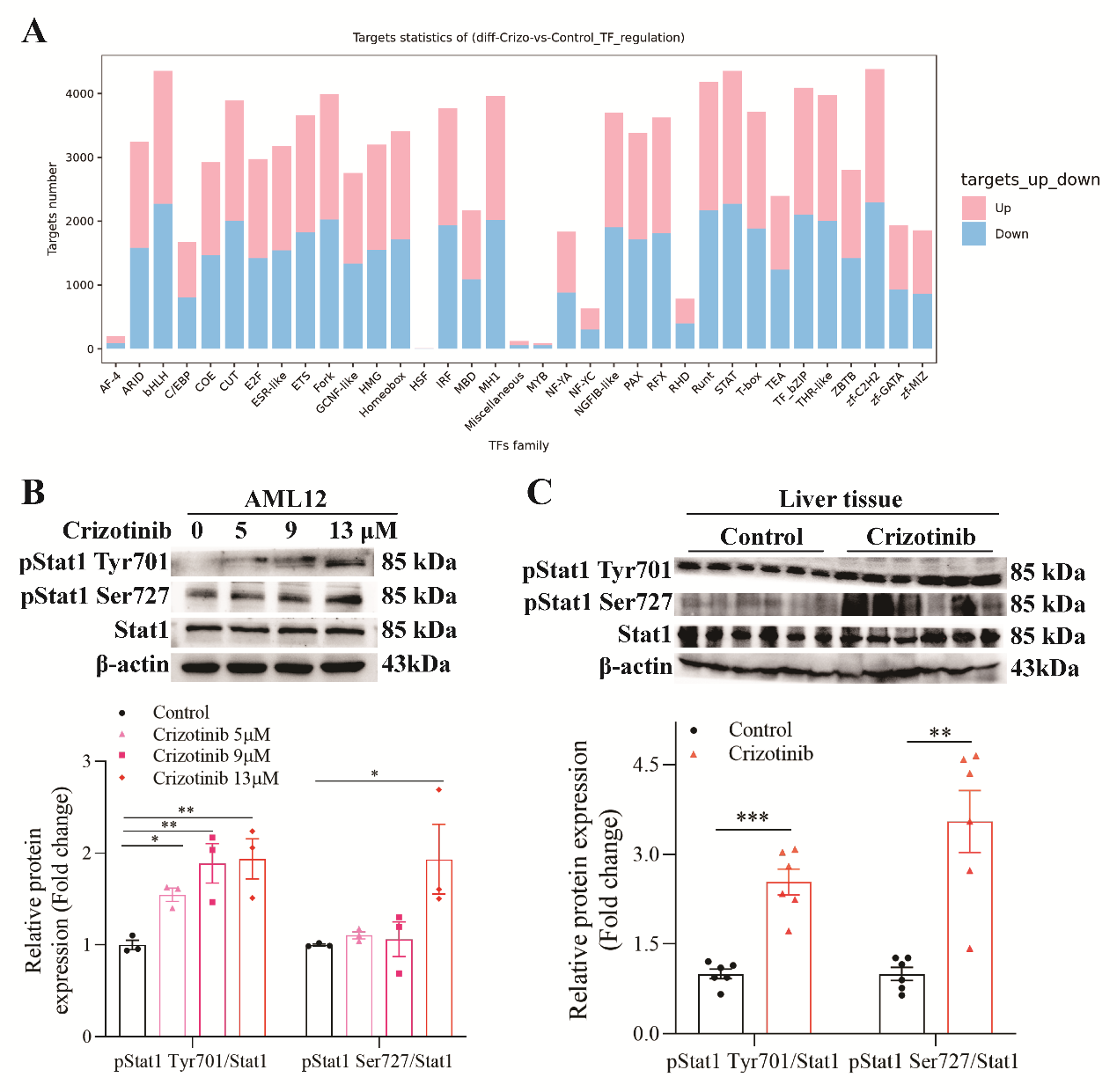


**Supplementary Figure 13. Crizotinib induced Stat1 phosphorylation.** **(A)** Distribution of TF family target genes in differential genes. **(B-C)** The levels of Stat1, pStat1 Tyr701 and Ser727 in AML12 cells (B, n=3) and liver tissue (C, n=6). ^*^P<0.05, ^**^P<0.01 and ^***^P<0.001 vs. control group.


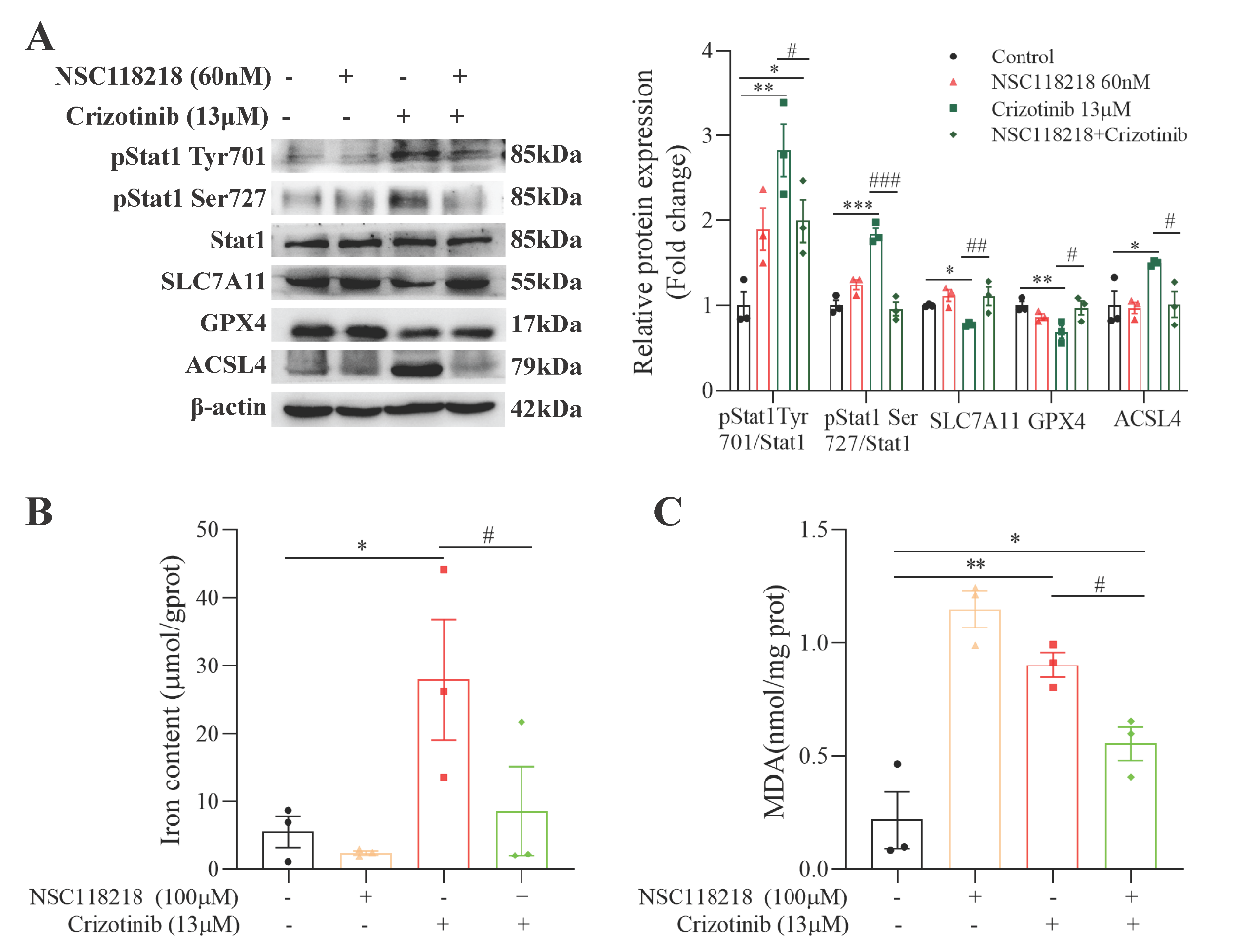


**Supplementary Figure 14. Silence of Stat1 by NSC118218 treatment attenuated the severity of Crizotinib- induced ferroptosis in AML12 cells (n=3).** **(A)** Protein expression of pStat1 Tyr701, pStat1 Ser727, Stat1, SLC7A11, GPX4 and ACSL4 was measured by western blot. **(B)** Fold change in Iron content. **(C)** Fold change in MDA level. ^*^P<0.05, ^**^P<0.01 and ^***^P<0.001 vs. control group. ^#^P<0.05, ^##^P<0.01 and ^###^P<0.001 vs. Crizotinib group.


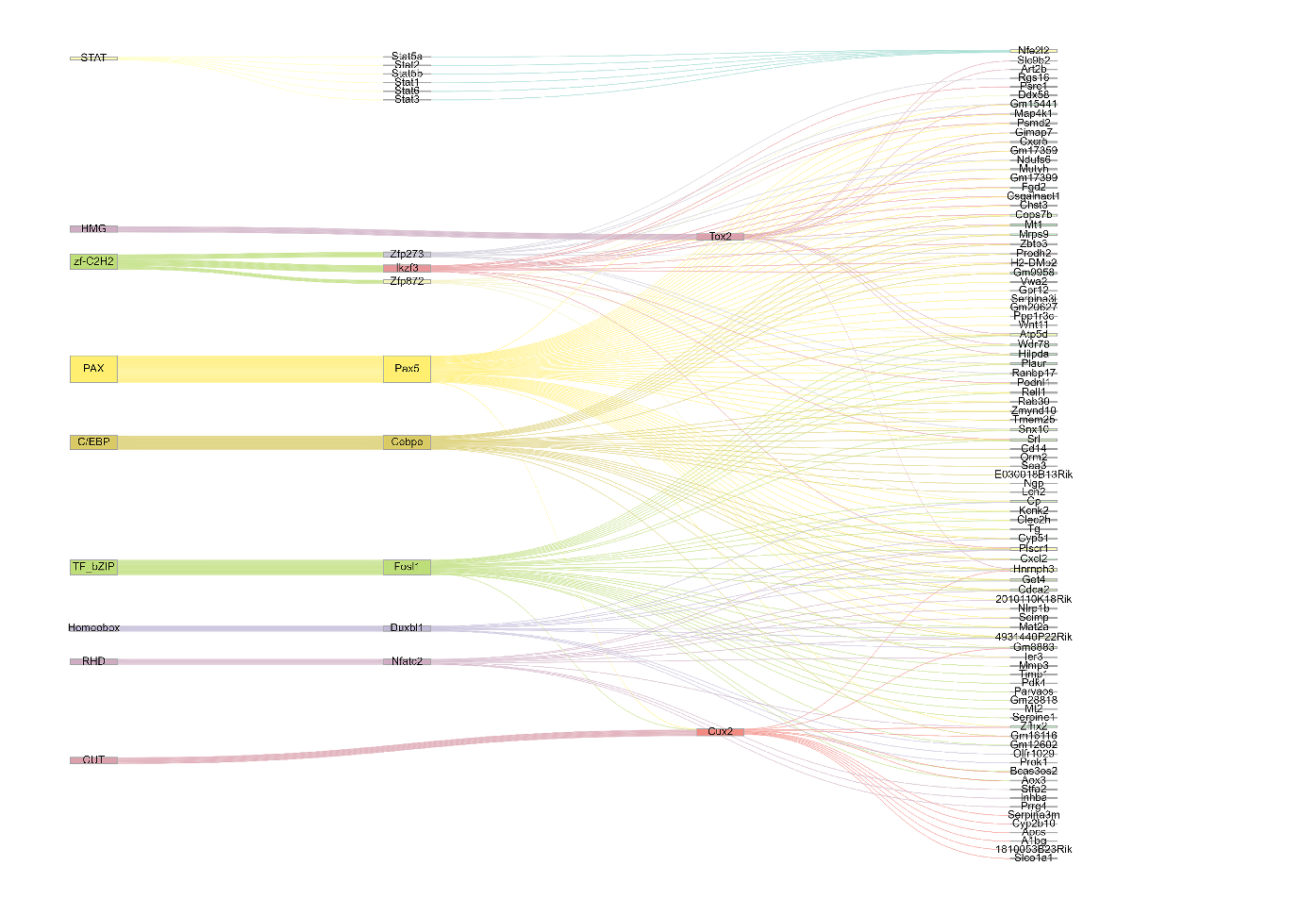


**Supplementary Figure 15. Mulberry diagram of the relationships between top 9 TF families and DEGs in RNA sequencing analysis.**


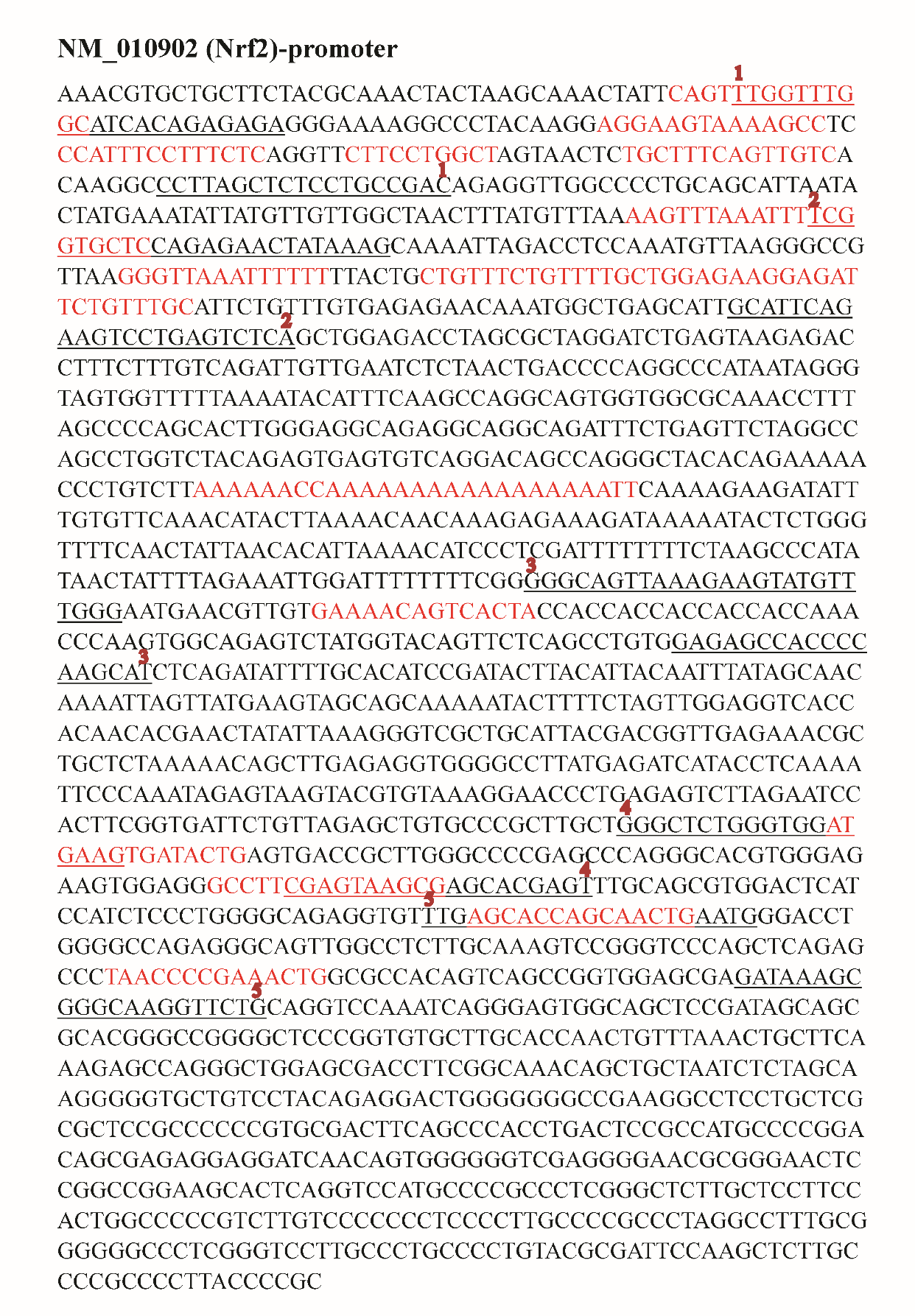


**Supplementary Figure 16. Mouse Nrf2 promoter sequence.** Red highlighting indicates the predicted sequences where Stat1 and Nrf2 bind; the underline represents the regions corresponding to the designed CHIP qPCR primers.


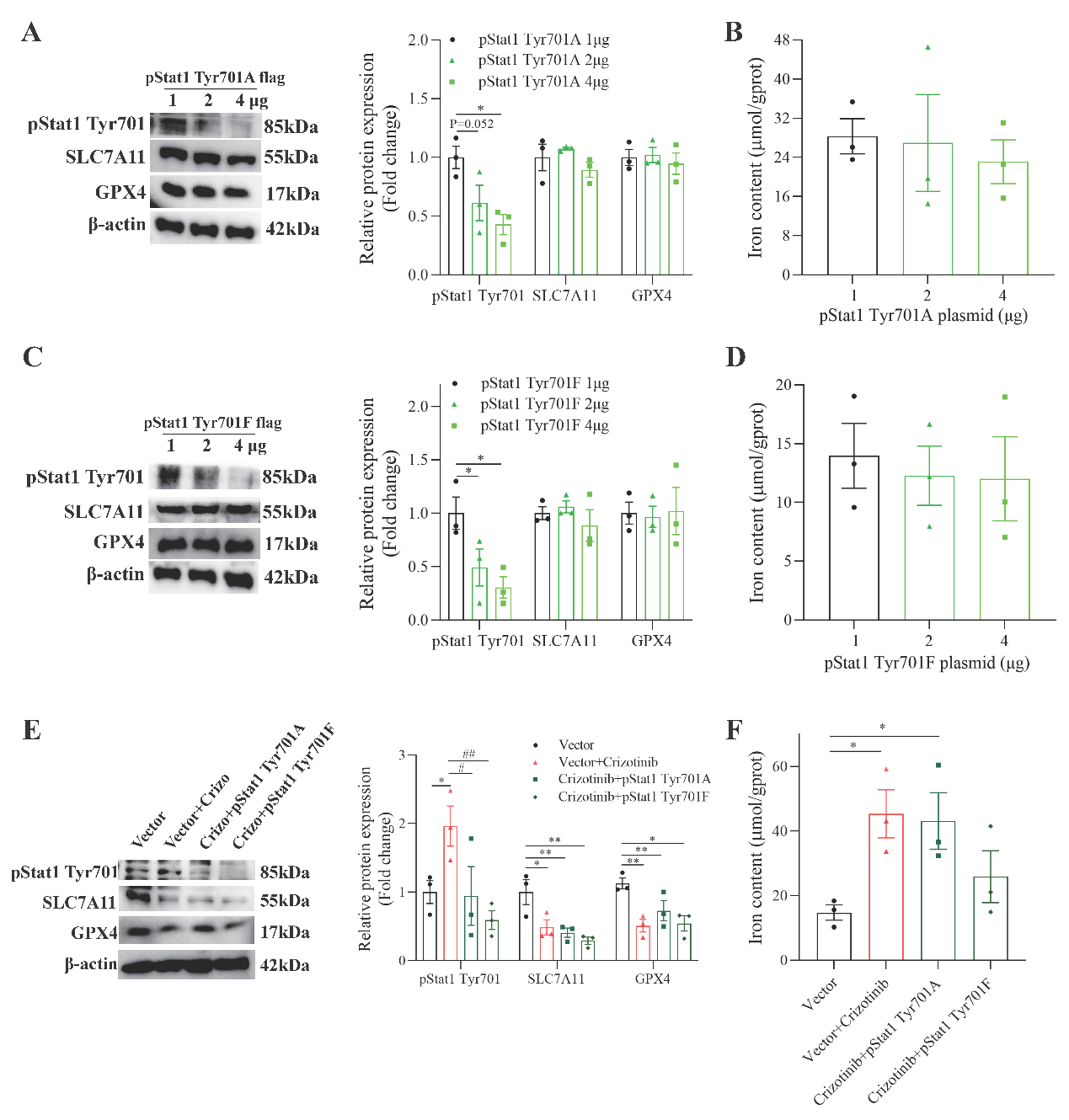


**Supplementary Figure 17. Crizotinib- induced ferroptosis independent of pStat1 Tyr701 in AML12 cells (n= 3).** AML12 cells were transfected with 1, 2 or 4 μg Stat1 Tyr701A or F plasmid for 48 h. **(A)** Protein expression of pStat1 Tyr701, SLC7A11 and GPX4 was measured by western blot. **(B)** Fold change in Iron content. AML12 cells were transfected with 4 μg vector or Stat1 Tyr701A or F plasmid, followed by treatment with or without 13 μM Crizotinib for 48 h. **(C)** The expression levels of pStat1 Tyr701, SLC7A11 and GPX4 were analyzed by western blot. **(D)** Fold change in Iron content. ^*^P<0.05, ^**^P<0.01 and ^***^P<0.001 vs. control group. ^#^P<0.05, ^##^P<0.01 and ^###^P<0.001 vs. Crizotinib group.


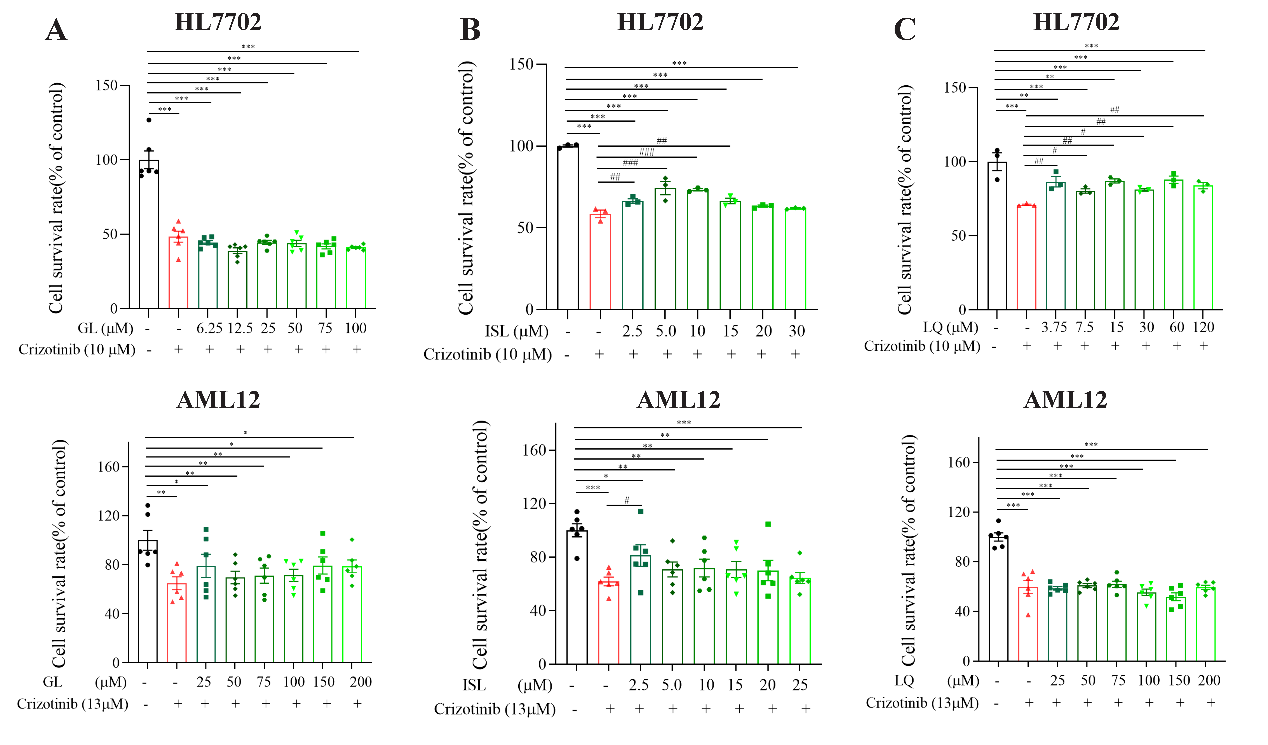


**Supplementary Figure 18. Effects of glycyrrhiza monomers combined with Crizotinib on cell survival in HL7702 cell and AML12 cells** (n=3 or 6). **(A)** GL; **(B)** ISL; **(C)** LQ. ^*^P<0.05, ^**^P<0.01 and ^***^P<0.001 vs. control group. ^#^P<0.05, ^##^P<0.01 and ^###^P<0.001 vs. Crizotinib group.


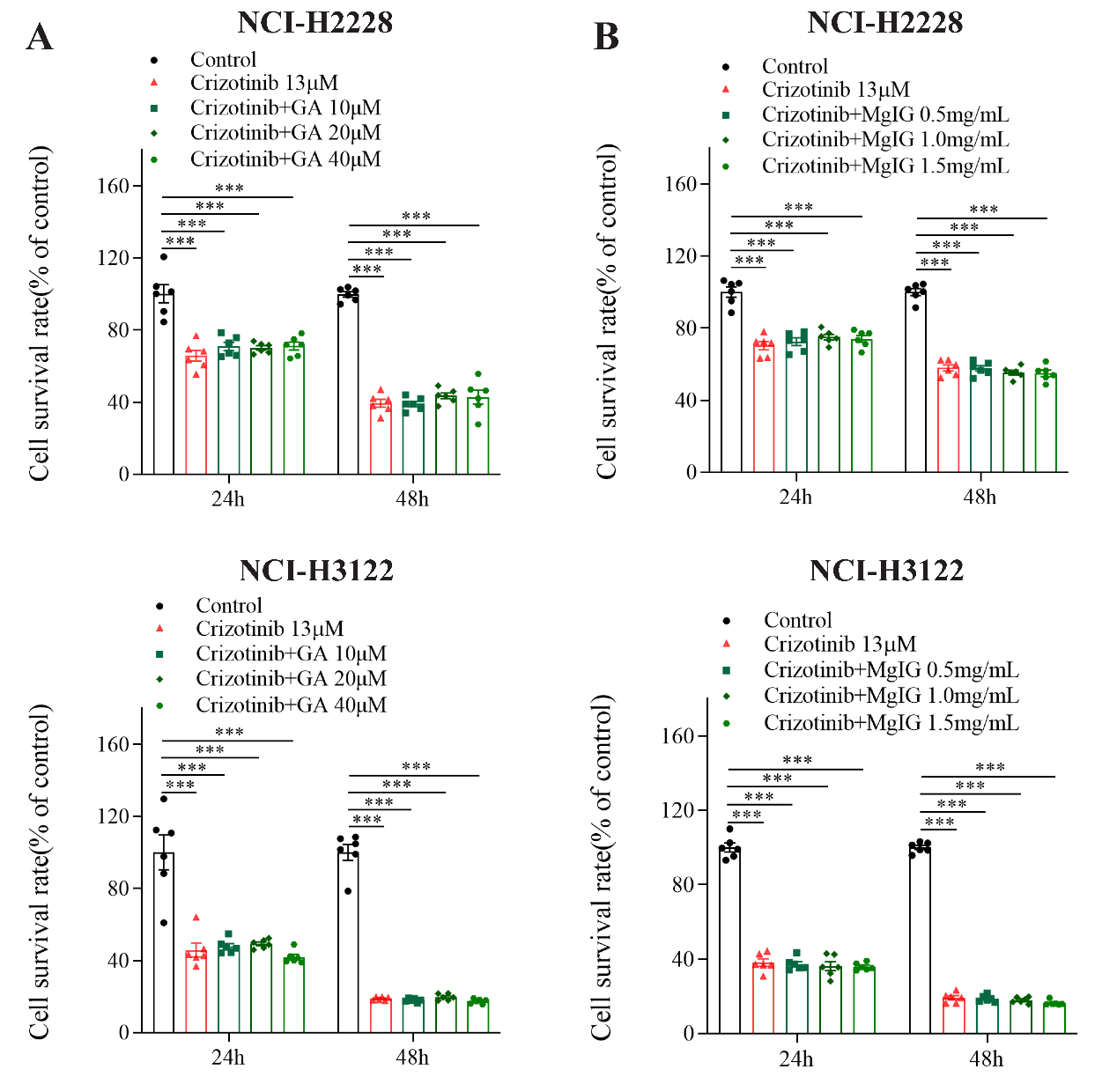


**Supplementary Figure 19. Effect of GA and MgIG combined with Crizotinib on the cell survival rate of ALK^+^ NSCLC cell lines (n=6).** **(A)** GA in NCI-H2228 and NCI-H3122 cell. **(B)** MgIG in NCI-H2228 and NCI-H3122 cell. ^***^P<0.001 vs. Control group.


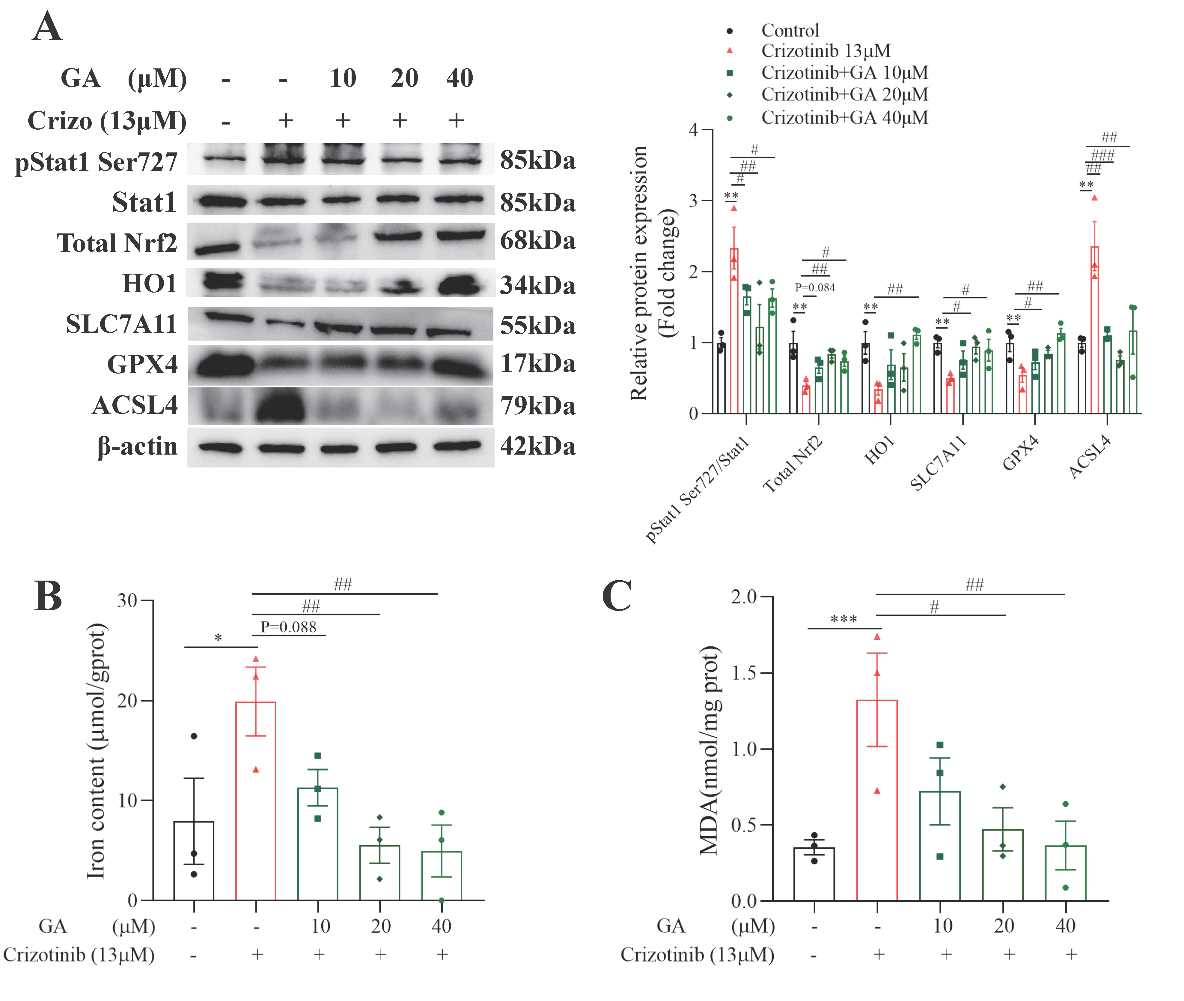


**Supplementary Figure 20. Effects of GA combined with Crizotinib on Stat1/Nrf2 pathway and ferroptosis in AML12 cells (n=3).** AML12 cells were pretreated with GA for 6 h and co-incubated with Crizotinib for 48 h. **(A)** The expression of pStat1 Ser727, Stat1, Nrf2, HO1, SLC7A11, GPX4 and ACSL4 protein were measured by western blot. **(B-C)** Iron content (B) and MDA (C) were measured by reagent kit. ^*^P<0.05, ^**^P<0.01 and ^***^P<0.001 vs. Control group. ^#^P<0.05, ^##^P<0.01 and ^###^P<0.001 vs. Crizotinib group. Abbreviation: Crizo, Crizotinib.


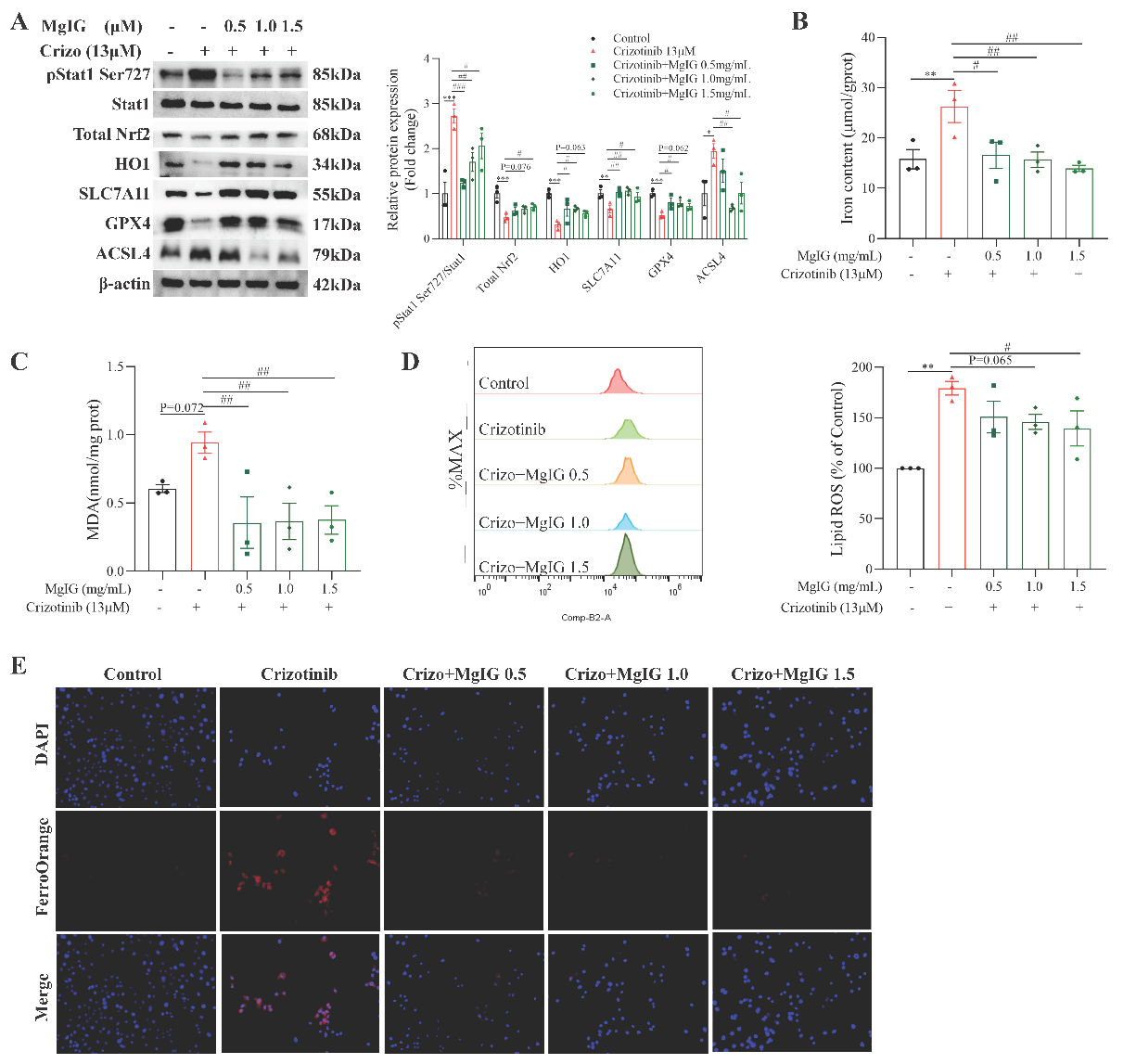


**Supplementary Figure 21. Effects of MgIG combined with Crizotinib on Stat1/Nrf2 pathway and ferroptosis in AML12 cells (n=3).** AML12 cells were pretreated with MgIG for 6 h and co-incubated with Crizotinib for 48 h. **(A)** The expression of pStat1 Ser727, Stat1, Nrf2, HO1, SLC7A11, GPX4 and ACSL4 protein were measured by western blot. **(B-C)** Iron content (B) and MDA (C) were measured by reagent kit. **(D)** The induction of lipid ROS was determined by BODIPY™ 581/591 C11 and flow cytometry. **(E)** Fe2+ was detected by FerroOrange probe (20×). ^*^P<0.05, ^**^P<0.01 and ^***^P<0.001 vs. Control group. ^#^P<0.05, ^##^P<0.01 and ^###^P<0.001 vs. Crizotinib group. Abbreviation: Crizo, Crizotinib.


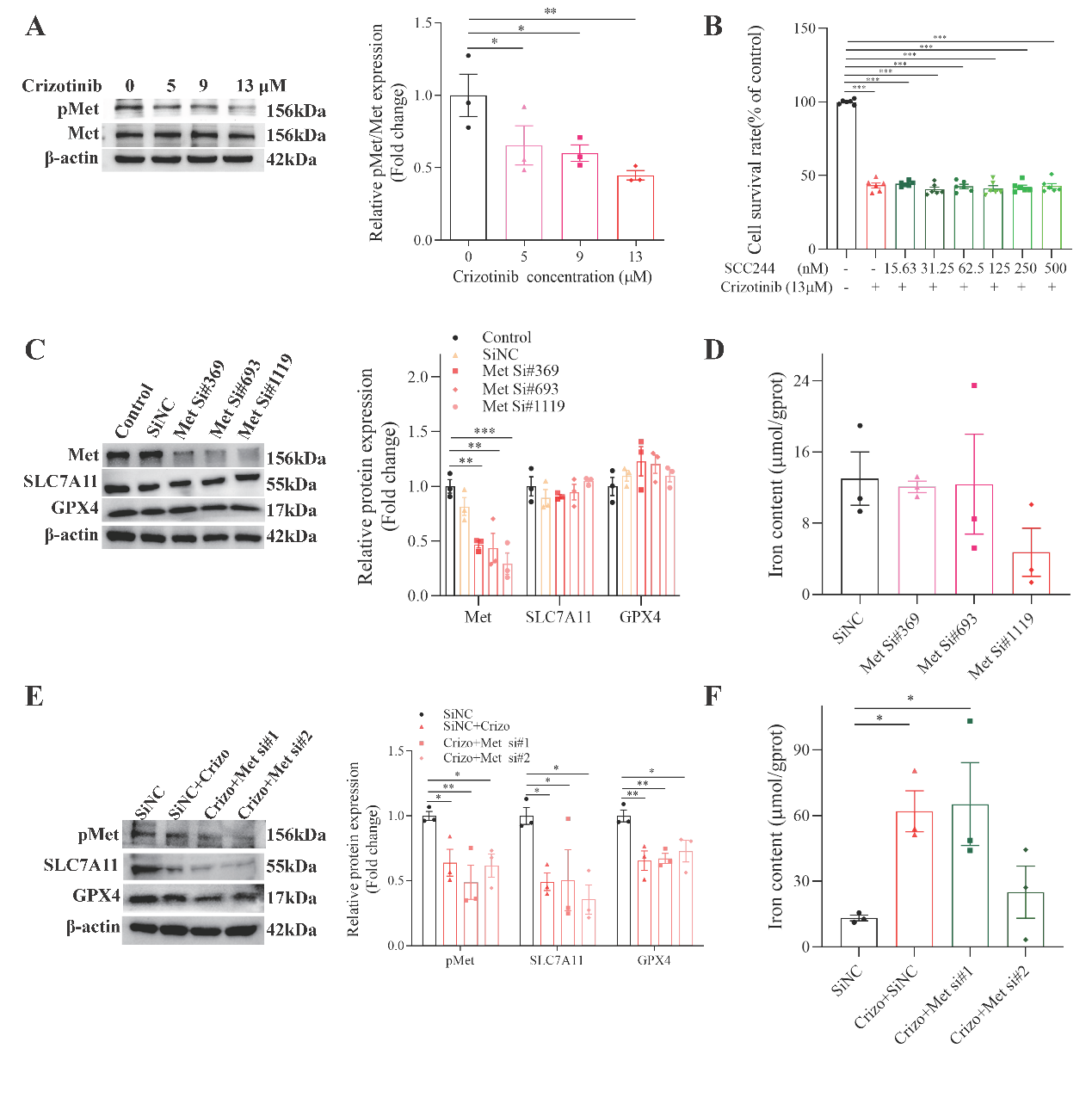
 **Supplementary Figure 22. Crizotinib- induced ferroptosis independent of Met in AML12 cells (n= 3).** AML12 cells were treated with 0, 5, 9 or 13 μM Crizotinib for 48 h. **(A)** The expression level of pMet and Met was measured by western blot. **(B)** Fold change in Iron content. AML12 cells were transfected with 100 ng Met SiRNA for 48 h. **(C)** The expression level of Met, SLC7A11 and GPX4 was measured by western blot. **(D)** Fold change in Iron content. AML12 cells were transfected with 4 μg vector or Stat1 Tyr701A or F plasmid, followed by treatment with or without 13 μM Crizotinib for 48 h. **(E)** The expression level of Met, SLC7A11 and GPX4 was measured by western blot. **(F)** Fold change in Iron content. ^*^P<0.05, ^**^P<0.01 and ^***^P<0.001 vs. control group. ^#^P<0.05, ^##^P<0.01 and ^###^P<0.001 vs. Crizotinib group. Abbreviation: Crizo: Crizotinib. Met Si#693: Met Si#1. Met Si#1119: Met Si#2.


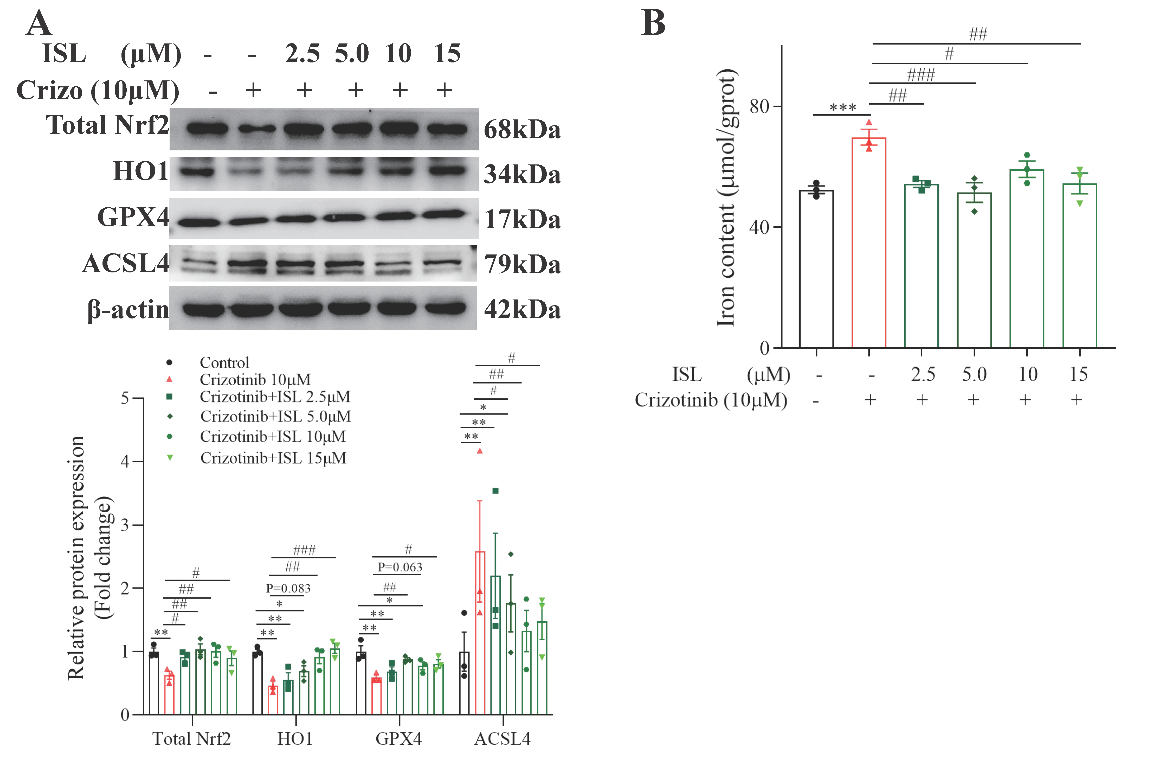


**Supplementary Figure 23. Effects of ISL combined with Crizotinib on Nrf2 pathway and ferroptosis in HL7702 cells (n=3). (A)** Protein expression of total Nrf2, HO1, GPX4 and ACSL4 was measured by western blot. **(B)** Determination of Iron content. ^*^P<0.05, ^**^P<0.01 and ^***^P<0.001 vs. Control group. ^#^P<0.05, ^##^P<0.01 and ^###^P<0.001 vs. Crizotinib group. Abbreviation: Crizo, Crizotinib.

**Supplementary Tables**

**Supplementary Table 1. Four-cell table for measurement of ratio imbalance**

| **Drugs** | **Target AEs** | **Other AEs** | **Total** |
| --- | --- | --- | --- |
| TKIs | A | B | A+B |
| Other Drugs | C | D | C+D |
| Total | A+C | B+D | A+B+C+D |

**Supplementary Table 2. The primer sequences of quantitative RT-qPCR**

| **Gene** | **Forward** | **Reverse** |
| --- | --- | --- |
| mus-PTGS2 | CTGGTGCCTGGTCTGATGATGTATG | GGATGCTCCTGCTTGAGTATGTCG |
| mus-SLC7A11 | CATTGTATGGGACAAGAAACC | GGCACTAGACTCAAGAACTGTG |
| mus-GPX4 | AATTCTCAGCCAAGGACATCGAC | ACACGAAACCCCTGTACTTATCCAG |
| mus-Nrf2 | AAGCACAGCCAGCACATTCTCC | TGACCAGGACTCACGGGAACTTC |
| mus-GCLC | GCACATCTACCACGCAGTCAAGG | TCTCAAGAACATCGCCTCCATTCAG |
| mus-GCLM | ACAATGACCCGAAAGAACTGCTCTC | TCTTCACGATGACCGAGTACCTCAG |
| mus-FPN | TGGAACTCTATGGAAACAGCCT | TGGCATTCTTATCCACCCAGT |
| mus-FTH1 | CCATCAACCGCCAGATCAACCTG | GCAAAGTTCTTCAGAGCCACATCATC |
| mus-GAPDH | TCACCATCTTCCAGGAGCGAGAC | TGAGCCCTTCCACAATGCCAAAG |

mus represents mouse;

**Supplementary Table 3. The sequences of siRNAs**

| **Name** | **Forward** | **Reverse** |
| --- | --- | --- |
| mmu-siMet-#1 (693) | GUCCUAUAUUGAUGUCUUATT | UAAGACAUCAAUAUAGGACTT |
| mmu-siMet-#2 (1119) | CAACAAGAUUGUCAACAAATT | UUUGUUGACAAUCUUGUUGTT |
| mmu-siMet-#3 (369) | CACAUACUAUGAUGAUCAATT | UUGAUCAUCAUAGUAUGUGTT |
| mmu-siStat1-#1 (850) | CAGAGAAGGUGAAGCCAAUTT | AUUGGCUUCACCUUCUCUGTT |
| mmu-siStat1-#2 (1470) | ACACAAAAGUGAUGAACAUTT | AUGUUCAUCACUUUUGUGUTT |
| mmu-siStat1-#3 (729) | AGAAGGAGCUGGACAGUAATT | UUACUGUCCAGCUCCUUCUTT |
| mmu-siNC | UUCUCCGAACGUGUCACGUTT | ACGUGACACGUUCGGAGAATT |

mus represents mouse;

**Supplementary Table 4. Instrument operating parameter for Iron ion concentration detection by ICP-MS**

| **parameter** | **Value** |
| --- | --- |
| Atomizer flow | 1.04 L/min |
| Auxiliary gas flow | 1.20 L/min |
| Plasma gas flow | 15 L/min |
| Radio-frequency power | 1600 W |
| Analog voltage | -1862 V |
| Impulse voltage | 1250 V |
| Limit of quantitation | 9.16 μg/L |

**Supplementary Table 5. qPCR primers for ChIP assays**

| **Name** | **Forward** | **Reverse** |
| --- | --- | --- |
| mus-Nrf2-#1 | TTGGTTTGGCATCACAGAGAGA | GTCGGCAGGAGAGCTAAGG |
| mus-Nrf2-#2 | TCGGTGCTCCAGAGAACTATAAAG | TGAGACTCAGGACTTCTGAATGC |
| mus-Nrf2-#3 | GGGCAGTTAAAGAAGTATGTTTGGG | ATGCTTGGGGTGGCTCTC |
| mus-Nrf2-#4 | GGGCTCTGGGTGGATGAAG | ACTCGTGCTCGCTTACTCG |
| mus-Nrf2-#5 | TTGAGCACCAGCAACTGAATG | CAGAACCTTGCCCGCTTTATC |

mus represents mouse;

**Supplementary Table 6. JASPAR Prediction results**

| **Matrix ID** | **Name** | **Score** | **Relative Score** | **Start** | **End** | **Strand** | **Predicted sequence** |
| --- | --- | --- | --- | --- | --- | --- | --- |
| [**MA0137.1**](https://jaspar.genereg.net/matrix/MA0137.1) | MA0137.1.STAT1 | 9.810686 | 0.7940530476430773 | 242 | 255 | - | GAAAATTTAAACTT |
| [**MA0137.1**](https://jaspar.genereg.net/matrix/MA0137.1) | MA0137.1.STAT1 | 9.522073 | 0.7880434737813594 | 332 | 345 | - | CAAAACAGAAACAG |
| [**MA0137.1**](https://jaspar.genereg.net/matrix/MA0137.1) | MA0137.1.STAT1 | 8.622327 | 0.7693087539094835 | 1277 | 1290 | + | ATGAAGTGATACTG |
| [**MA0137.1**](https://jaspar.genereg.net/matrix/MA0137.1) | MA0137.1.STAT1 | 8.179393 | 0.7600858780827401 | 40 | 53 | - | GCCAAACCAAACTG |
| [**MA0137.1**](https://jaspar.genereg.net/matrix/MA0137.1) | MA0137.1.STAT1 | 7.4618354 | 0.7451447298840848 | 138 | 151 | - | GACAACTGAAAGCA |
| [**MA0137.1**](https://jaspar.genereg.net/matrix/MA0137.1) | MA0137.1.STAT1 | 7.389271 | 0.7436337728161141 | 101 | 114 | - | GAGAAAGGAAATGG |
| [**MA0137.1**](https://jaspar.genereg.net/matrix/MA0137.1) | MA0137.1.STAT1 | 7.3822265 | 0.7434870944389061 | 355 | 368 | - | GCAAACAGAATCTC |
| [**MA0137.1**](https://jaspar.genereg.net/matrix/MA0137.1) | MA0137.1.STAT1 | 6.528748 | 0.7257157674419025 | 85 | 98 | + | AGGAAGTAAAAGCC |
| [**MA0137.1**](https://jaspar.genereg.net/matrix/MA0137.1) | MA0137.1.STAT1 | 5.956111 | 0.7137921845584199 | 1482 | 1495 | + | TAACCCCGAAACTG |
| [**MA0137.2**](https://jaspar.genereg.net/matrix/MA0137.2) | MA0137.2.STAT1 | 5.8205576 | 0.7756057124282436 | 1338 | 1352 | - | CGCTTACTCGAAGGC |
| [**MA0137.2**](https://jaspar.genereg.net/matrix/MA0137.2) | MA0137.2.STAT1 | 5.813716 | 0.775495264501949 | 340 | 354 | - | CTTCTCCAGCAAAAC |
| [**MA0137.1**](https://jaspar.genereg.net/matrix/MA0137.1) | MA0137.1.STAT1 | 5.697829 | 0.7084141726608137 | 886 | 899 | + | GAAAACAGTCACTA |
| [**MA0137.1**](https://jaspar.genereg.net/matrix/MA0137.1) | MA0137.1.STAT1 | 5.6222415 | 0.7068402768374703 | 312 | 325 | - | AAAAAATTTAACCC |
| [**MA0137.1**](https://jaspar.genereg.net/matrix/MA0137.1) | MA0137.1.STAT1 | 5.6034856 | 0.7064497368463312 | 249 | 262 | - | GAGCACCGAAAATT |
| [**MA0137.1**](https://jaspar.genereg.net/matrix/MA0137.1) | MA0137.1.STAT1 | 5.4842954 | 0.7039679303637681 | 686 | 699 | + | AAAAAAAAAAAATT |
| [**MA0137.1**](https://jaspar.genereg.net/matrix/MA0137.1) | MA0137.1.STAT1 | 5.4842954 | 0.7039679303637681 | 687 | 700 | + | AAAAAAAAAAATTC |
| [**MA0137.1**](https://jaspar.genereg.net/matrix/MA0137.1) | MA0137.1.STAT1 | 5.447077 | 0.7031929566024323 | 673 | 686 | + | AAAAAACCAAAAAA |
| [**MA0137.1**](https://jaspar.genereg.net/matrix/MA0137.1) | MA0137.1.STAT1 | 5.3628616 | 0.7014394088087916 | 1405 | 1418 | + | AGCACCAGCAACTG |
| [**MA0137.2**](https://jaspar.genereg.net/matrix/MA0137.2) | MA0137.2.STAT1 | 5.305263 | 0.7672870853026375 | 1338 | 1352 | + | GCCTTCGAGTAAGCG |

A relative score below 0.7 is considered to indicate no binding. The strand refers to binding on the coding or non-coding DNA strand, with binding on either strand considered valid. The higher the score, the more likely the binding, with a score greater than 5 being considered a good binding affinity.
