# Supplementary material for: The involvement of the Stat1/Nrf2 pathway in exacerbating Crizotinib-induced liver injury: implications for ferroptosis
Source: Cell Death Dis. 2024 Aug 19;15(8):600. doi: 10.1038/s41419-024-06993-z (PMC11333746; doi:10.1038/s41419-024-06993-z)

Figure 2B

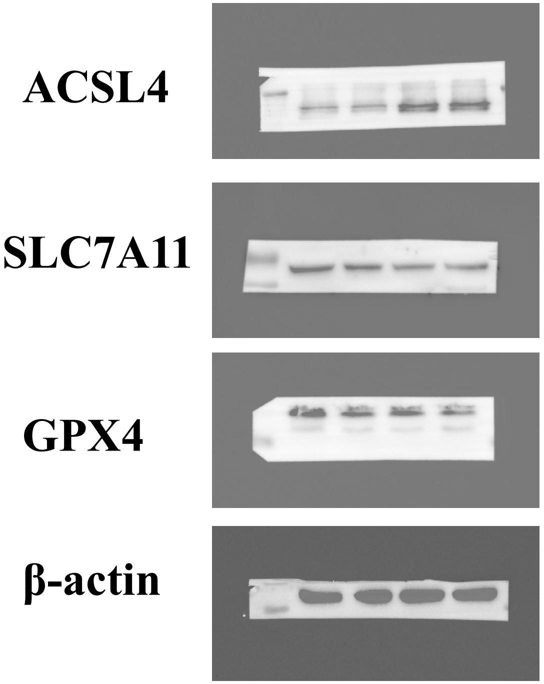

Figure 3F

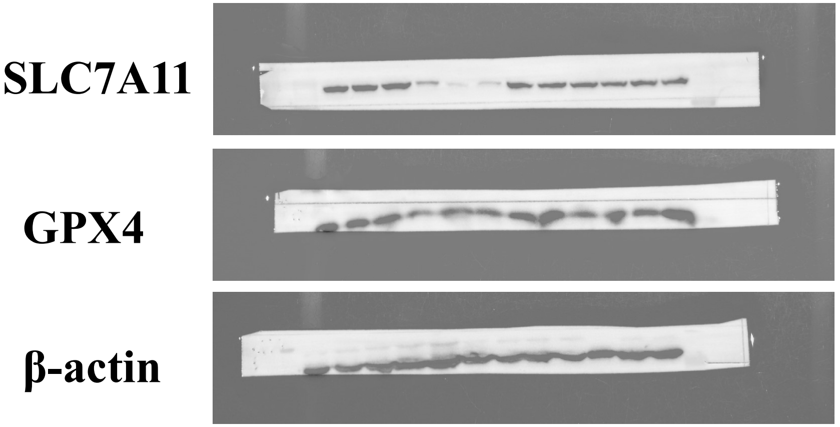

Figure 4A

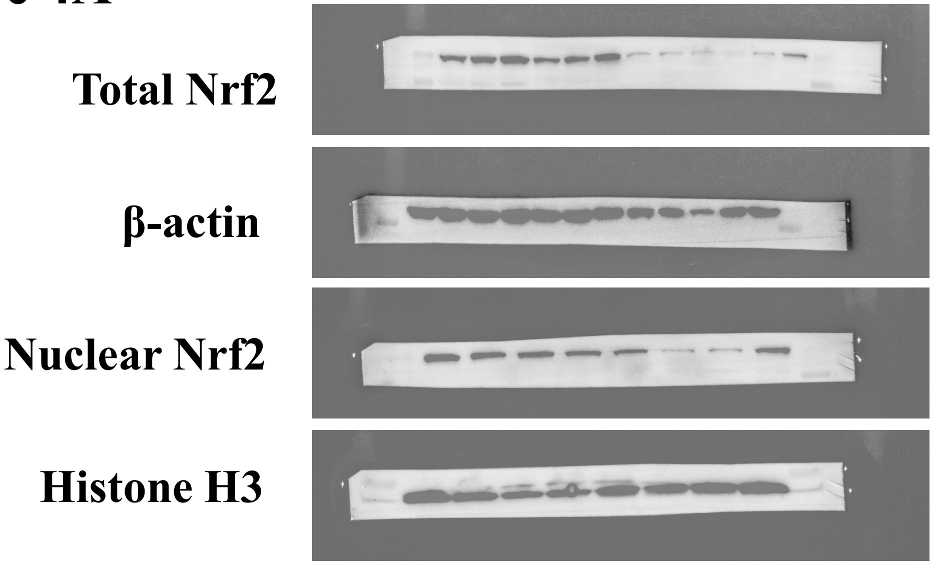

Figure 4C

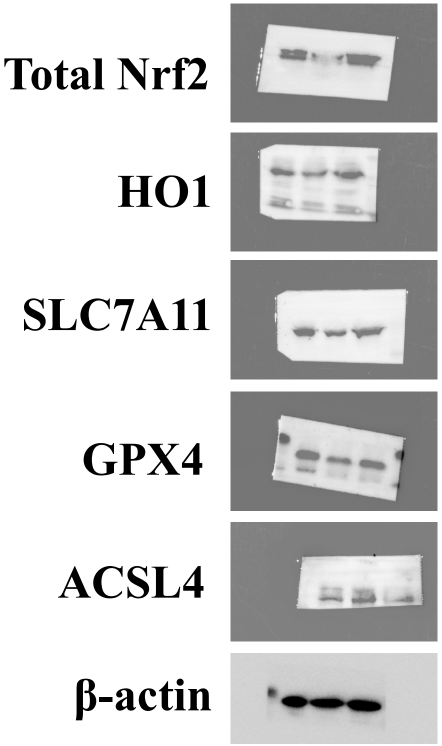

Figure 5D

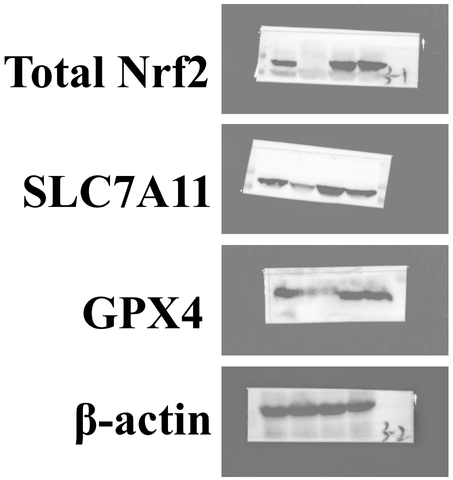

Figure 6A

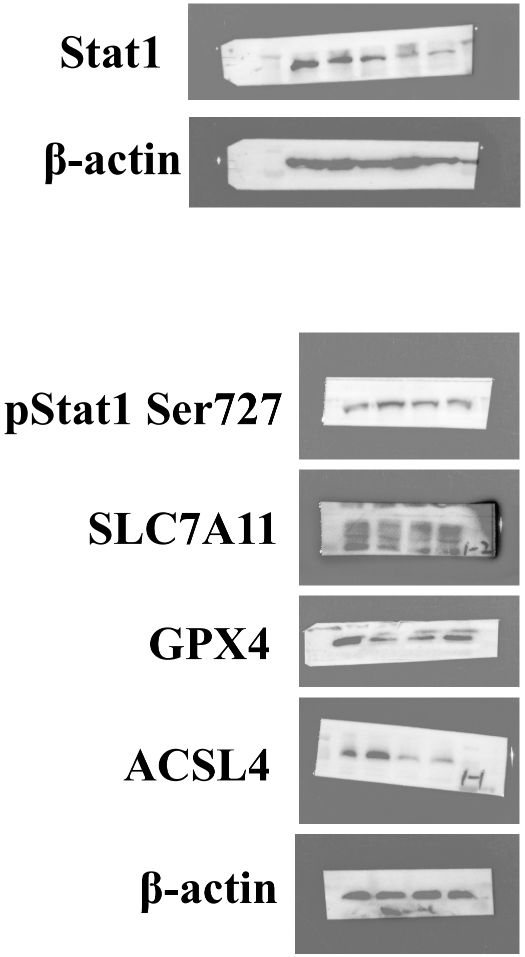

Figure 7A

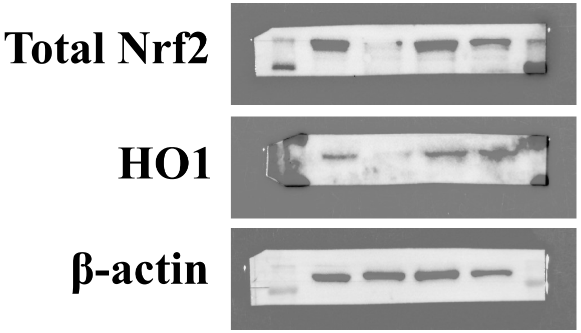

Figure 7B

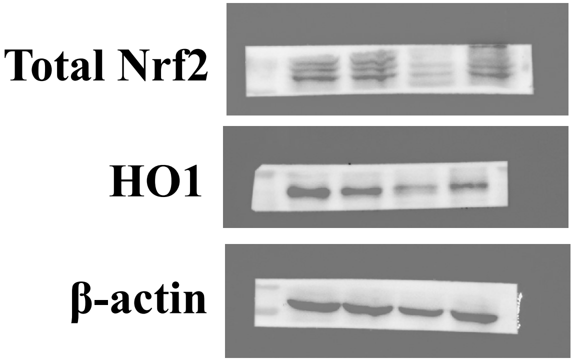

Figure 8A

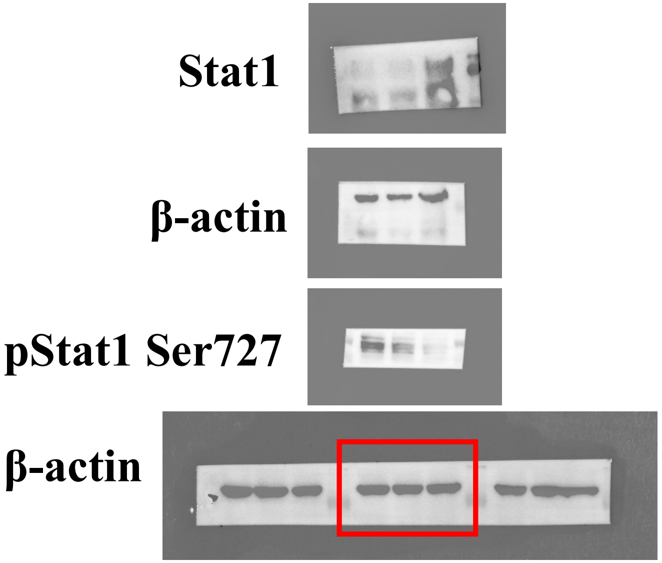

Figure 8B

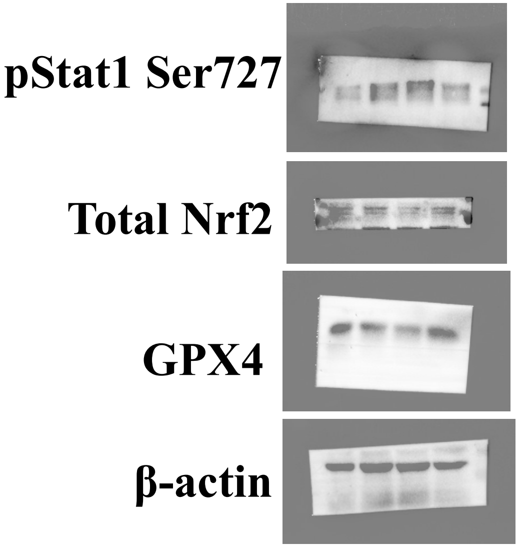

Figure 9F

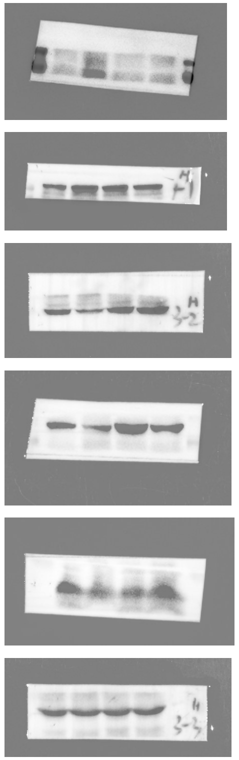

Supplementary Figure 4A

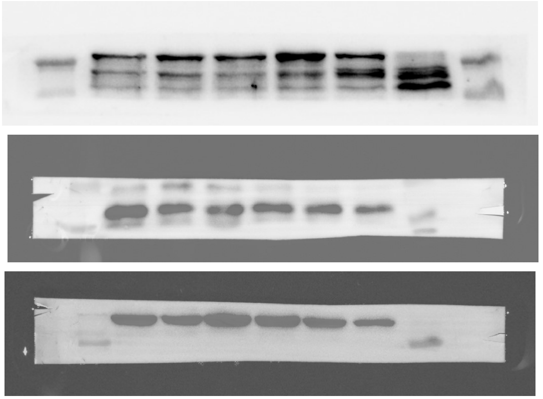

Supplementary Figure 4B

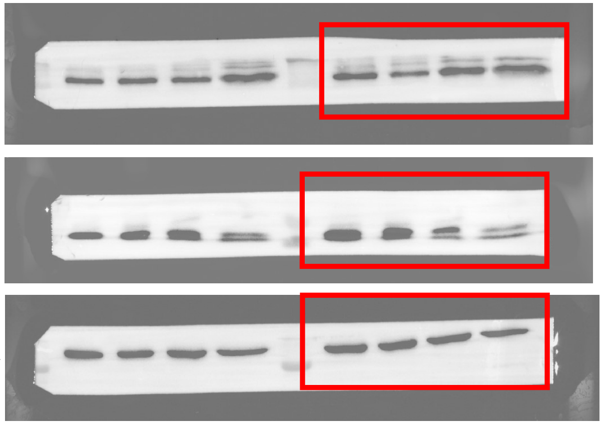

Supplementary Figure 6A

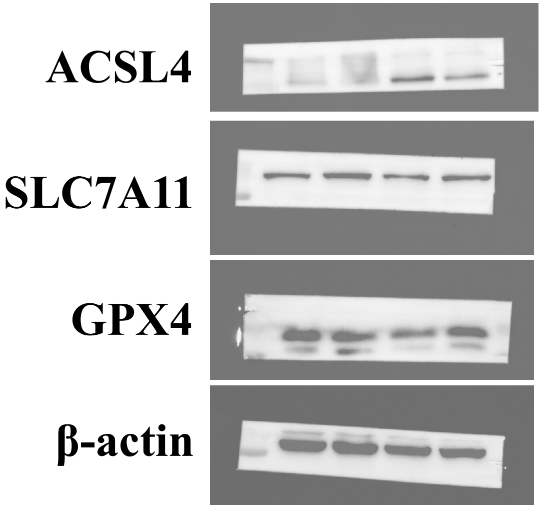

Supplementary Figure 7A

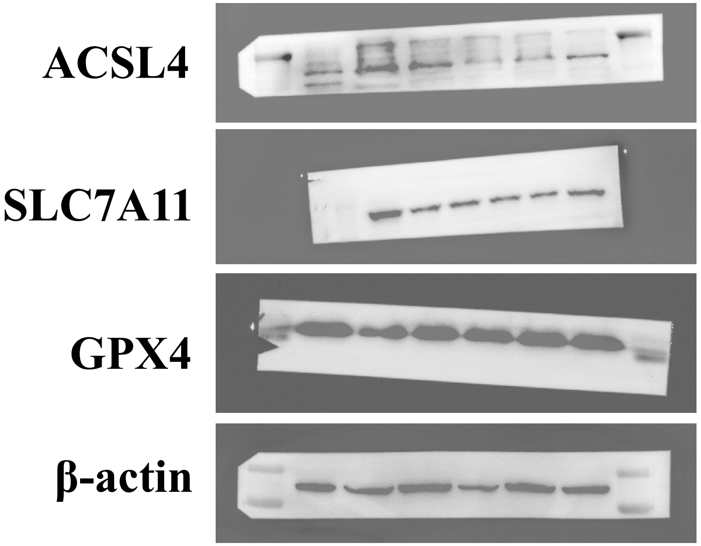

Supplementary Figure 8A

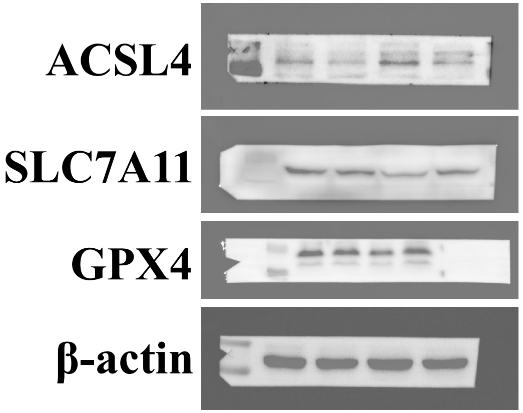

Supplementary Figure 10B

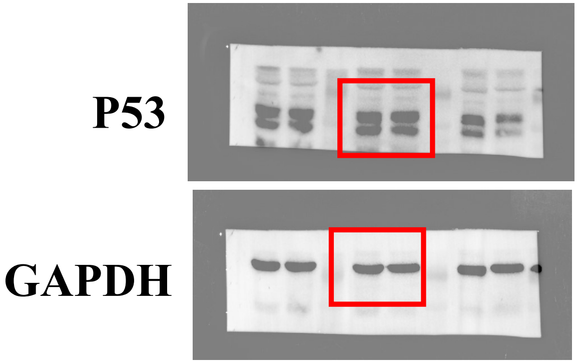

Supplementary Figure 10C

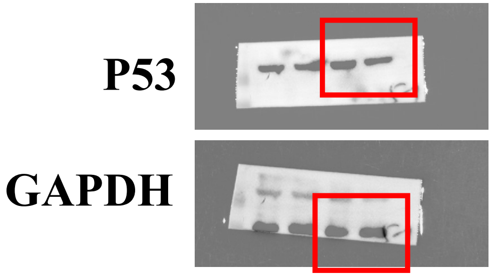

Supplementary Figure 10D

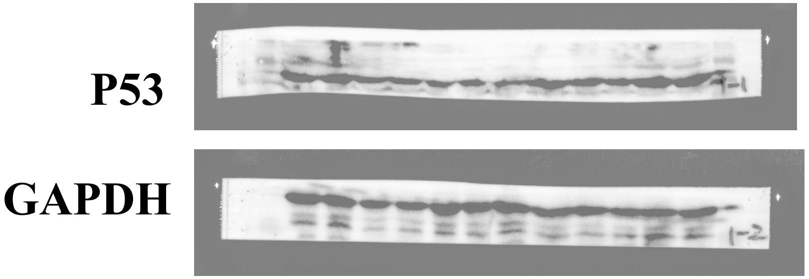

Supplementary Figure 11B

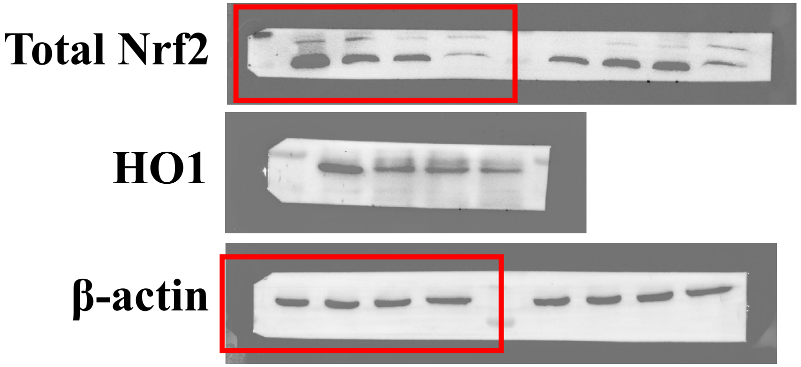

Supplementary Figure 11C

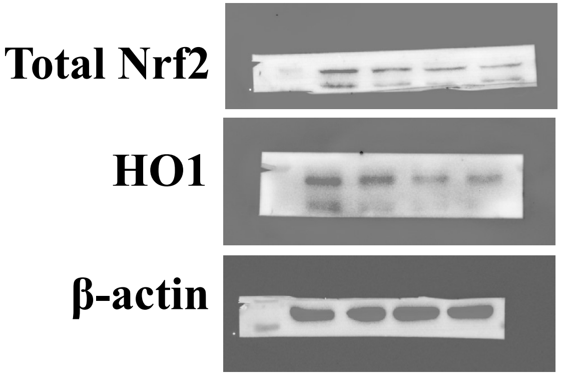

Supplementary Figure 12A

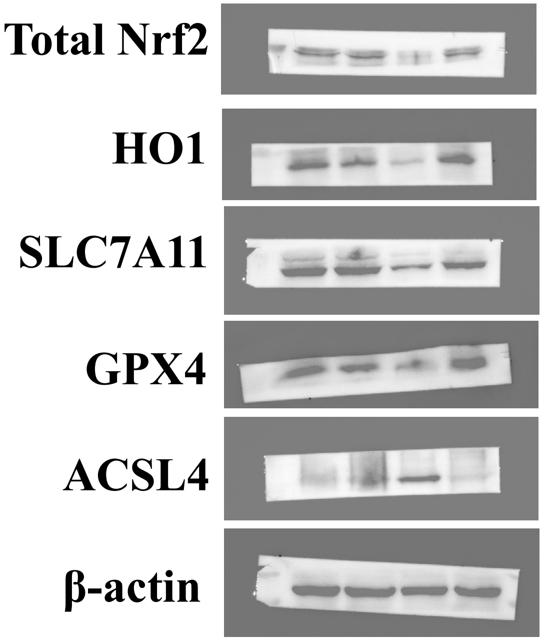

Supplementary Figure 13B

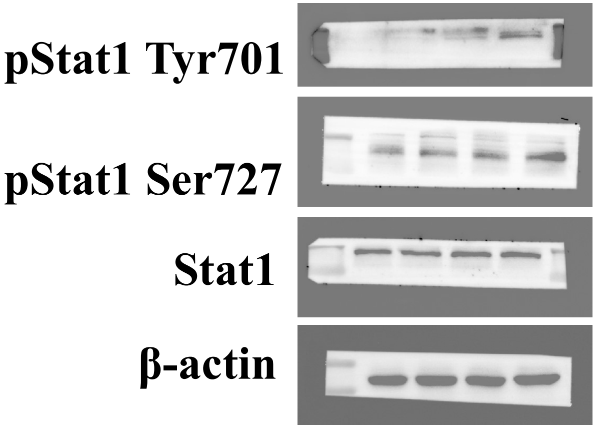

Supplementary Figure 13C

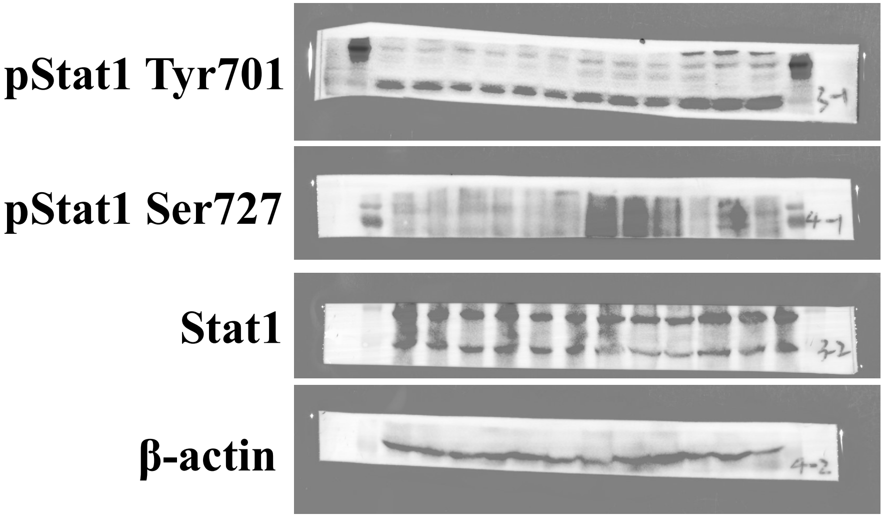

Supplementary Figure 14A

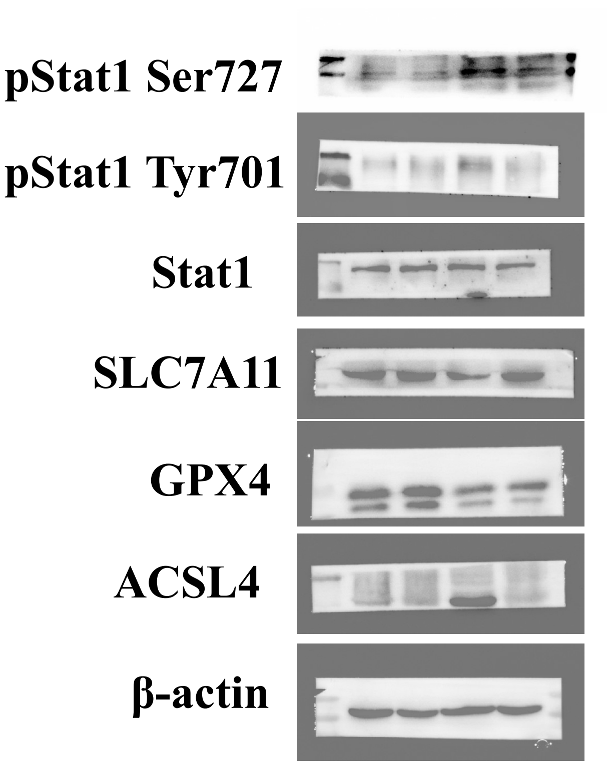

Supplementary Figure 17A

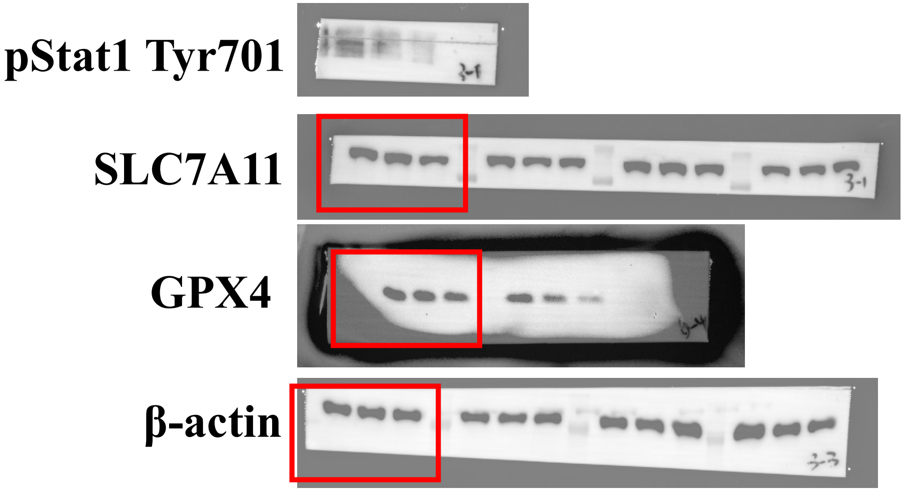

Supplementary Figure 17C

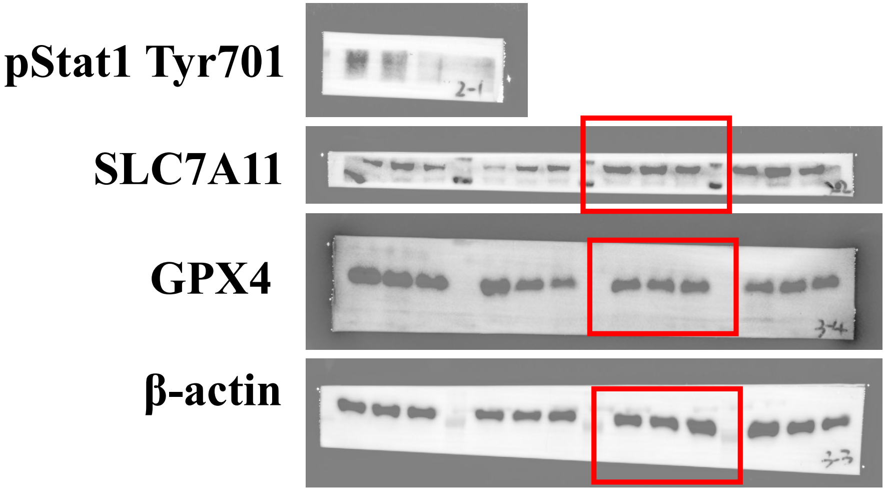

Supplementary Figure 17E

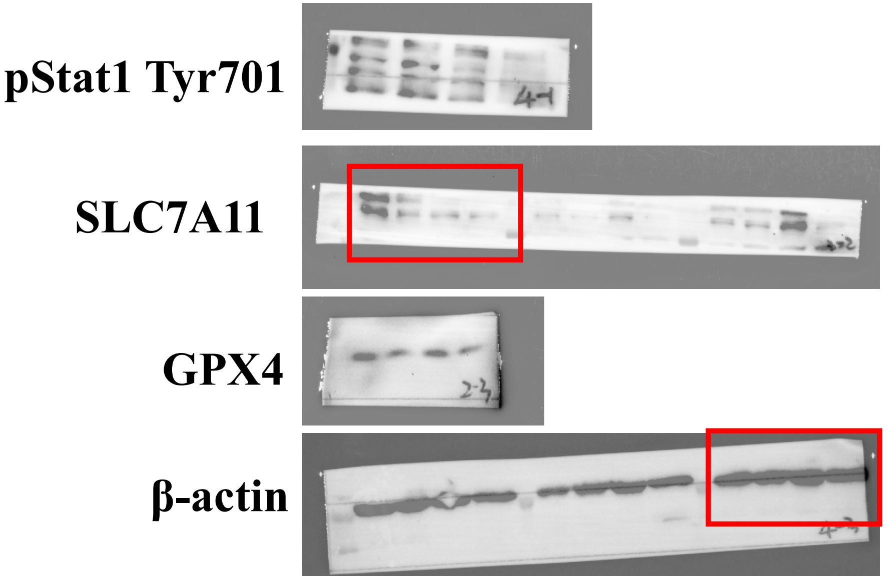

Supplementary Figure 20A

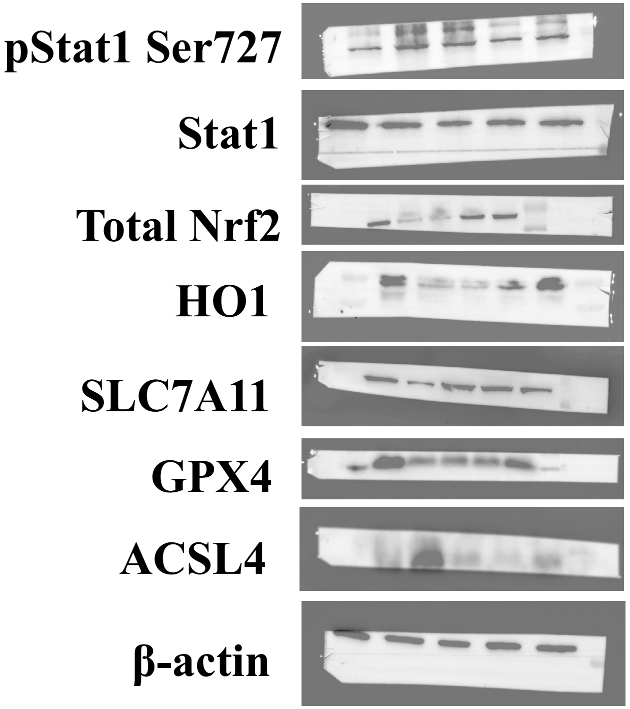

Supplementary Figure 21A

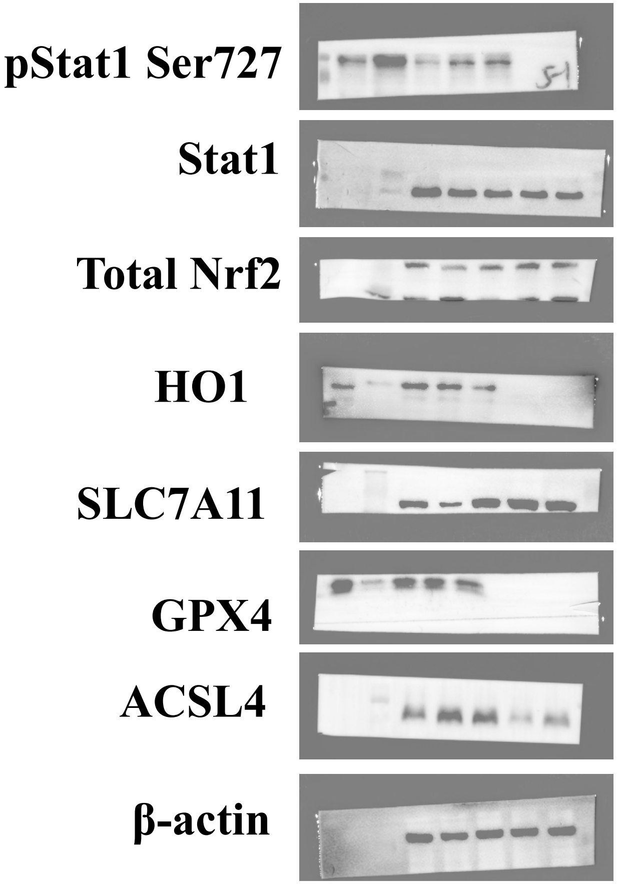

Supplementary Figure 22A

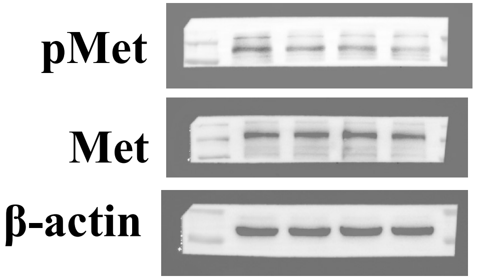

Supplementary Figure 22C

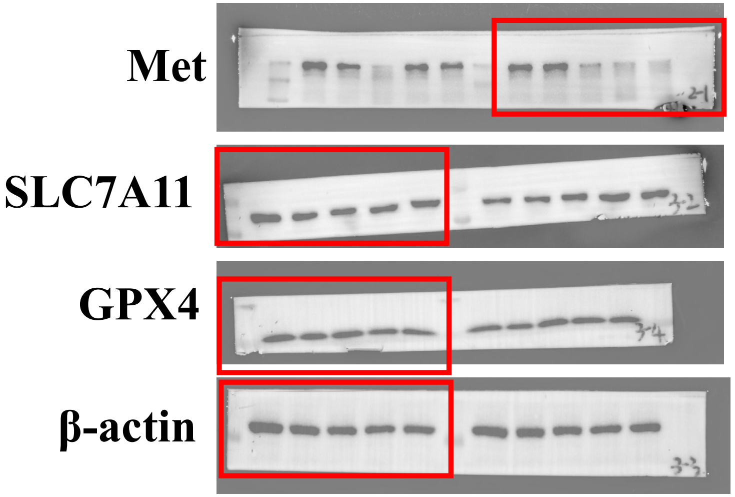

Supplementary Figure 22E

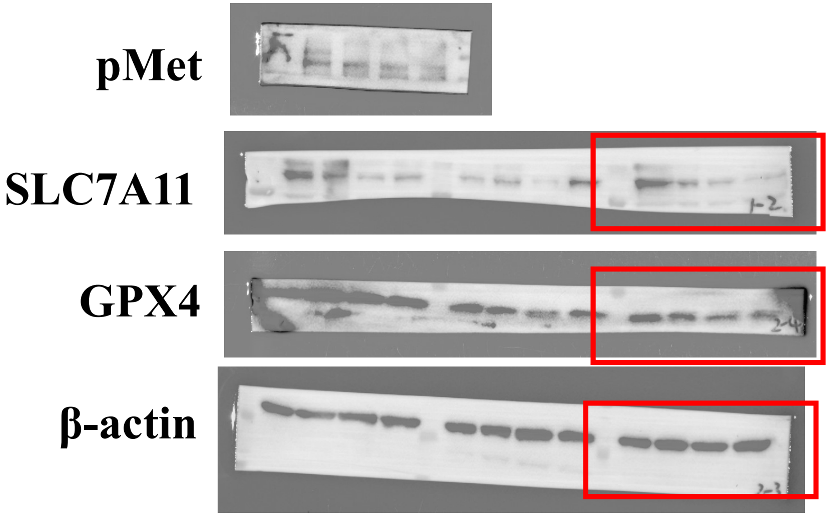

Supplementary Figure 23A

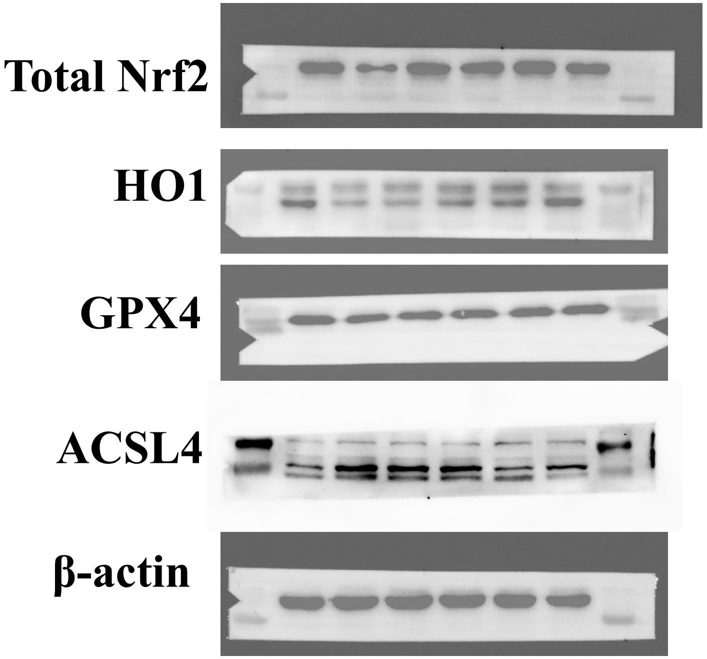

Supplement: Supplementary file 2 — Original Western Blots [file 41419_2024_6993_MOESM2_ESM.pdf]
